# Endothelial SLC25A13 links glutamine metabolism to angiogenesis

**DOI:** 10.1038/s44321-026-00524-3

**Published:** 2026-09-26

**Authors:** Abhishek Subramanian, Anne Cuypers, Patricia Yagüe, Mathias Declercq, Joris Souffreau, Pieter-Jan Coolen, Liliana Sokol, Anh-Co Truong, Mila Borri, Koen Veys, Sabine Wyns, Els Gils, Stefan Vinckier, Katleen Brepoels, Ling Jin, Huberte Moreau, Mira Mousa, Bart Ghesquière, Baharak Hosseinkhani, Guy Eelen, Mieke Dewerchin, Massimiliano Mazzone, Peter Carmeliet

**Affiliations:** 1 Laboratory of Angiogenesis and Vascular Metabolism, Center for Cancer Biology (CCB), VIB and Department of Oncology, Leuven Cancer Institute (LKI), KU Leuven, Leuven, Belgium; 2 Department of Biotechnology, Indian Institute of Technology Hyderabad, Telangana 502284, India; 3 Center for Biotechnology, Khalifa University of Science and Technology, Abu Dhabi, UAE; 4 Metabolomics Expertise Center, Center for Cancer Biology (CCB), VIB and Department of Oncology, Leuven Cancer Institute (LKI), KU Leuven, Leuven, Belgium; 5 Laboratory of Tumor Inflammation and Angiogenesis, Center for Cancer Biology (CCB), VIB and Department of Oncology, Leuven Cancer Institute (LKI), KU Leuven, Leuven, Belgium

**Keywords:** Metabolism, Vascular Biology & Angiogenesis

## Abstract

Attention to exploring endothelial cell (EC) metabolism is increasing, but a holistic perspective on the metabolic adaptations of ECs during angiogenic proliferation is still missing. Here, we use a previously in-house developed computational EC-adapted genome-scale metabolic model that recapitulates known metabolic features in proliferating ECs to predict novel pathways central to biomass synthesis. We unveil the metabolic function of the glutamate–aspartate antiporter SLC25A13, previously overlooked in ECs, as a key regulator of glutamine utilization and provide evidence regarding its contribution to the generation of cholesterol and other biomass precursors. To our knowledge, predominant SLC25A13-linked utilization of glutamine for cholesterol synthesis has not been reported in other cell types in prior literature. Silencing of *SLC25A13* in ECs reduced proliferation and vessel sprouting in vitro as well as in a choroidal neovascularization mouse model. These insights underscore the particular characteristics of EC metabolism and may warrant further evaluation of SLC25A13 as a putative new anti-angiogenic target.

## Synopsis

SLC25A13 plays a pivotal role in angiogenesis through its regulatory function in glutamine metabolism and biomass synthesis in endothelial cells (ECs). This highlights SLC25A13 as a promising target for the development of novel anti-angiogenic therapies.

- Silencing of *SLC25A13* in ECs significantly impairs biomass synthesis, thereby limiting the availability of essential precursors required for new blood vessel formation.
- Silencing of *SLC25A13* leads to a pronounced reduction in proliferation, migration, and sprouting in vitro, as well as diminished angiogenic responses in vivo.
- SLC25A13 inhibition may serve as a potential therapeutic target in pathological conditions characterized by dysregulated angiogenesis.

## Abstract (web summary)

SLC25A13 plays a pivotal role in angiogenesis through its regulatory function in glutamine metabolism and biomass synthesis in endothelial cells (ECs). This highlights SLC25A13 as a promising target for the development of novel anti-angiogenic therapies.

## Graphical abstract

**Figure:**
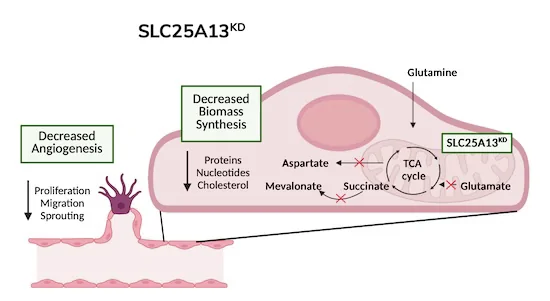

The paper explained **Problem**

Abnormal angiogenesis underlies several major diseases, including cancer and eye disorders. ECs depend on appropriate metabolism for their angiogenic activity, making EC metabolism an attractive therapeutic target.

**Results**

We identified the glutamate–aspartate antiporter SLC25A13, previously overlooked in endothelial cells (ECs), as a key regulator in EC metabolism. SLC25A13 coordinates glutamine utilization with the tricarboxylic acid cycle to support the synthesis of biomass precursors, particularly those of the mevalonate pathway.

Our findings suggest that SLC25A13 plays a distinct metabolic role in ECs compared with other proliferating cell types. Whereas previous studies have primarily linked SLC25A13 to coupling glycolysis with oxidative phosphorylation, our data indicate that in ECs it is essential for directing glutamine utilization towards the synthesis of biomass precursors, including mevalonate and cholesterol, highlighting a metabolic adaptation that appears to be particularly important during angiogenic proliferation. Accordingly, silencing *SLC25A13* in ECs reduced angiogenesis and cell proliferation in vitro, as well as in a choroidal neovascularization mouse model in vivo.

**Impact**

Our findings reveal that silencing of *SLC25A13* significantly impairs angiogenesis and cell proliferation, supporting its further evaluation as a potential anti-angiogenic therapeutic target, particularly in diseases characterized by aberrant vascularization and pathological cell proliferation.

## Introduction

Endothelial cells (ECs) line blood vessels and perform various functions such as angiogenesis, regulation of vessel permeability, supply of nutrients and removal of waste, leukocyte transmigration, hemostasis, vasomotor tone, and immune modulation (Aird, 2007). ECs remain mostly quiescent in healthy adults but are rapidly activated in response to angiogenic stimuli produced by neighboring tissues that require vascularization (Falkenberg et al, 2019). Activation of ECs leads to a phenotypic switch in which the quiescent ECs differentiate to migratory tip cells that move towards the angiogenic stimulus, and to proliferating stalk cells that elongate the sprout, a process referred to as sprouting angiogenesis. In tumors, sprouting angiogenesis is often considered the key mode of vascularization and has become a target for anti-angiogenic therapy (AAT) (Carmeliet and Jain, 2011). However, the efficacy and clinical benefit of AAT is still limited (Bergers and Hanahan, 2008; Ellis and Hicklin, 2008; Welti et al, 2013). In addition to angiogenic stimuli, including vascular endothelial growth factor (VEGF, a target of current AAT), EC metabolism has been recognized as a key driver of tumor angiogenesis, since ECs need to adapt their metabolism to optimally allocate metabolic resources required for angiogenesis (De Bock et al, 2013; Eelen et al, 2018; Schoors et al, 2015). In recent years, EC metabolism has gained increasing attention as a novel therapeutic target to normalize the abnormal, leaky, and tortuous tumor blood vessels. A key characteristic of ECs is their preference of glycolysis over oxidative phosphorylation  even under oxygen-abundant conditions, to produce ATP rapidly (De Bock et al, 2013). Besides ECs, only a few non-cancerous, proliferating cells exhibit such a Warburg-like phenomenon (Abdel-Haleem et al, 2017). ECs adapt their metabolism in a unique manner, different from many other healthy and cancer cell types (Li et al, 2019). For instance, the oxidation of fatty acids along with the use of anaplerotic substrates for nucleotide synthesis (Schoors et al, 2015), serine biosynthesis for heme production (Vandekeere et al, 2018), glutamine as an essential nitrogen source for asparagine production (Huang et al, 2017) and the critical role of oxidative phosphorylation to maintain NAD/NADH ratio for aspartate biosynthesis rather than ATP production (Diebold et al, 2019) underscore the unique nature of proliferative EC metabolism. Other metabolic pathway changes in ECs are shared amongst other cell types (Li et al, 2019). In any living cell, metabolic pathways do not function independently but rather couple with other pathways to produce the necessary macromolecular needs of the cell. A unified understanding of the flexible metabolic adaptations of ECs for different substrates is still missing. Such a perspective can contextualize the use of other pathways in the light of existing metabolic knowledge and enable the discovery and prioritization of new anti-angiogenic targets.

Genome-scale metabolic models (GEMs) present a mathematical framework for representing cellular metabolism. They enable a system-wide analysis of existing gene (enzyme) and reaction knowledge of the metabolism and extracellular metabolite conditions. Additionally, GEMs integrate diverse biological data (including gene, transcript, protein, and metabolic reactions) to predict optimal metabolic pathways for various cellular purposes, under defined cellular conditions (O’Brien et al, 2015). Generic human metabolic reconstructions, subsequently tailored using condition-specific omics data in ECs, have been instrumental in uncovering the significance of fatty acid oxidation as a pathway that regulates EC permeability (Patella et al, 2015). Recently, we developed in-house a GEM customized for proliferating ECs by utilizing bulk and single-cell transcriptomics data and experimentally validated the EC-GEM-predicted essential role of cholesterol and collagen biosynthesis in choroidal neovascularization (Rohlenova et al, 2020), demonstrating the power of using GEMs in the exploration of EC metabolism. This EC-specific GEM can thus be exploited in-depth to identify metabolic pathways that enable ECs to optimally use metabolic sources for biomass synthesis to support EC proliferation and angiogenesis.

In this study, we used the EC-specific GEM to further identify which pathways are crucial for biomass synthesis by ECs. By performing flux-based computational analyses in the EC-specific GEM, coupled with gene-level confirmatory perturbation experiments, we discover an essential role for the mitochondrial glutamate–aspartate carrier protein SLC25A13 (also called AGC2), a component of the malate–aspartate shuttle (MAS), in supporting biomass synthesis in ECs. AGCs together with the 2-oxoglutarate carrier (OGC) play a crucial role in the transport of NADH reducing equivalents from the cytosol to the mitochondrial matrix as a component of the MAS (Amoedo et al, 2016). Using phenotypic and metabolic assays, we establish SLC25A13 as responsible for the GEM-predicted aspartate–glutamate antiport function that coordinates glutamine degradation with the TCA cycle to generate biomass precursors (nucleotide, protein, and cholesterol synthesis). In ECs, SLC25A13 is decoupled from glycolysis and ATP production, an observation contrary to previous reports in fibroblast and cancer cell lines (Rabinovich et al, 2020). Additionally, we report for the first time the predominant utilization of glutamine as a carbon source for cholesterol synthesis and the essential function of SLC25A13 in influencing cellular cholesterol levels, a finding not documented in any other cell type to date. Based on its central role in the generation of multiple biomass precursors, SLC25A13 might deserve further attention as a possible therapeutic candidate for diseases/disorders involving pathological sprouting angiogenesis, even more so if SLC25A13 can be inhibited selectively in ECs.

## Results

### EC-GEM predicts known and new utilization pathways in biomass synthesis

**Figure EV1:**
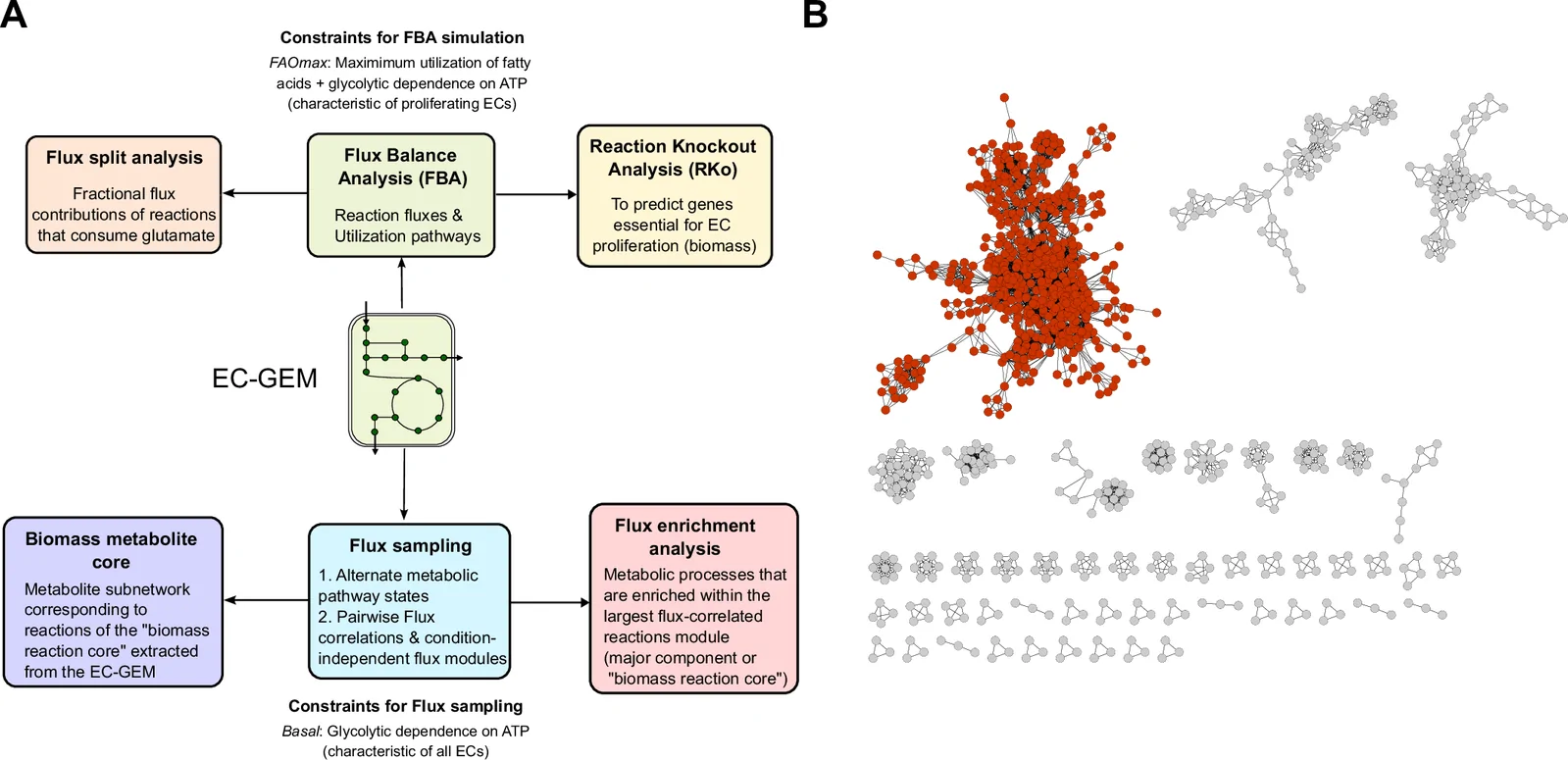
Endothelial cell genome-scale metabolic model workflow. (**A**) Workflow of the analyses performed using the endothelial cell genome-scale metabolic model (EC-GEM). (**B**) Flux correlation analysis in the EC-GEM. The network plot shows 1064 flux-correlated reactions distributed into multiple modules (groups). The nodes (balls) represent the reactions, and the sticks (edges) represent the presence of a correlation between the nodes. The red-colored motif represents the biomass reaction core that contains 548 flux-correlated reactions belonging to pathways required for biomass synthesis in ECs. The gray-colored motifs are smaller connected components of flux-coupled reactions enlisted in Dataset EV1.

**Figure 1:**
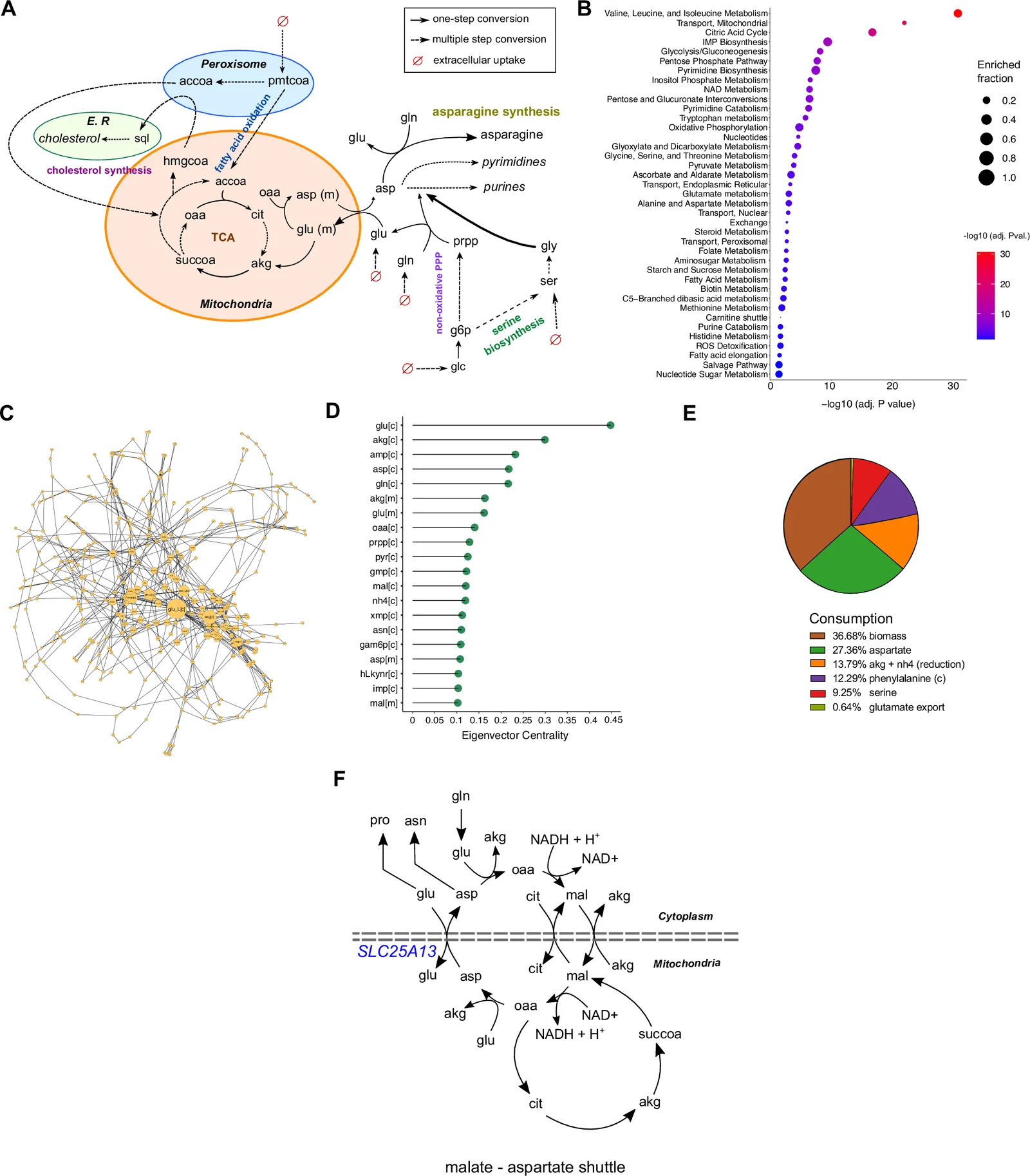
Genome-scale metabolic modeling predicts mitochondrial transport to be essential for biomass synthesis. (**A**) The EC-GEM recapitulates canonical metabolic pathways activated during EC proliferation. (**B**) Flux enrichment analysis displaying the enriched pathways in the “biomass reaction core” subnetwork. The dot size represents the contribution of the enriched fraction; the bigger the dot, the higher the contribution of the process. −Log10 (adj. Pval.), red indicates highly upregulated. (**C**) Biomass metabolite core: The network plot shows 371 metabolites that were extracted from the reactions of the biomass reaction core in (**B**). The size of the nodes (balls) corresponds to the eigenvector centrality of the metabolite within the network. (**D**) Eigenvector centrality ranking: Lollipop plot showing the sorted ranking of metabolites based on eigenvector centrality. Eigenvector centrality represents the importance of the metabolite based on the influence it can have on other nodes in the network. (**E**) Percentage consumption of total glutamate flux for the production of other metabolites. (**F**) The malate aspartate shuttle: The glutamate aspartate antiporter SLC25A13 is shown in blue color. accoa acetyl-coA, akg α-ketoglutarate, asp aspartate, cit citrate, E.R. endoplasmic reticulum, g6p glucose-6-phosphate, glc glucose, gln glutamine, glu glutamate, gly glycine, hmgcoa hydroxymethylglutaryl-coA, (m) mitochondrial, mal malate, oaa oxaloacetate, pmtcoa palmitoyl-coA, pro proline, prpp phosphoribosylpyrophosphate, ser serine, sql squalene, succoa succinyl-coA. Source data are available online for this figure.

We used our previously in-house developed endothelial cell genome-scale metabolic model (EC-GEM) to recapitulate known metabolic features and to predict new characteristics of metabolism in proliferating endothelial cells (ECs) (Rohlenova et al, 2020). We used two sets of constraints to identify unrecognized pathways for biomass synthesis (as a proxy for EC proliferation): (i) FAO_max_: a constraint that specifically identifies a flux space to maximize the utilization of fatty acids for aspartate synthesis (taken from our previous study (Rohlenova et al, 2020)); and (ii) basal metabolism: a constraint that allows flexible adaptation of ECs to different carbon and nitrogen sources. By performing flux balance analysis (FBA) on the EC-GEM using FAO_max_ constraints (see “Methods”, Fig. EV1A; Dataset EV1), utilization pathways of different environmental metabolites toward synthesis of biomass precursors were inferred from the estimated reaction fluxes (Fig. 1A). The EC-GEM was able to capture the utilization of glucose into glycolysis, the pentose phosphate pathway and the serine biosynthesis pathways (Dumas et al, 2020; Falkenberg et al, 2019) (Fig. 1A). These pathways mainly lead to the synthesis of phosphoribosyl pyrophosphate (*prpp*) and glycine (*gly*), known precursors of purine and/or pyrimidine synthesis (Fig. 1A). Similar to a previous observation (Labuschagne et al, 2014), cellular serine was predicted to be a sum of serine produced through biosynthesis and extracellular uptake. Analogous to previous experimental observations (Huang et al, 2017; Kim et al, 2017), the EC-GEM recapitulates the established functions of glutamine in synthesizing glutamate, aspartate, asparagine, and nucleotide precursors. Palmitoyl-CoA (pmtcoa) is used for both mitochondrial and peroxisomal β-oxidation to produce acetyl-CoA (accoa) and other TCA intermediates (Schoors et al, 2015) (Fig. 1A). Overall, the EC-GEM correctly predicted previously characterized metabolic pathways in ECs, further establishing its predictive potential (RKo).

By performing an in silico reaction knockout analysis (RKo) in the EC-GEM (Heirendt et al, 2019), metabolic genes crucial for biomass generation were predicted. A total of 358 essential reactions were predicted by this analysis. To validate our RKo analysis, a ground truth dataset was curated and compared with EC-GEM RKo predictions. This ground truth dataset contained the essentiality information of 34 metabolic genes that were experimentally tested for their impact on proliferation in previous studies (Rohlenova et al, 2020) (“Methods”, Dataset EV1). The metabolic genes (and their associated reactions) in the ground truth dataset were termed “essential” if they explicitly reduced proliferation as a result of a metabolic defect in biomass production; otherwise, they were termed “non-essential”. The essentiality predictions for these 34 metabolic genes from the EC-GEM RKo were compared with the ground truth essentiality information. With the FAO_max_ constraint, EC-GEM achieved a high sensitivity of 78.5% (the rate at which actual essential genes are also predicted from the EC-GEM to be essential) and a high accuracy, as indicated by the F1-score of 0.88 in predicting true essential genes (reactions) (Dataset EV1).

To predict alternative optimal pathways for maximizing biomass synthesis, we conducted a flux sampling simulation of the EC-GEM using basal constraints (Fig. EV1A). This analysis generated random samples of optimal flux distributions representing alternative pathway states for maximal biomass synthesis (Haraldsdottir et al, 2017). Subsequently, a flux-based correlation analysis of reactions was performed on these random samples to identify pairs of reactions that are coupled by flux. The flux-correlated reactions were represented as a correlation network to identify flux-coupled reaction modules (see Methods). This analysis identified 1,064 flux-correlated reactions that are distributed into multiple modules (Dataset EV1; Fig. EV1B). The major connected component (Fig. EV1B; Dataset EV1 (component 1)) accounted for 51.5% (548 reactions) of the total flux-correlated reactions.

Flux enrichment analysis demonstrated that this major connected component (Fig. EV1B (module with red-colored nodes)) was overrepresented in multiple known metabolic subsystems and processes such as glycolysis, pentose phosphate pathway, oxidative phosphorylation, nucleotide salvage, purine/pyrimidine synthesis and amino acid catabolic pathways related to nucleotide and protein synthesis in ECs (Fig. 1B). We therefore labeled this major component as the “biomass reaction core”. Extraction of the metabolite network associated with this biomass reaction core and subsequent evaluation for the most influential metabolite within this associated network revealed glutamate to be the most central metabolite (Fig. 1C,D), similar to a previous study (Oberkersch et al, 2022).

Flux split analysis with the FAO_max_ constraints also revealed that 27.4% of glutamate is used to exchange for cellular aspartate synthesis in the EC-GEM (Fig. 1E). Previous studies in non-endothelial cell types demonstrated the importance of mitochondrial aspartate synthesis from glutamate in general cell proliferation (Alkan et al, 2018; Ma et al, 2023). Mitochondrial aspartate synthesis involves cross-compartmental conversion of glutamate and aspartate, mediated by mitochondrial glutamate–aspartate antiporters. These are part of the malate–aspartate shuttle, which is involved in the translocation of electrons across the mitochondrial inner membrane (Fig. 1F). Interestingly, mitochondrial metabolite transport was one of the highly enriched metabolic subsystems in the biomass reaction core (Fig. 1B). The mitochondrial aspartate–glutamate antiport reaction (*ASPGLUm*) was also predicted to be essential by the RKo analysis (Dataset EV1). The predicted direction of metabolic flux through *ASPGLUm* also indicated an export of aspartate from the mitochondria to the cytoplasm, supporting the predicted consumption of glutamate for aspartate synthesis (Dataset EV1). The EC-GEM gene-protein-reaction association links two antiporter isoforms, SLC25A12 and SLC25A13, to the *ASPGLUm* reaction responsible for mitochondrial aspartate export.

### Loss of SLC25A13 function impairs EC proliferation and angiogenesis

**Figure EV2:**
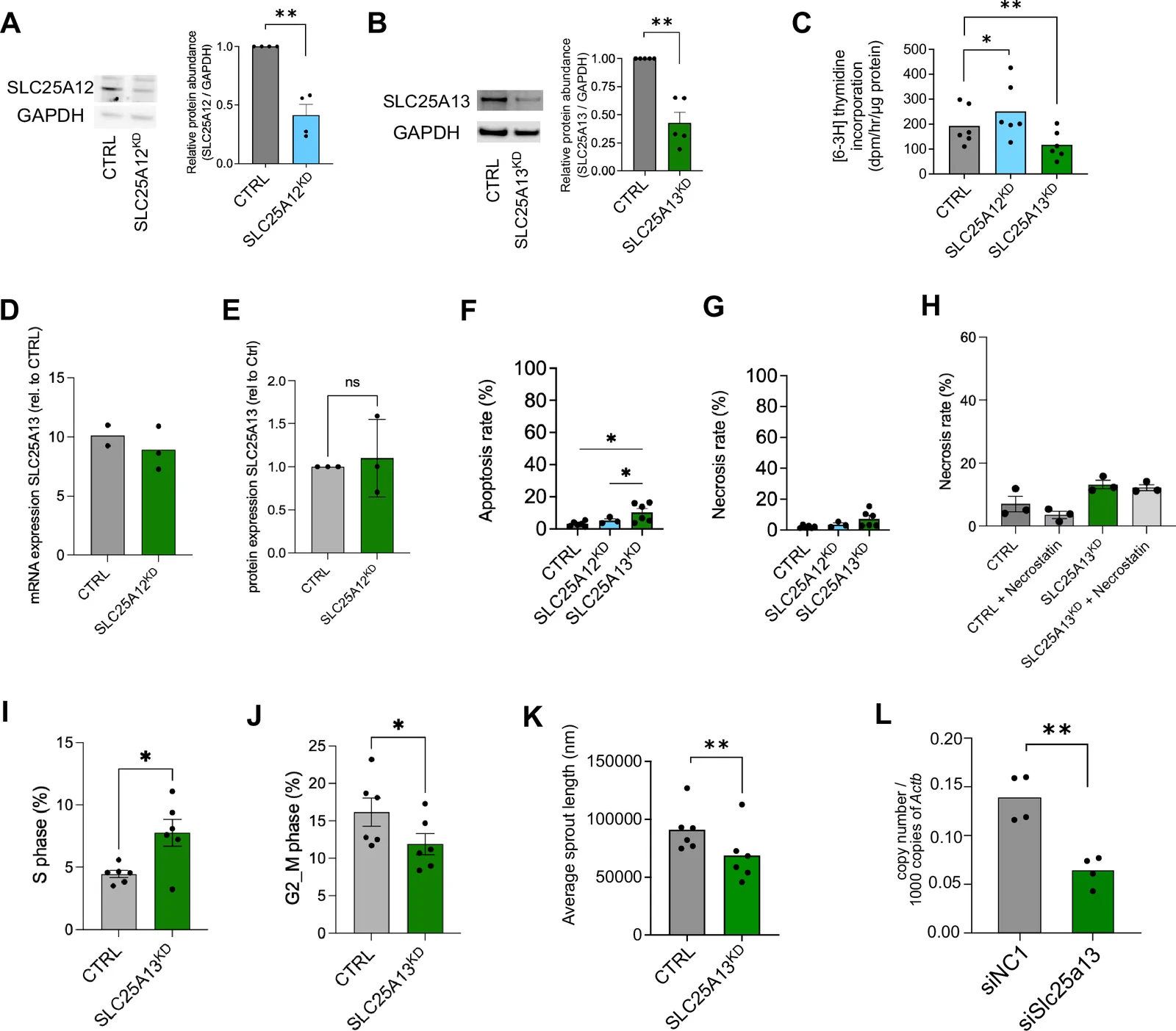
Functional characterization of *SLC25A12* and *SLC25A13* knockdown in endothelial cells. (**A**) Representative immunoblot and densitometric quantification of SLC25A12 protein levels in CTRL and SLC25A12^KD^ ECs (expressed relative to control) (*n* = 4). GAPDH was used as a loading control. (**B**) Representative immunoblot and densitometric quantification of SLC25A13 protein levels in CTRL and SLC25A13^KD^ ECs (expressed relative to control) (*n* = 4 and *n* = 5, respectively). GAPDH was used as a loading control. (**C**) Proliferation ([6-^3^H] thymidine incorporation into DNA) of CTRL, SLC25A12^KD^, and SLC25A13^KD^ ECs (*n* = 6). These results were obtained in the same experiment as the data shown in Fig. 2A and therefore show the same results for the CTRL. The results for the SLC25A13^KD^ bar have been repeated for an easy comparison. (**D**) Quantification of *SLC25A13* mRNA expression levels in CTRL and SLC25A12^KD^ ECs (expressed relative to control) (*n* = 2–3). (**E**) Densitometric quantification of SLC25A13 protein levels in CTRL and SLC25A12^KD^ ECs (expressed relative to control) (*n* = 3). (**F**) Quantification of apoptosis with Annexin/PI test (*n* = 3–6). (**G**) Quantification of necrosis with Annexin/PI test. (**H**) Quantification of necroptosis with Annexin/PI test (*n *= 3–6). (**I**) Quantification of cell cycle S-phase (*n* = 6). (**J**) Quantification of cell cycle G2_M-phase (*n* = 6). (**K**) Spheroid sprouting in mitomycin C-treated mitotically inactivated EC: sprout length (nm) of sprouting CTRL and SLC25A13^KD^ EC spheroids (*n* = 6). (**L**) Transcript levels of *Slc25a13* in ECs isolated from siNC1 or siSLC25A13 treated neovascularized choroids (*n* = 4). Data are means ± SEM; *P < 0.05, **P < 0.01 by one-sample *t* test and Wilcoxon test (**A**, **B**); a two-tailed paired *t* test (**C**, **E**, **F**, **G**, **I**–**K**); by a one-way ANOVA (**H**); unpaired *t* test (**L**). Exact *P* values: (**A**) *P* = 0.0077; (**B**) *P* = 0.0037; (**C**) CTRL vs SLC25A13, *P* = 0.0015; CTRL vs SLC25A12, *P* = 0.0235; (**F**) CTRL vs SLC25A13, *P* = 0.0270; SLC25A12 vs SLC25A13, *P* = 0.0364; (**I**) *P* = 0.0179; (**J**) *P* = 0.0169; (**K**) *P* = 0.0096; (**L**) *P* = 0.0020.

**Figure 2:**
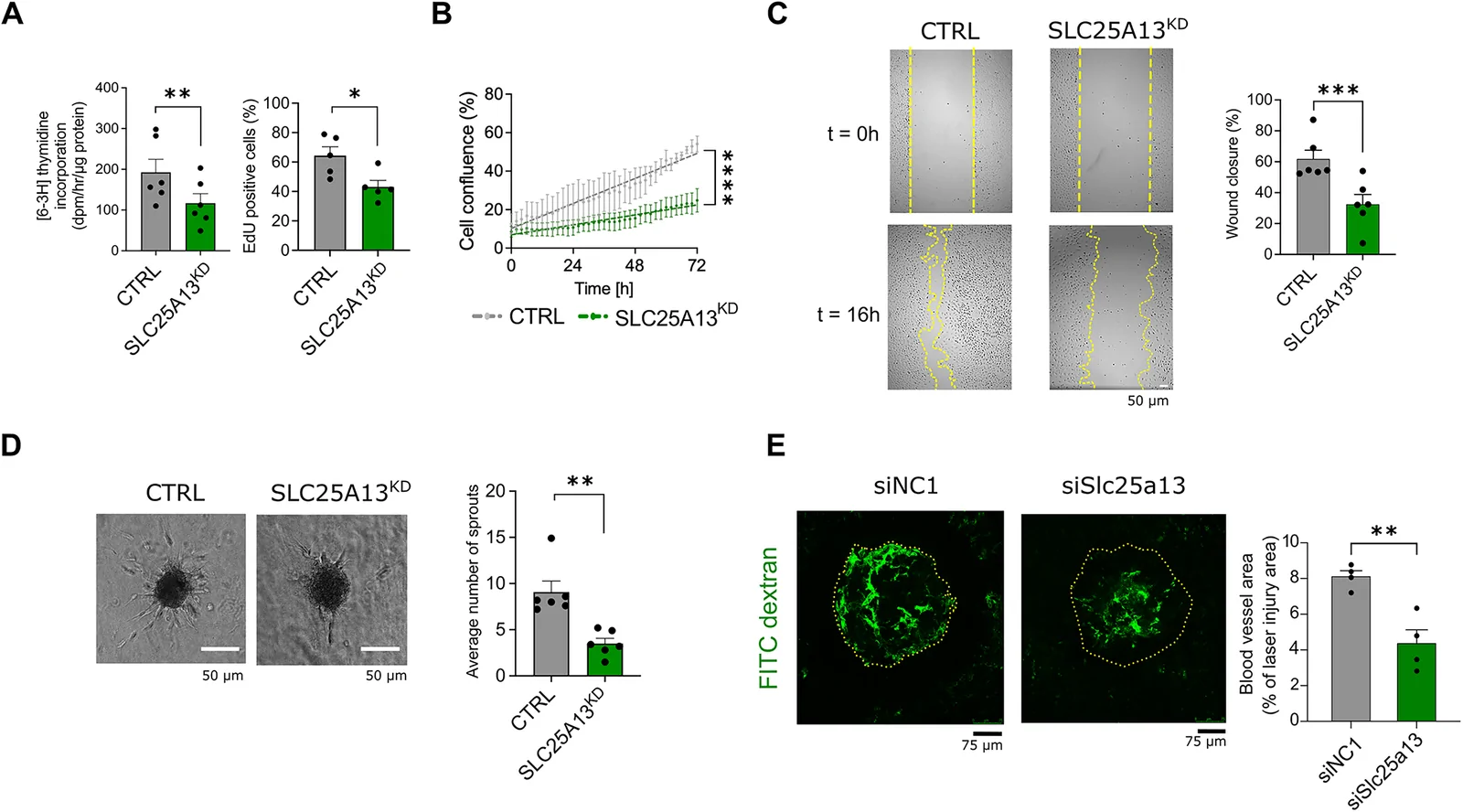
*SLC25A13* silencing affects EC proliferation, migration, and vessel sprouting. (**A**) [6-^3^H]-Thymidine and EDU incorporation into DNA in CTRL and SLC25A13^KD^ ECs (*n* = 5 and *n* = 6, respectively). (**B**) Rate of change of cell proliferation (measured as % cell confluence) with time (0–72 h). The dotted lines represent linear regression through the datapoints for each condition (*n* = 3). (**C**) Wound closure capacity (proxy of migration) of mitomycin-C-treated CTRL and SLC25A13^KD^ ECs. Left: representative microscopy images displaying the wound closure capacity at the start (t0, immediately after applying the scratch wound) and end (t16) of the experiment. Right: quantification of migration (area covered by the cells for closing the scratch wound) (*n* = 6). Scale bar: 50 μm. (**D**) Spheroid sprouting in mitomycin C-treated mitotically inactivated EC. Left: representative images of sprouting CTRL and SLC25A13^KD^ EC spheroids. Right: average number (#) of sprouts per spheroid (*n* = 6). Scale bars: 50  μm. (**E**) In vivo choroidal neovascularization upon intravitreal injection of a siNC1 (negative control) or *Slc25a13*-specific siRNA (siSlc25a13). Left: representative images of FITC-Dextran-stained (green, visualizing the neovasculature) choroidal flatmounts. Right: Quantification of choroidal neovascularization (blood vessel area; green) normalized to laser injury area (demarcated by dotted line in the images) (*n* = 4) (each datapoint represents the mean of the three mice of an independent replicate experiment). Scale bars: 75 μm. Data are means ± SEM; *P < 0.05, **P < 0.01, ***P < 0.001,****P < 0.0001 by two-tailed paired *t* test (**A**, **C**, **D**); simple linear regression analysis (**B**); two-tailed unpaired *t* test (**E**). Exact *P* values: (**A**) thymidine incorporation, *P* = 0.0015; EdU incorporation, *P* = 0.0107; (**B**) *P *< 0.0001; (**C**) *P* = 0.0006; (**D**) *P* = 0.0071; (**E**) *P* = 0.0044. CTRL – HUVECs transfected with control (nonsense scrambled) shRNA. Source data are available online for this figure.

To experimentally investigate the role of SLC25A12 and SLC25A13 in EC proliferation and angiogenesis, we performed several in vitro assays and in vivo validation experiments. We silenced *SLC25A12* or *SLC25A13* in human umbilical vein ECs (HUVECs, further named ECs) by lentiviral transduction of a construct expressing target-specific short hairpin RNA (shRNA) or a scrambled shRNA (negative control). Silencing of *SLC25A12* or *SLC25A13* significantly reduced their protein levels (Fig. EV2A,B). However, the knockdown of *SLC25A12* (SLC25A12^KD^) did not result in a reduction in EC proliferation, but only *SLC25A13* knockdown (SLC25A13^KD^) significantly reduced EC proliferation as measured by 6-^3^H thymidine incorporation (reduction in mean incorporation - SLC25A13^KD^: 40%) (Figs. 2A and EV2C). In addition, qPCR and Western blot analysis confirmed that silencing *SLC25A12* does not lead to a compensatory upregulation of SLC25A13, suggesting the two transporters function independently of each other (Fig. EV2D,E). Therefore, we further focused on SLC25A13 and no longer on SLC25A12. A significant reduction in EC proliferation of SLC25A13^KD^ ECs was confirmed by an EdU incorporation assay (Fig. 2A). Further, SLC25A13^KD^ EC growth was reduced as compared to CTRL ECs, when monitoring cell confluence over time (72 h) (Fig. 2B). These observations identify SLC25A13 as a key mediator of the essential mitochondrial aspartate–glutamate antiport function predicted by EC-GEM and confirm its critical role in EC proliferation. To confirm the importance of SLC25A13 in EC proliferation, apoptosis, necrosis, necroptosis, and cell cycle were also evaluated, but only a slight increase in apoptosis was observed after silencing *SLC25A13* in ECs (Fig. EV2F–J). Silencing of *SLC25A13* led to an S-phase arrest, indicating alterations in the cell cycle. Biomass impairment might be associated with nucleotide deprivation, which can cause cells to arrest in the S phase, accompanied by a decrease in the G2/M phase (Fig. EV2I,J). Although *SLC25A13* silencing altered cell-cycle progression, the fractional ^13^C-labeling patterns and metabolite rescue phenotypes reported below are not inconsistent with direct metabolic effects, as fractional enrichment and functional rescue are generally less sensitive than absolute pool sizes to cell-cycle compositional shifts.

SLC25A13^KD^ also reduced the migratory capacity of mitomycin C-treated mitotically inactivated ECs (mean reduction in wound closure capacity - SLC25A13^KD^: 48%) in a scratch wound assay (Fig. 2C). In accordance with reduced migration, one of the two main cellular processes during sprouting angiogenesis, SLC25A13^KD^ reduced the number and length of vessel sprouts in a mitomycin C-treated mitotically inactivated EC spheroid sprouting assay (Figs. 2D and EV2K).

We then tested if Slc25a13 silencing inhibits neovascularization in vivo in laser-injured choroids in mice, an in vivo model of age-related macular degeneration (AMD) characterized by proliferative pathological angiogenesis (Schoors et al, 2014). Intravitreal injection of a siRNA against murine* Slc25a13* (siSlc25a13) decreased the transcript levels of the gene by 54% in ECs isolated from the choroid lesions as compared to ECs from control siRNA (siNC1) treated mice (Fig. EV2L). This reduction in *Slc25a13* expression also inhibited the laser-induced neovascularization (Fig. 2E; decrease in blood vessel area by 54% compared to siNC1). However, since the silencing in this model is not EC-specific, effects on other cell types could also contribute to this in vivo vascular phenotype. Thus, silencing of *SLC25A13* leads to defective EC proliferation, migration, and sprouting in vitro and in vivo.

### Mitochondrial glutamate–aspartate shuttling links to metabolic functions in ECs

**Figure 3:**
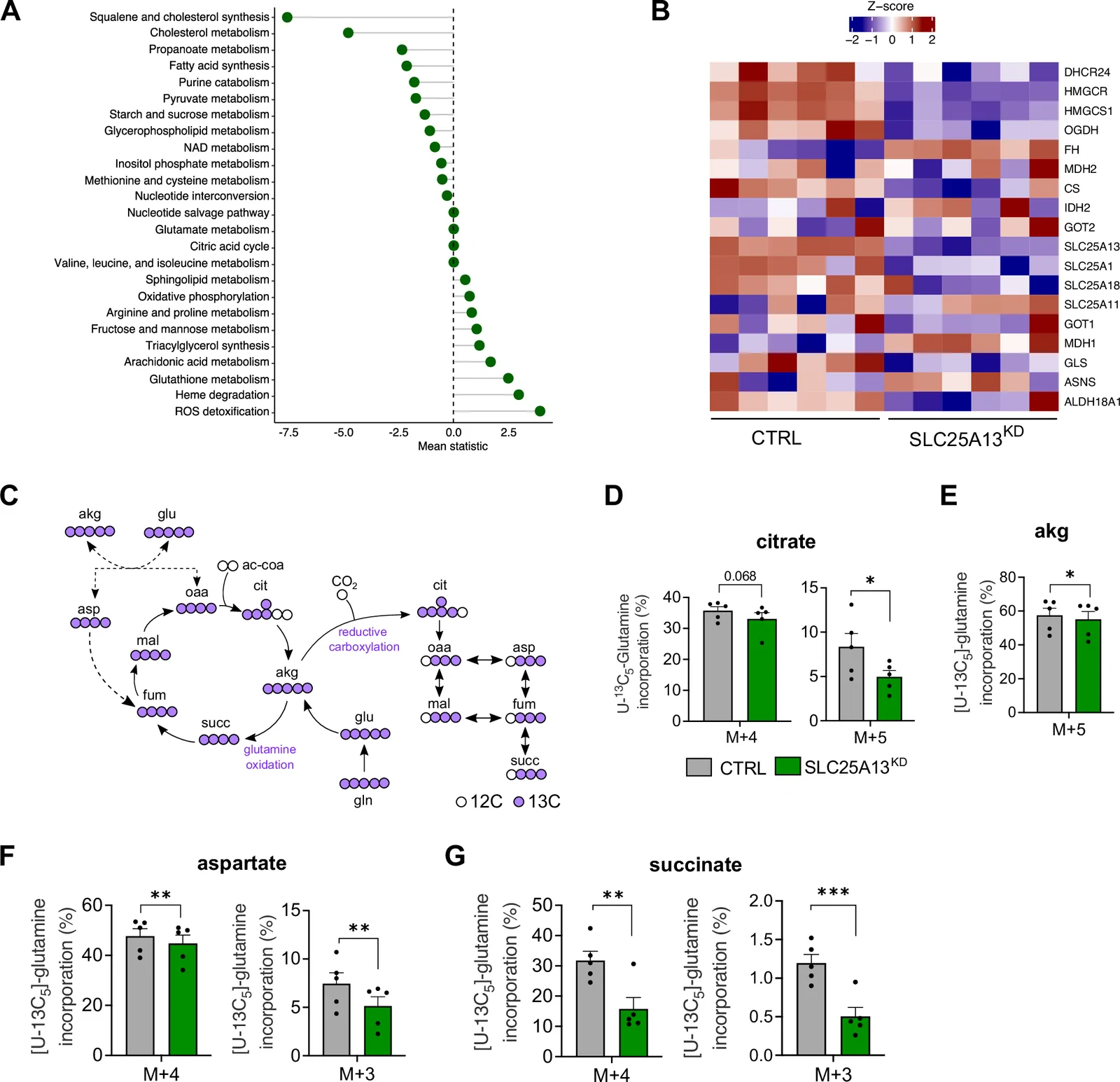
*SLC25A13* silencing affects TCA cycle metabolic function. (**A**) Self-contained enrichment analysis across 25 metabolic gene sets. Lollipop visualization of the mean t-statistic of each gene set. The length of the lollipops indicates the deviation from a zero mean (deviation from the zero mean to the left indicates a downregulation; deviation from the zero mean to the right indicates an upregulation). All significantly enriched gene sets (adj. *P* value < 0.05) are displayed by a line deviating from zero mean. (**B**) Heatmap showing the variation in expression of genes linked to the malate–aspartate shuttle from bulk RNA-sequencing of CTRL and SLC25A13^KD^ ECs. (**C**) Scheme demonstrating the utilization of uniformly labeled ^13^C glutamine ([U-^13^C]) glutamine within oxidative and reductive pathways of the TCA cycle. The ^13^C labeled carbons are indicated by purple-colored labels, whereas the ^12^C unlabeled carbons are represented by colorless circles. D-G. Fractional contribution of [U-^13^C]-glutamine carbons in the indicated isotopomers of citrate (*n* = 5) (**D**), a-ketoglutarate (akg) (*n* = 5) (**E**), aspartate (*n* = 5) (**F**), and succinate (*n* = 5) (**G**) in CTRL and SLC25A13^KD^ ECs. akg α-ketoglutarate, asn asparagine, asp aspartate, cit citrate, ctp cytidine triphosphate, dhor dihydroorotate, gln glutamine, glu glutamate, mal malate, mev mevalonate, nh4 ammonium ion, oaa oxaloacetate, pro proline, succ succinate, succoa succinyl coA, ump uridine monophosphate, utp uridine triphosphate. Data are means ± SEM; *P < 0.05, **P < 0.01, ***P < 0.001 by two-tailed paired *t* test (**D**–**G**). Exact *P* values: (**D**) M + 5, *P* = 0.0158; (**E**) *P* = 0.0181; (**F**) M + 4, *P* = 0.0055; M + 3, *P* = 0.0040; (**G**) M + 4, *P* = 0.0012; M + 3, *P* = 0.0008. Source data are available online for this figure.

Since SLC25A13 is a mitochondrial transporter and part of the malate–aspartate shuttle (Palmieri et al, 2001), we further explored its role in metabolic EC functions. We performed high-throughput bulk RNA-sequencing and analyzed the differences between the gene expression profiles of CTRL and SLC25A13^KD^ ECs (HUVECs) (Dataset EV2A,B). A self-contained gene-set enrichment analysis was performed on the bulk RNA-sequencing data from CTRL and SLC25A13^KD^ ECs (Dataset EV2B). Gene sets involved in cholesterol metabolism, purine catabolism, fatty acid synthesis, pyruvate metabolism, propanoate metabolism, and glycerophospholipid metabolism were downregulated (mean statistic <0) upon *SLC25A13* silencing in ECs (Fig. 3A; Dataset EV2B); because *SLC25A13* silencing also altered cell-cycle distribution (Fig. EV2I,J), a portion of these bulk transcriptional changes may reflect compositional shifts in addition to direct SLC25A13-dependent regulation. More specifically, the expression of TCA cycle genes (citrate synthase (CS), alpha-ketoglutarate dehydrogenase or oxoglutarate dehydrogenase (OGDH), the aspartate–glutamate shuttle (SLC25A13), glutamate transporter (SLC25A18), citrate–malate shuttle (SLC25A1), glutamine oxidation (GLS), proline synthesis (ALDH18A1), cholesterol synthesis (HMGCR, HMGCS1, DHCR24) was downregulated upon *SLC25A13* silencing (Fig. 3B).

### SLC25A13 regulates citric acid cycle function

**Figure EV3:**
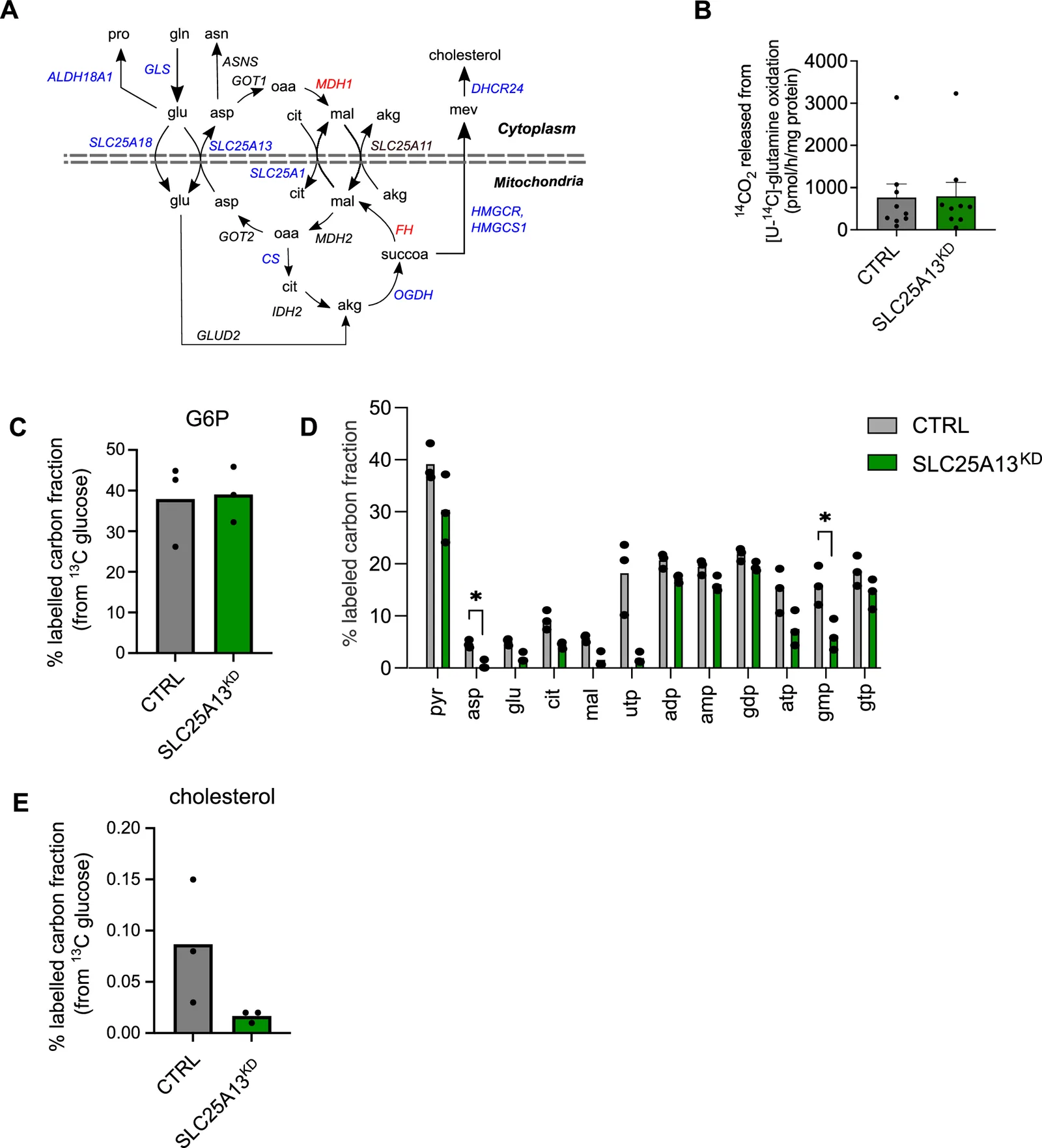
*SLC25A13* knockdown alters malate-aspartate shuttle activity and cellular carbon metabolism. (**A**) Scheme demonstrating the metabolic reactions associated with the malate–aspartate shuttle. Genes associated with these reactions are displayed on the reaction arrows. Blue colored gene names represent genes that are downregulated, and red-colored gene names represent genes that are upregulated upon SLC25A13^KD^. (**B**) Glutamine oxidation: Amount of ^14^CO_2_ released as a consequence of glutamine oxidation in CTRL and SLC25A13^KD^ ECs (*n* = 9). (**C**) Carbon fraction of glucose-6-phosphate (G6P) labeled from U-^13^C glucose (*n* = 3). (**D**) Barplots representing the total fraction of [U-^13^C]-glucose carbon in pyruvate (pyr), amino acids (asp, glu), TCA metabolites (cit, mal), nucleotides (ump, adp, amp, gdp, atp, gmp, gtp), and cholesterol precursor (mev) (*n* = 3). (**E**) Carbon fraction of cholesterol labeled from U-^13^C glucose (*n* = 3). adp adenosine diphosphate, atp adenosine triphosphate, amp adenosine monophosphate, asp aspartate, cit citrate, gdp guanosine diphosphate, glu glutamate, gmp guanosine monophosphate, mal malate, pyr pyruvate, utp uridine triphosphate. Data are means ± SEM; not significant, *P < 0.05 by a two-tailed paired *t* test (**D**). Exact *P* values: (**D**) asp, *P* = 0.044900; gmp, *P* = 0.030079.

Given SLC25A13’s involvement in mitochondrial glutamate import and the finding that the expression of GLS, the gene/protein associated with glutamine oxidation to glutamate, was downregulated in SLC25A13^KD^ ECs (Figs. 3B and EV3A), we hypothesized that SLC25A13 might be essential for glutamine oxidation and utilization into the TCA cycle (Fig. EV3A). To test this hypothesis, we used [U-^13^C] glutamine isotope tracing (Fig. 3C). When glutamine (gln) is oxidized in the forward direction (forward cycling or glutamine oxidation), citrate (cit) derived from [U-^13^C] glutamine will be labeled on four carbons (M + 4) (since one carbon will be released in the TCA cycle as CO_2_ when akg is converted into succinyl-CoA), whereas reductive carboxylation of glutamine labels five carbons (M + 5) of citrate (Fig. 3C). Notably, the M + 5 fraction of citrate, formed due to reductive carboxylation of glutamine (via M + 5 α-ketoglutarate (akg)) was significantly reduced by 40% in SLC25A13^KD^ ECs, whereas the M + 4 fraction of citrate showed only a modest insignificant decrease (Fig. 3D). Of note, the M + 5 fraction of akg, which represents the fully labeled fraction of akg from [U-^13^C] glutamine, shows only a modest decrease between CTRL and SLC25A13^KD^ ECs (Fig. 3E). We thus verified whether glutamine oxidation to CO_2_ is affected upon SLC25A13^KD^.

As expected, ^14^CO_2_ production (generated because of the α-ketoglutarate dehydrogenase and isocitrate dehydrogenase reactions in the TCA cycle) from [U-^14^C] glutamine remained unchanged between CTRL and SLC25A13^KD^ ECs (Fig. EV3B). Aspartate, which is an important precursor of amino acid and nucleotide synthesis, is derived from intermediates of the TCA cycle. No change in the forward cycling of glutamine oxidation possibly suggests the recycling of aspartate (derived from oxaloacetate) back into the TCA cycle via fumarate towards oxaloacetate (Fig. 3C). The reversible transaminase reactions oaa <--> asp couples with reversible asp <--> fum towards oaa again (Fig. 3C). Consequently, the fraction of M + 4 aspartate carbons generated because of forward cycling was reduced by only 7% in SLC25A13^KD^ ECs as compared to CTRL ECs (Fig. 3F), whereas a 30% reduction in the M + 3 aspartate carbon fraction, generated as a result of reductive carboxylation, was observed (Fig. 3F). Forward cycling of aspartate to generate oxaloacetate also explains the modest reduction observed in M + 4 cit and M + 5 akg (Fig. 3D). Since multiple amino acid transaminases (*e.g*. branched chain amino acid, kynurenine, alanine transaminase, tyrosine and tryptophan transaminases) can potentially utilize akg (in this case M + 5 akg) as a cofactor, akg is less available for succinate formation through TCA. Thus, a significant decrease in M + 4 succinate is observed in SLC25A13^KD^ ECs as compared to CTRL ECs (Fig. 3G). Owing to the observed decrease in overall glutamine utilization in the citric acid cycle, M + 3 and M + 4 succinate fractions were also significantly reduced in SLC25A13^KD^ ECs as compared to CTRL ECs (Fig. 3G), clearly demonstrating the defects in glutamine utilization in the citric acid cycle (Fig. 3C). Overall, by comparing the relative amount of labeled carbon fractions of metabolites generated from oxidative or reductive glutamine catabolism, the reductive carbon fractions in citrate and aspartate were found to be more compromised than the oxidative fractions. On the other hand, both M + 4 and M + 3 fractions of labeled succinate were significantly reduced.

### SLC25A13 regulates biomass synthesis

**Figure 4:**
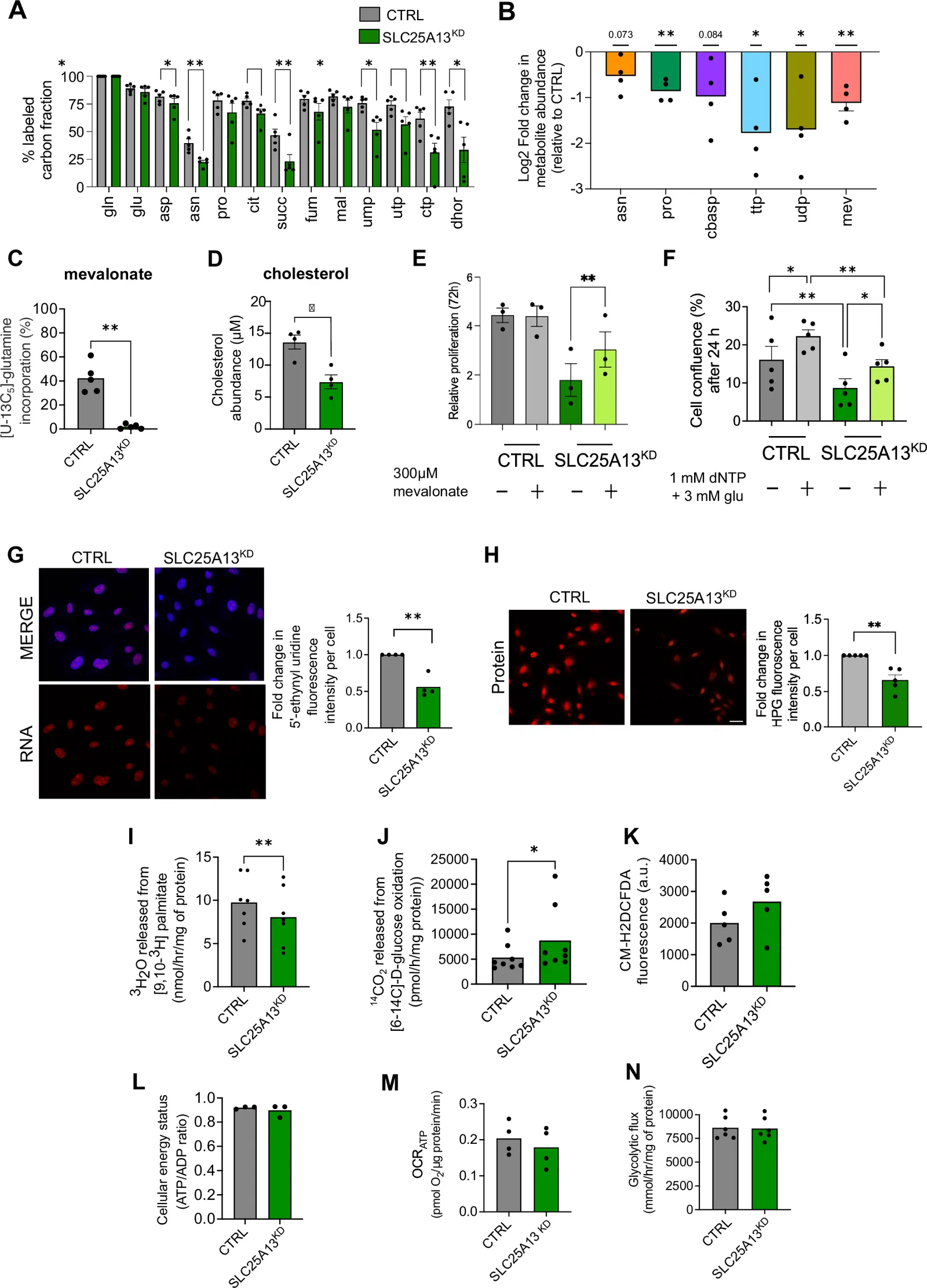
*SLC25A13* silencing affects biomass synthesis. (**A**) Barplots representing the total fraction of [U-^13^C]-glutamine carbon in amino acids (asn, asp, pro), nucleotide precursors (dhor), nucleotides (ump, utp, ctp), and cholesterol precursor (mev) (*n* = 5). (**B**) Log-fold change in the abundance of amino acids, nucleotide precursors, and nucleotides in SLC25A13^KD^ ECs relative to the CTRL ECs (*n* = 4). (**C**) Total fraction of [U-^13^C]-glutamine carbon in mevalonate in CTRL and SLC25A13^KD^ ECs (*n* = 5). (**D**) Difference in cholesterol abundance between CTRL and SLC25A13^KD^ ECs (*n* = 4). (**E**) Rescue with excess Mevalonate: Barplots demonstrating the comparison between CTRL and SLC25A13^KD^ ECs grown in EGM2 without and with 300 µM Mevalonate (*n* = 3). (**F**) Proliferation (measured by cell confluence) of CTRL and SLC25A13^KD^ ECs without and with supplementation of Glutamate + dNTP to the medium (*n* = 5). Scale bar: 50 µm. (**G**) Effect of *SLC25A13* silencing on RNA synthesis. Left: Representative images of 5’-ethynyl uridine fluorescence (red) in CTRL and SLC25A13^KD^ ECs. Nuclei are counterstained with DAPI (blue). Right: Fold change in 5’-ethynyl uridine fluorescence in SLC25A13^KD^ ECs (*n* = 4). Scale bar: 50 µm. (**H**) Effect of *SLC25A13* silencing on protein synthesis. Left: Representative images of homopropargylglycine (HPG) fluorescence (red) in CTRL and SLC25A13^KD^ ECs. Right: Fold change in HPG fluorescence in SLC25A13^KD^ ECs (*n* = 5). (**I**) Effect of *SLC25A13* silencing on fatty acid oxidation: [9-10-^3^H] palmitate oxidation measured by released ^3^H_2_O levels in CTRL and SLC25A13^KD^ ECs (*n* = 7). (**J**) Effect of SLC25A13 silencing on glucose oxidation: Amount of ^14^CO_2_ released because of [6-^14^C] D-glucose oxidation in CTRL and SLC25A13^KD^ ECs (*n* = 8). (**K**) Change in cytosolic oxidative stress: Measurement of CM-H2DCFDA fluorescence. (**L**) Effect of *SLC25A13* silencing on cellular energy status, measured as (ATP + 1/2 ADP)/(ATP + ADP + AMP) (*n* = 3). (**M**) Oxygen consumption rate coupled to ATP production (OCR_ATP_) in CTRL and SLC25A13^KD^ ECs (*n* = 4). (**N**) Glycolytic flux in CTRL and SLC25A13^KD^ ECs (*n* = 6). asn asparagine, asp aspartate, cbasp N-carbamoyl-L-aspartate, cit citrate, ctp cytidine triphosphate, dhor dihydroorotate, fum fumarate, gln glutamine, glu glutamate, mal malate, mev mevalonate, pro proline, succ succinate, ttp thymidine triphosphate, udp uridine diphosphate, ump uridine monophosphate, utp uridine triphosphate. Data are means ± SEM; not significant, *P < 0.05, **P < 0.01 by one-sample *t* test and Wilcoxon test (**B**, **G**); two-tailed paired *t* test (**A**, **C**, **D**, **E**, **H**–**N**); one-way ANOVA (**F**). Exact *P* values: (**A**) asp, *P* = 0.0473; asn, *P* = 0.0050; cit, *P* = 0.0185; succ, *P* = 0.0020; ump, *P* = 0.0105; utp, *P* = 0.0123; ctp, *P* = 0.0021; dhor, *P* = 0.0156 (**B**) pro, *P* = 0.0057; ttp, *P* = 0.028; udp, *P* = 0.0332; mev, *P* = 0.0081; (**C**) *P* = 0.0022; (**D**) *P* = 0.0330; (**E**) *P* = 0.0023; (**F**) CTRL (−) vs CTRL (+), *P* = 0.0241; CTRL (−) vs SLC25A13^KD^ (−), *P* = 0.0091; CTRL (+) vs SLC25A13^KD^ (+) *P* = 0.0062; SLC25A13^KD^(−) vs SLC25A13^KD^(+), *P* = 0.0354, (**G**) *P* = 0.0095; (**H**) *P* = 0.0096; (**I**) *P* = 0.0054; (**J**) *P* = 0.0228. Source data are available online for this figure.

Given SLC25A13’s role in EC proliferation and its involvement in biomass-related processes (bulk RNA-sequencing), we further explored its role in biomass synthesis, since this can provide the building blocks required for blood vessel formation. Glutamine-derived aspartate can be used to form asparagine (asn), pyrimidine precursor dihydroorotate (dhor), and nucleotides (ump, utp, ctp) (Sullivan et al, 2015; Yoo et al, 2020; Zhang et al, 2020). The glutamine carbon-labeled fractions of all these metabolites were considerably decreased in SLC25A13^KD^ ECs (Fig. 4A), indicating a role for SLC25A13 in modulating glutamine utilization for asparagine and nucleotide synthesis, important metabolites for EC proliferation (Huang et al, 2017). Similarly, labeled fractions of proline formed from glutamine-derived glutamate tended to be decreased in SLC25A13^KD^ ECs (Fig. 4A). These results indicate a clear link of SLC25A13^KD^ to glutamine utilization and its implications for the synthesis of glutamine-derived biomass metabolites. To test whether SLC25A13 is specific for glutamine, we also performed [U-^13^C] glucose isotope labeling. We observed no change in either labeled glycolytic metabolites (glucose-6-phosphate (g6p), pyruvate (pyr)), TCA metabolites (citrate, malate), or cholesterol (Fig. EV3C,E). We only observed significant alteration in the labeled nucleotide guanosine-monophosphate (gmp) and the amino acid aspartate (asp) (Fig. EV3D). Although we observed changes specifically in glutamine utilization, we aimed to investigate whether perturbations in SLC25A13 function also generate a global effect on overall biomass metabolites in proliferating ECs. Therefore, we performed metabolomics profiling on metabolites extracted from SLC25A13^KD^ and CTRL ECs using Liquid chromatography-mass spectrometry (LC–MS). Relative asn abundances showed a decreasing trend in SLC25A13^KD^ ECs because of impaired reductive carboxylation (Fig. 4B), whereas the relative abundances of mevalonate (mev), nucleotides (thymidine triphosphate (ttp), uridine diphosphate (udp)), N-carbamoyl-L-aspartate (cbasp), and proline were significantly decreased in SLC25A13^KD^ ECs (Fig. 4B). These findings point to a role of SLC25A13 in the general regulation of cholesterol synthesis, amino acid, and nucleotide synthesis, which are essential components of biomass.

**Figure EV4:**
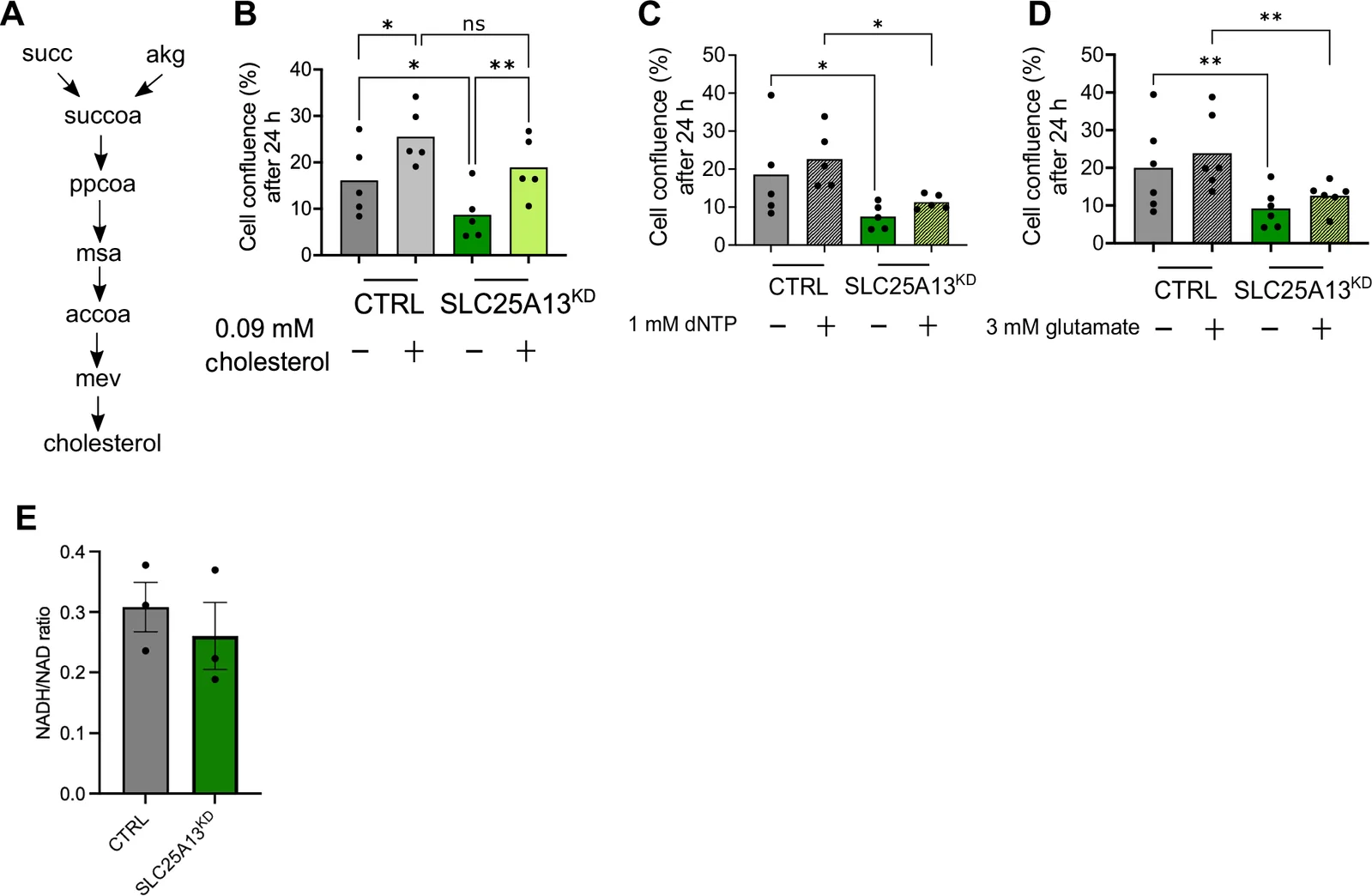
SLC25A13 knockdown limits endothelial cell proliferation through impaired cholesterol, nucleotide, and glutamate metabolism. (**A**) Pathway showing the catabolism of TCA metabolites to form mevalonate and hence, cholesterol. (**B**) Rescue with excess cholesterol: Barplots demonstrating the comparison between CTRL and SLC25A13^KD^ ECs grown in EGM2 + 0.09 mM methyl-β-cyclodextrin cholesterol (*n* = 5). (**C**) Proliferation (measured via percentage of cell confluency) of CTRL and SLC25A13^KD^ ECs, cultured in the presence or absence of 1 mM dNTP (*n* = 5). (**D**) Proliferation (measured via percentage confluence) of CTRL and SLC25A13^KD^ ECs, cultured with or without excess glutamate (3 mM) (*n* = 6). (**E**) Cellular NADH/NAD^+^ ratio in CTRL and SLC25A13^KD^ ECs (*n* = 3). accoa acetyl-coA, akg α-ketoglutarate, mev mevalonate, msa malonate semialdehyde, ppcoa propionyl-coA, succ succinate, succoa succinyl-coA. Data are means ± SEM; ns, not significant, *P < 0.05, **P < 0.01 by one-way ANOVA (**B**–**D**). Exact *P* values: (**B**) CTRL (−) vs CTRL (+), *P* = 0.0143; CTRL (−) vs SLC25A13^KD^ (−), *P* = 0.0435; SLC25A13^KD^ (−) vs SLC25A13^KD^ (+), *P* = 0.090; (**C**) CTRL (−) vs SLC25A13^KD^ (−), *P* = 0.0121; CTRL (+) vs SLC25A13^KD^ (+), *P* = 0.0104; (**D**) CTRL (−) vs SLC25A13^KD^ (−), *P* = 0.0075; CTRL (+) vs SLC25A13^KD^ (+), *P* = 0.0056.

Furthermore, glutamine-derived akg and succinate might also be routed for the production of cholesterol via succinyl-coA production (Fig. EV4A). To verify whether SLC25A13^KD^ affects cholesterol synthesis, we also measured the total glutamine-labeled fraction of mevalonate, a precursor of cholesterol synthesis. Indeed, we observed a significant reduction in glutamine-derived, labeled mevalonate levels upon SLC25A13^KD^ (Fig. 4C)_._ This again correlates with the significant reduction observed in glutamine-labeled succinate fractions upon SLC25A13^KD^ (Fig. 3G). In addition, a significant reduction in the relative abundance of mevalonate and absolute abundance of cholesterol was also observed in SLC25A13^KD^ ECs (Fig. 4B,D).

To verify whether supplementation of the affected metabolites could rescue the defect in biomass synthesis and thus EC proliferation (measured by cell confluence, since [^3^H]-thymidine incorporation could not be used as a measure for proliferation because the supplemented dNTPs would dilute the [^3^H]-thymidine pool) in SLC25A13^KD^ ECs, excess glutamate, dNTPs, mevalonate or cholesterol, were added to the culture medium. To explore whether we could restore the reduction in mevalonate in SLC25A13^KD^ ECs, we treated HUVECs with excess mevalonate (300 µM). Notably, we observed a partial restoration of cell growth (proliferation rate) after 72 h (Fig. 4E). Similarly, excess cholesterol (0.09 mM) was able to rescue cell confluence levels at least to similar levels as the CTRL cells after 24 h (Fig. EV4B). Indeed, proliferation of SLC25A13^KD^ ECs was nearly completely restored upon exogenous provision of dNTPs and glutamic acid (Fig. 4F). Not surprisingly, dNTPs or glutamate individually did not rescue cell confluence (Fig. EV4C,D).

Further, nascent RNA synthesis measured by 5’-ethynyl uridine (5-EU) fluorescence intensity was reduced in SLC25A13^KD^ ECs as compared to CTRL ECs (Fig. 4G), in agreement with the pyrimidine (udp and ttp) deficit observed from the metabolomics measurements (Fig. 4B). Similarly, global protein synthesis as measured by the incorporation of the amino acid analog homopropargylglycine (HPG) was decreased in SLC25A13^KD^ ECs as compared to CTRL ECs (Fig. 4H). The reduction in thymidine, 5-EU, and HPG incorporation upon SLC25A13^KD^ further supports the EC-GEM-predicted essential role of the mitochondrial aspartate–glutamate antiport function in biomass synthesis. A moderate decrease in fatty acid oxidation (FAO) (as measured by the conversion of [9-10-^3^H]-palmitate to ^3^H_2_O) was also observed in SLC25A13^KD^ ECs (Fig. 4I). This suggests a link between SLC25A13 and FAO, previously reported to be essential for biomass synthesis (nucleotides) during EC proliferation (Patella et al, 2015; Schoors et al, 2015). Furthermore, the glucose oxidation rate nearly doubled in SLC25A13^KD^ ECs (Fig. 4J), possibly suggesting a compensatory response by the cells to the impairment of the reductive carboxylation; however, apparently failing to restore the defects observed in EC proliferation, migration, and sprouting (Fig. 2A–D).

Since SLC25A13, being part of the malate–aspartate shuttle, is canonically known to maintain cellular redox homeostasis by transporting NADH reducing equivalents from the cytosol into mitochondria and by regulating the influx/efflux of protons via exchange of aspartate/glutamate (Palmieri, 2004), we verified the redox status. The cellular NADH:NAD ratio in SLC25A13^KD^ ECs showed no significant difference from that of CTRL ECs (Fig. EV4E). In line with this observation, CM-H2DCFDA fluorescence, an indicator of cellular reactive oxygen species (ROS) did not change in the SLC25A13^KD^ ECs (Fig. 4K). These findings show that SLC25A13’s primary role in ECs is not related to redox homeostasis, but rather to biosynthesis.

Finally, we tested whether the cellular energy status is affected in response to *SLC25A13* silencing. No change in the energy status ((ATP + 1/2 ADP)/(ATP + ADP + AMP)) was observed between CTRL and SLC25A13^KD^ ECs (Fig. 4L). Congruently, Oxygen Consumption Rate (OCR) associated with ATP production (OCR_ATP_) (Fig. 4M) and the glycolytic flux (the major ATP generator in proliferating ECs (De Bock et al, 2013)) remained unchanged (Fig. 4N). Thus, the SLC25A13 function is neither linked to glycolytic flux nor ATP synthesis. Overall, these data support a major role of SLC25A13 (and hence malate aspartate shuttle) in biomass synthesis (precursor of metabolites—amino acid, nucleotide, and cholesterol synthesis) rather than in the maintenance of redox balance.

## Discussion

In this study, we used our previously in-house developed EC-GEM to simulate the metabolic behavior of proliferating ECs. Additionally, we conducted flux and reaction knockout analyses to predict novel metabolic pathways for biomass synthesis in ECs. Reaction knockout analysis predicted the essential role of the mitochondrial glutamate–aspartate antiporter reaction in EC biomass synthesis. Predicted fluxes through the mitochondrial glutamate–aspartate antiporter reaction also indicated mitochondrial export of aspartate to be essential for biomass synthesis. Such a role for the glutamate–aspartate antiporter reaction in EC biomass synthesis has never been documented previously.

Only a few studies have investigated the role of mitochondrial glutamate–aspartate antiporters directly or indirectly in the context of cell proliferation (Gorgoglione et al, 2022; Petralla et al, 2019; Profilo et al, 2017). It has been documented that two different mitochondrial glutamate–aspartate antiporters (SLC25A12, SLC25A13) play essential metabolic roles in various proliferative cell types in terms of metabolic processes. SLC25A12 was shown to regulate cytosolic aspartate for nucleotide biosynthesis in HepG2 cells, glutamine-independent/glutamine-dependent cytosolic aspartate levels for nucleotide synthesis in cancer and immortalized cell lines, and N-acetyl aspartate production coupled with mitochondrial dysfunction in oligodendrocyte precursor cells (Alkan et al, 2018; Infantino et al, 2019; Lv et al, 2022; Miyo et al, 2016; Petralla et al, 2019; Rabinovich et al, 2020). Similarly, SLC25A13 was directly linked to glycolytic flux and energy production in proliferating cancer cells and fibroblasts (Rabinovich et al, 2020). Colorectal cancer cells exhibit an overexpression of SLC25A13, which is associated with increased amino acid metabolism upon glucose depletion (Miyo et al, 2016). Although these studies describe the functions of mitochondrial glutamate–aspartate antiporters in various proliferating cell types, nothing is known about their roles in ECs. We therefore experimentally addressed the question whether glutamate–aspartate antiporters serve analogous roles in ECs and explored its contribution to EC metabolic function.

Both SLC25A12 and SLC25A13 are detectable at the protein level in ECs. However, only the silencing of the antiporter *SLC25A13* caused a proliferation defect in ECs, suggesting that ECs in particular use this antiporter during proliferation. Silencing of *SLC25A13* impaired proliferation, migration, and spheroid vessel sprouting of ECs in vitro as well as in vivo pathological choroidal neovascularization. Based on these observations and EC-GEM predictions, we hypothesized and explored the role of antiporter SLC25A13 in modulating the synthesis of biomass precursors in ECs.

Glutamine metabolism in the TCA cycle was perturbed upon silencing the antiporter. Interestingly, reductive carboxylation of glutamine was reduced to a considerably larger extent than its oxidation, indicating a shift towards an intramitochondrial oxidative phenotype. Reductive carboxylation of glutamine in ECs has been previously documented (Huang et al, 2017; Kim et al, 2017) but was not studied upon perturbation of the malate–aspartate shuttle (SLC25A13).

**Figure 5:**
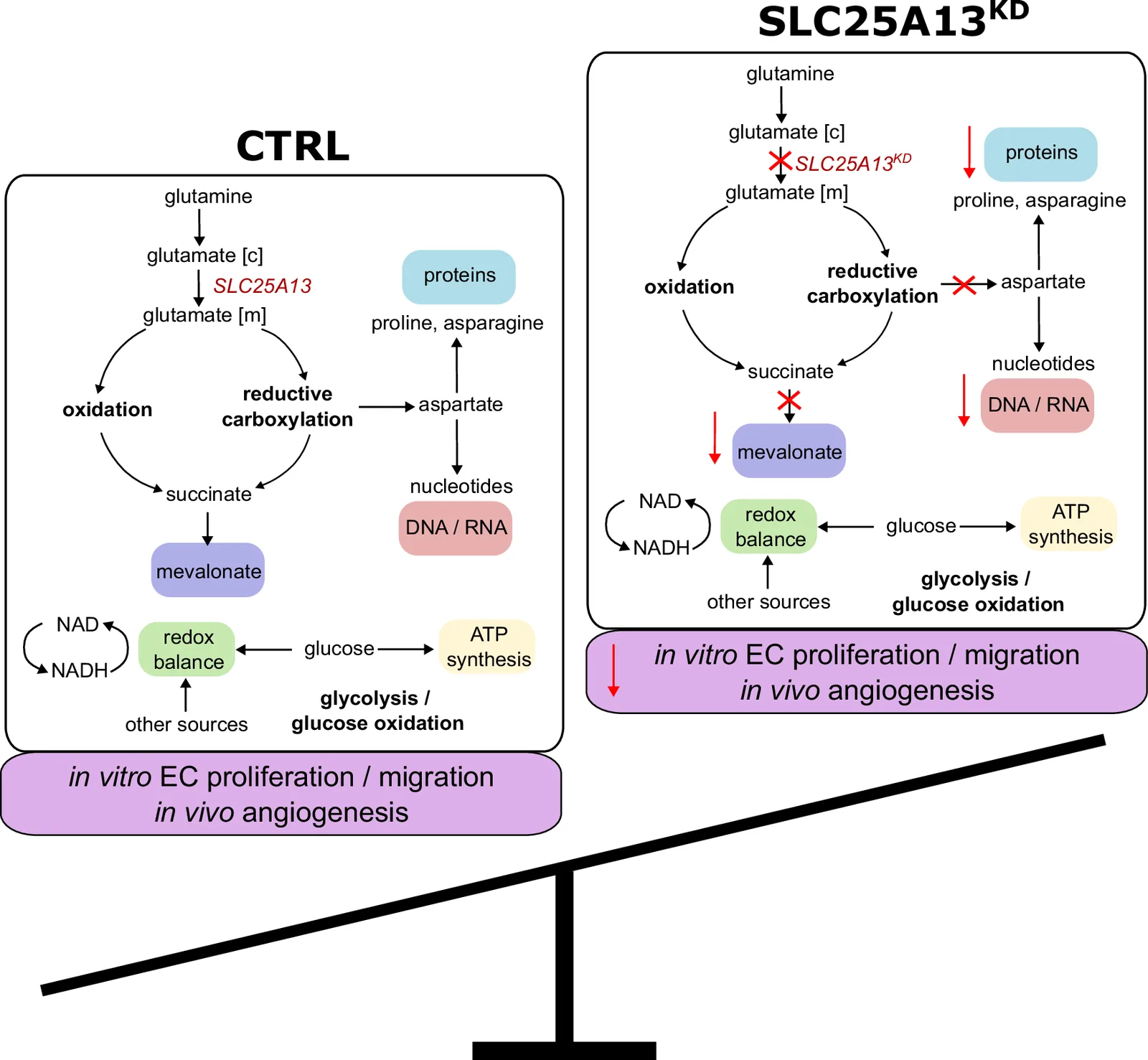
Working model for explaining the metabolic role of SLC25A13^KD^ in ECs. A scheme representing the metabolic role of SLC25A13^KD^ *vs*. control in ECs.

Combining observations from the different experiments, we propose a working model to explain the role(s) of SLC25A13 that link metabolic changes with EC angiogenic phenotypes (Fig. 5). Interestingly, tracing the fate of ^13^C glutamine clearly delineates a role of SLC25A13 in coupling glutamine reductive carboxylation with aspartate synthesis and coupling glutamine oxidation and reductive carboxylation together for overall succinate production. As explored in the EC-GEM, aspartate is a precursor for nucleotide synthesis, and succinate is linked to mevalonate/cholesterol synthesis. Glutamine-derived glutamate import into mitochondria is utilized for the synthesis of nucleotides, amino acids, and the cholesterol precursor mevalonate, and SLC25A13’s function is imperative for maintaining normal levels of these precursors.

Complementing these observations, functional assays further confirmed the role of SLC25A13 in DNA, RNA, protein synthesis, and in maintaining in vitro angiogenic EC phenotypes. Additionally, rescue experiments using mevalonate, a precursor of cholesterol biosynthesis, established SLC25A13’s role in modulating glutamine catabolism. Another study reported similar observations in other proliferating cell types where glutamine was shown to be metabolically essential for cholesterol synthesis and to transcriptionally control the mevalonate pathway (Garcia et al, 2024). We observe a similar dual role of glutamine utilization in mevalonate/cholesterol synthesis: as a metabolite contributing to mevalonate/cholesterol synthesis, and via transcriptional regulation of squalene and cholesterol synthesis gene sets, as observed via SLC25A13^KD^ experiments, suggesting both metabolic and non-metabolic modes of mevalonate/cholesterol regulation (Figs. 4 and 3A). Partial rescue of SLC25A13^KD^ EC proliferation suggests that impaired mevalonate synthesis contributes to the proliferation defect observed upon *SLC25A13* silencing. The distinct utilization of glutamine as an important substrate for cholesterol biosynthesis (mevalonate) and SLC25A13’s role in metabolically coupling glutamine catabolism with cholesterol biosynthesis is reported for the first time in any cell type. The contribution of SLC25A13 to biomass precursor generation is associated with impaired in vitro vessel sprouting. Slc25a13 gene silencing in murine models also affects blood vessel formation, though further EC-selective knockout studies are needed to demonstrate a direct role for ECs univocally.

In ECs, the specificity of SLC25A13 in biomass synthesis is intriguing, as it is also decoupled from glycolysis, glucose oxidation, and oxygen-coupled ATP production under basal conditions. This observation is quite opposite to proliferating cancer cells and fibroblasts, where inhibition of SLC25A13 leads to a decrease in glycolytic flux and ATP production under basal conditions (Rabinovich et al, 2020). Tracing the fate of ^13^C glucose in SLC25A13^KD^ EC metabolism clearly demonstrates that glucose catabolism via glycolysis occurs independent of SLC25A13 function, highlighting SLC25A13 to be dispensable for sustaining ATP synthesis. Like healthy ECs, SLC25A13^KD^ ECs probably retain their ability to maintain ATP synthesis through glycolysis. Isotope tracing experiments clearly demonstrate that glucose-derived TCA intermediates produced via oxidative metabolism remain largely unchanged upon SLC25A13 perturbation. In accordance, cellular NADH:NAD balance and ROS remain unaltered, pointing towards sustained redox balance achieved via glucose oxidation. Thus, maintenance of redox balance is also not the primary function of SLC25A13 in ECs (Fig. 5). Overall, these observations underscore a key biosynthetic role of glutamine in ECs, mediated by SLC25A13.

**Figure EV5:**
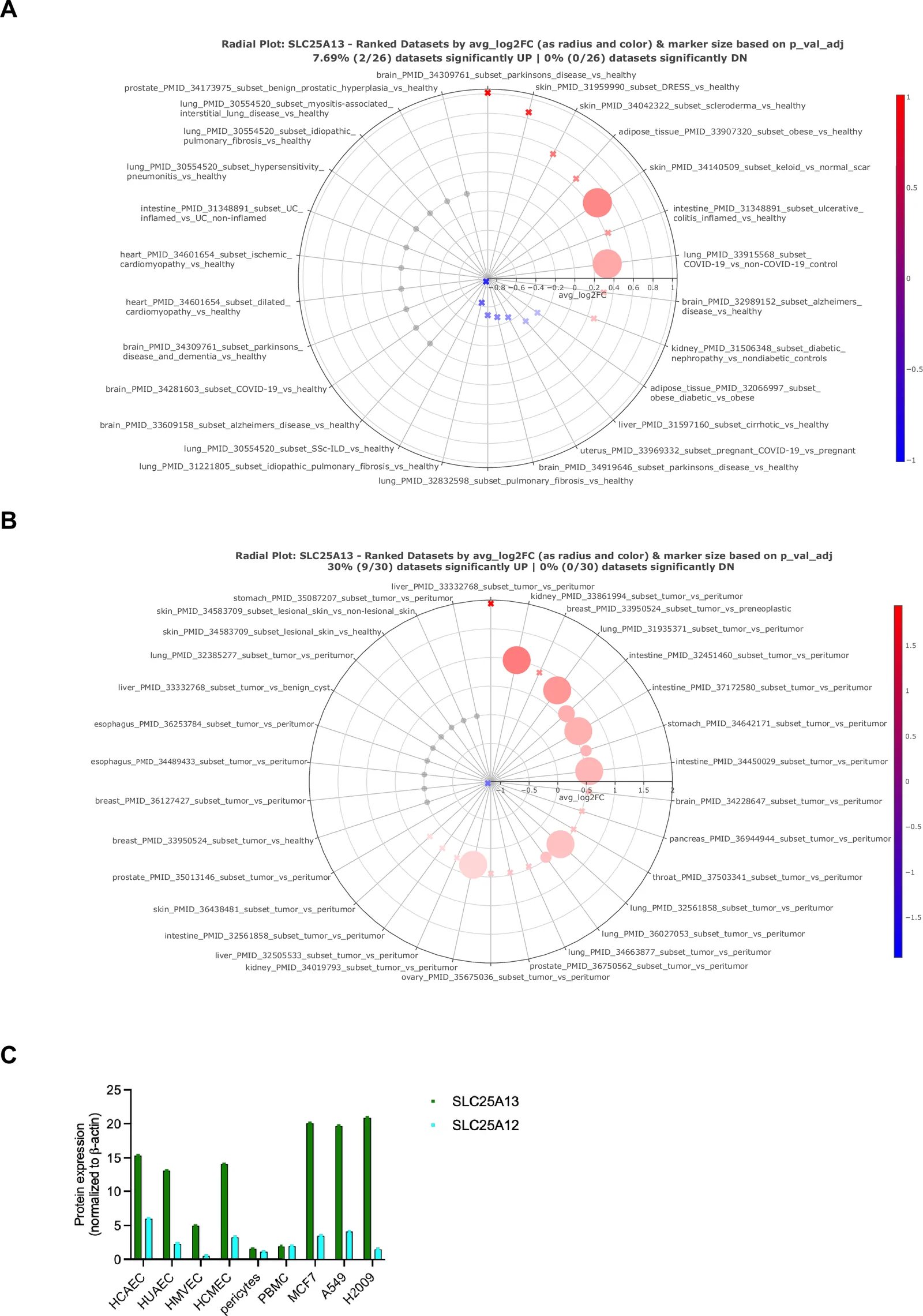
SLC25A13 expression across inflammatory and tumor datasets and vascular cell types. (**A**) Radial plot of *SLC25A13* expression in inflammatory datasets. (**B**) Radial plot of *SLC25A13* expression in tumor datasets. (**C**) SLC25A13 and SLC25A12 protein expression normalized to β-actin in vascular and non-vascular cells.

The involvement of SLC25A13 in regulating glutamine utilization and biomass precursor synthesis (DNA, RNA, and cholesterol biosynthetic pathways) makes it an attractive target for diseases characterized by aberrant angiogenesis (Fig. 5). SLC25A13 was upregulated in ECs across various inflammatory and tumor single-cell RNA sequencing datasets, further underscoring a potential clinical role in diseases (Fig. EV5A,B). Similarly, SLC25A13 exhibits heterogeneous expression across vascular and non-vascular cell types, including among endothelial subtypes, the functional significance of which remains to be determined. (Fig. EV5C). Overall, our novel findings may warrant further consideration of SLC25A13 as a possible candidate target for potential alternative anti-angiogenic therapies, likely more feasible if this metabolic candidate can be inhibited selectively in ECs in tumors. Overall, our findings warrant further exploration of the role of SLC25A13 in different diseases and conditions  characterised by pathological angiogenesis.

### Limitations and perspectives

Although EC-GEM is a valuable tool for the identification of essential metabolic genes, we acknowledge the limitations of our study. First, while transcript levels and metabolic modeling using EC-GEM offer valuable insights, it does not fully encompass the intricacies of metabolism, such as enzyme activities and metabolite levels. Similarly, EC-GEM FBA does not capture the non-linear, dose-dependent effects of partial gene modulation. Incorporating dynamics of target modulation would require complementary modeling approaches (e.g., kinetic modeling), which we view as an important future direction. Despite this limitation, the experimental validations in our previous article (Rohlenova et al, 2020) and current study show the ability of the EC-GEM to predict (with high accuracy) actual metabolic pathways and roles of metabolic enzymes in the context of EC proliferation. We anticipate that future additions to the EC-GEM (e.g., tailored to specific EC subtypes or to AAT-treated ECs) could further refine the discovery of promising therapeutic targets.

The EC-GEM and experiments in this study were conducted under standard culture conditions. We anticipate that the inclusion of physiologically relevant environmental perturbation data (hypoxia, high glucose, etc.) within the EC-GEM model, coupled with in vitro confirmations, could widen the study of metabolic adaptability during EC proliferation in future studies.

SLC25A13 knockdown alters cell-cycle distribution, with an accumulation of cells in S-phase. Our bulk RNA-sequencing, steady-state metabolomics, and synthesis assays were performed on asynchronous EC populations. Consequently, we cannot fully exclude that a portion of the observed transcriptional and metabolite-abundance changes reflects altered cell-cycle composition in addition to the direct metabolic consequences of SLC25A13 loss. Cell-cycle-matched or single-cell approaches will help further deconvolve these contributions.

We cannot exclude potential off-target effects of RNAi-mediated silencing on the observed phenotypes, which could be addressed via rescue experiments or orthogonal validation. Nevertheless, the phenotypes observed with *SLC25A13* knockdown were highly consistent across multiple independent assays.

In addition, formal compartment-resolved flux quantitation might be needed to quantify altered routing through reductive carboxylation. Compartment-targeted redox biosensors that can detect subcellular perturbations in NADH/NAD+ balance may be required under glycolytic stress.

Lastly, metabolic genes can also have non-canonical roles, which cannot be captured through a metabolic GEM model (e.g., pyruvate kinase M2 triggers innate immune signaling in ECs (Stone et al, 2018) or PCK2, which has a non-enzymatic role in proteostasis in ECs (de Zeeuw et al, 2024)). Exploring such functions, particularly for the prioritized SLC genes, could provide complementary biological insights and help refine the interpretation of EC metabolic regulation.

## Methods

**Table:** Reagents and tools table

Reagent/resource	Reference or source	Identifier or catalog number
**Experimental models**		
C57BL6/J mice	KU Leuven animal facility	
Human Umbilical Vein Endothelial Cells (HUVEC)	In-house isolated from umbilical cords from UZ Leuven Hospitals	
PBMCs	In-house isolated from donors from UZ Leuven Hospitals	
bEen3	ATCC	CRL-2299
Human pericytes from the placenta (hPC-PL)	PromoCell	C-12980
Human coronary artery endothelial cells (HCAEC)	Lonza Bioscience	CC-2585
Human Umbilical Artery Endothelial Cells (HUAEC)	In-house isolated from umbilical cords from UZ Leuven Hospitals	
Human lung microvascular endothelial cells (HMVEC-L)	Lonza Bioscience	CC-2527
Human Cardiac Microvascular Endothelial Cells (HCMEC)	PromoCell	C-12285
MCF7	ATCC	HTB-22
A549	ATCC	CCL-185
H2009	ATCC	CRL-5911
293T	ATCC	CRL-3216
**Antibodies**		
Rabbit anti-SLC25A12	Abcam	ab200201
Rabbit anti-SLC25A13	Abcam	ab167166
Rabbit anti-GAPDH	Cell Signaling	2118
Anti-rabbit Ig	Cell Signaling	7074
Anti-mouse Ig	Cell Signaling	7076
CD45 (PE-Cy7, dilution 1:500)	Thermo Fisher Scientific	25-0451-82
CD31 (AF488, dilution 1:100),	Thermo Fisher Scientific	11-0311-82
CD102/ICAM2 (APC, dilution 1:50)	Thermo Fisher Scientific	A15452
Fixable viability dye (VD, eFluorTM 450, dilution 1:1000	Thermo Fisher Scientific	65-0863-14
**Oligonucleotides and other sequence-based reagents**		
pLKO-shRNA2 vector (PLKO)	Clontech	No. PT4052-5
*SLC25A12* shRNA	Origene	NM_003705
*SLC25A13* shRNA	Origene	NM_014251
siRNA duplexes against (siSlc25a13; mm.Ri.Slc25a13.13.1) or control DsiRNA (siNC1)	IDT Integrated DNA Technologies	
*HPRT* Hs.PT.58.2145446	IDT Integrated DNA Technologies	NM_000194
*SLC25A12* Hs.PT.58.22913049	IDT Integrated DNA Technologies	NM_003705(2)
*SLC25A13* Hs.PT.58.25777150	IDT Integrated DNA Technologies	NM_001160210(3)
*Actb* Mm.PT.39a.22214843.g	IDT Integrated DNA Technologies	NM_007393
*Slc25a13* Mm.PT.58.9683680	IDT Integrated DNA Technologies	NM_001177572(2)
**Chemicals, enzymes, and other reagents**		
EGM2	PromoCell	C-22011
ECGM-2	PromoCell	C-39211
DMEM, high glucose	Thermo Fisher Scientific	11965092
M199 medium	Thermo Fisher Scientific	11150059
Knock-out ™ DMEM-medium	Thermo Fisher Scientific	10829018
EGM-2 Bullet kit	Lonza	CC-3202
RPMI 1640	GIBCO	11875093
Pericyte Growth Medium 2	PromoCell	C-28041
ECGM-MV Kit	PromoCell	C-22120
CM-H2DCFDA	Thermo Fisher Scientific	C6827
dNTPs	Thermo Fisher Scientific	10297018
Mevalonate	Sigma-Aldrich	90469-50MG
Cholesterol	Sigma-Aldrich	C4951-30MG
RIPA Lysis and Extraction Buffer	Invitrogen	89901
Amersham™ ECL Western Blotting Detection Reagents	GE Healthcare	RPN2106
Cytiva Amersham™ ECL™ Select	GE Healthcare	RPN2235
FITC–dextran (MW 2,000,000)	Sigma-Aldrich	FD2000S
Mitomycin C	Sigma-Aldrich	M0503-2MG
in vivo-jetPEI®	Polyplus-transfection SA	101000030
[^3^H]-thymidine	PerkinElmer	NET355L005MC
Antibiotic-antimycotic	Thermo Fisher Scientific	15240062
Bovine serum albumin (BSA Fraction V)	Sigma-Aldrich	10735086001
Collagenase type I	Thermo Fisher Scientific	17018029
Dispase	Thermo Fisher Scientific	17105-041
DNase I	Sigma-Aldrich	D4527-10KU
EDTA	Sigma-Aldrich	ED2P-500G
Endothelial cell growth factor supplements (ECGS/Heparin)	PromoCell	C-30120
Fetal bovine serum (FBS)	Thermo Fisher Scientific	16000044
Gelatin from bovine skin	Sigma-Aldrich	G9391
MEM NEAA	Thermo Fisher Scientific	11140035
Methylcellulose 4000 cP	Sigma-Aldrich	M0512
Paraformaldehyde Merck	Millipore	8.18715.1000
Penicillin/streptomycin	Thermo Fisher Scientific	15140122
Phosphate-buffered saline (DPBS)	Thermo Fisher Scientific	14190094
Phosphatase inhibitors (PhosSTOP)	Roche	04906837001
Protease inhibitors (cOmplete, EDTA-free Protease Inhibitor Cocktail)	Roche	11873580001
Sodium pyruvate	Thermo Fisher Scientific	11360070
TaqMan Fast Universal PCR Master Mix (2X)	Thermo Fisher Scientific	4364103
TCA (trichloroacetic acid)	Sigma-Aldrich	T6399
Nitrocellulose Pre-Cut Blotting Membranes, 0.45 µm pore size	Thermo Fisher Scientific	LC2001
NuPAGE 10% Bis-Tris Protein Gels, 1.5 mm, 10-well	Thermo Fisher Scientific	NP0315BOX
PUREPOINT®LASER	Alcon	
RNase A	Thermo Fisher Scientific	EN0531
[U-^14^C]glutamine	PerkinElmer	NEC451050UC
[6-^14^C]D-glucose	PerkinElmer	NEC045X050UC
[5-^3^H]D-glucose	PerkinElmer	NET531001MC
[9,10-^3^H]palmitic acid	PerkinElmer	NET043001MC
[U-^13^C]-L-glutamine	Cambridge Isotopes Laboratories	CLM-1166
Trypsin-EDTA (0.25%)	Thermo Fisher Scientific	25200056
L-carnitine	Sigma-Aldrich	C0283
Sodium palmitate	Sigma-Aldrich	P9767
**Software**		
MATLAB R2018a	The MathWorks, Inc.	
Cytoscape v.3.7.2	Cytoscape Consortium	
Fiji	Open-source	
NIH ImageJ	NIH	
FlowJo	FlowJo LLC	
R v.4.0.1.	Open-source	
Xcalibur	Thermo Fisher Scientific	
XF Reader 1.8.1.1.	Agilent Technologies	
QuantaSmart V4	PerkinElmer	
Leica MM AF morphometric analysis	Leica Microsystems	
GraphPad Prism v.8	GraphPad Software	
Inkscape v.0.9.1	Open-source	
**Other**		
Lipofectamine RNAiMAX transfection kit	Thermo Fisher Scientific	13778150
RNeasy Micro Kit	Qiagen	74004
PureLink RNA mini kit	Invitrogen	12183018 A
SuperScript III First Strand cDNA synthesis kit	Thermo Fisher Scientific	18080-051
iScript cDNA synthesis kit	Bio-Rad	1708891
TRIzol kit	Life Technologies	15596026
Illumina TruSeq Stranded mRNA Library prep kit	Illumina	20020595
DNA 1000 kit	Agilent Technologies	5067-1504
Annexin V/Propidium Iodide (PI) staining kit	Thermo Fisher Scientific	v13241
NAD/NADH Assay Kit	Sigma-Aldrich	MAK4681KT
Click-iT™ EdU Alexa Fluor™ 647 Flow Cytometry Assay Kit	Invitrogen	C10419
Pierce™ BCA Protein Assay Kit	Thermo Fisher Scientific	23225
Leica DMI6000 microscope	Leica Microsystems	
Zeiss LSM780 confocal microscope	Carl Zeiss	
Agilent 1260 HPLC	Agilent Technologies	
Seahorse XF24 device	Agilent Technologies	
Leica TCS SPE confocal microscope	Leica Microsystems	
BD FACSAria III	BD Biosciences	
LC–MS system: Dionex UltiMate 3000 LC system coupled to a Q Exactive Orbitrap mass spectrometer	Thermo Fisher Scientific	
GC–MS system: Trace 1610 gas chromatograph coupled to an Exploris 120 Orbitrap mass spectrometer	Thermo Fisher Scientific	

### The EC-GEM model

All the constraint-based flux analyses in this section are performed using COBRA toolbox v.3.0 (Heirendt et al, 2019) implemented in MATLAB R2018a.

#### Constraints used in the proliferation EC-GEM

The endothelial cell genome-scale metabolic model (EC-GEM), which we developed in our previous study (Rohlenova et al, 2020), was used for discovering novel catabolic routes. Maximization of an EC-specific biomass demand reaction was used as the objective function for flux-balance simulations. Similarly, the uptake rates of different metabolites were constrained with respect to the metabolite composition of the endothelial growth medium 2 (EGM2) (5.5 mM glucose, 10 mM glutamine, 1 mM pyruvate) typically used for culturing HUVECs (Rohlenova et al, 2020). As the fatty acid composition within EGM2 is unknown, we assumed an abundant availability of fatty acids for uptake (Rohlenova et al, 2020). As transcriptomics constraints alone might not fully capture the true metabolic behavior, two sets of functional constraints were additionally defined. These constraints are provided in Dataset EV1.

##### Fatty acid oxidation (FAO)-dependent constraints

We adopted the FAO-dependent constraints from our previous study (Rohlenova et al, 2020). The FAO-dependent constraints maximize the utilization of fatty acids for acetyl-coA production and glycolysis for ATP production (Rohlenova et al, 2020). These constraints help to mimic the mandatory use of fatty acids along with other anaplerotic substrates like glucose and amino acids, a phenomenon characteristic of ECs (Rohlenova et al, 2020).

##### Basal constraints

Pyruvate carboxylase flux was bounded in an arbitrary range (0–10 mmol gram dry weight (gDW)^−1^ h^−1^) such that its flux is always assumed to be lower than maximal mitochondrial fatty acid *β*-oxidation flux. In addition, the flux through the aspartate–glutamate antiporter was bound in an arbitrary range (−20 to 20 mmol gDW^−1^ h^−1^) so that the leakage of protons from mitochondria to cytosol via the malate–aspartate shuttle would be restricted (Rohlenova et al, 2020). These constraints help to maintain an EC-specific dependence of glycolysis on ATP dependence, compartmentalize glucose utilization for ATP production and catabolism through glycolytic side pathways for glycine synthesis, thereby lowering glucose oxidation within the mitochondria. We refer to these minimal EC-specific constraints as “basal” constraints.

#### Flux balance analysis

Flux balance analysis (FBA) (Orth et al, 2010), as implemented in the COBRA toolbox v.3.0 (Heirendt et al, 2019) on MATLAB R2018a, was used to estimate the steady-state reaction fluxes for the purpose of maximizing biomass synthesis in the EC-GEM, subject to the FAO-dependent constraints. The CycleFreeFlux algorithm implemented in the COBRA toolbox was used to remove the thermodynamically infeasible loops from the flux distribution obtained following maximization (Desouki et al, 2015). The predicted fluxes were employed to manually trace the fate of different environmental metabolites.

#### Reaction knockout analysis (reaction essentiality)

The Fast-SL algorithm was used for efficient genome-scale identification of essential reactions (Pratapa et al, 2015). A reaction was deemed essential if the knockout of a reaction (constraining the upper and lower flux bounds of the reaction to zero flux) led to at least a 1% reduction in optimal biomass flux; else, they were deemed to be non-essential. Genes associated with these reactions were also considered to be essential. Genes that were explicitly tested or studied for their essential metabolic role in the synthesis of biomass in proliferating ECs from diverse experimental techniques involving in vitro or in vivo targeted gene deletion, inhibition of reactions by small molecule analogs, and/or gene knockdowns were extensively curated from literature (Dataset EV1). Thereby, the experimentally determined classification of 34 genes as metabolically essential or non-essential for biomass synthesis was used for comparison with the essentiality predictions obtained from EC-GEM simulations.

#### Flux split analysis

The estimated (thermodynamically feasible) flux distribution in the FAOmax situation was used to calculate flux splits across reactions, thereby measuring the relative contributions of reactions (fluxes) that produce or consume a given metabolite (Aurich et al, 2016). Here, we were interested to know the fractional flux contributions of reactions that consume glutamate, the most central metabolite within the biomass metabolite core.

#### Flux sampling and correlation network analysis

To simulate alternative flux distributions that can produce maximal biomass, coordinate hit and-run with rounding (CHRR) sampling (Haraldsdottir et al, 2017) was used to sample 10,000 random flux distributions, following the application of the basal constraints in the EC-GEM. For this purpose, the maximal constraint on biomass was changed to 0.025 mmol.gDW^−1 h−1^ (Rohlenova et al, 2020). The sampling was performed using the “gurobi” solver v.9.1.2. Pearson correlation coefficients were computed for every pair of reactions across the sample flux distributions to generate a 3897 × 3897 correlation matrix. This correlation matrix was used to generate an adjacency matrix where two reactions were presumed to be connected if the magnitude of Pearson correlation for that reaction pair was >= 0.15. This adjacency matrix was represented as an undirected graph (correlation network) with reactions as nodes and correlation-based connections as edges between the nodes. Connected components were computed using the igraph package. All components that consist of at least three edges were considered to be a correlated (flux-coupled) subnetwork. Thereby, we obtained 52 flux-coupled subnetworks (modules) with a varying number of edges (Fig. 1C; Dataset EV1). We consider the major connected (correlated) network component to be the biomass reaction core, as it contained reactions related to canonical biomass precursor synthesis pathways known to occur in ECs (e.g., purine, pyrimidine synthesis, fatty acid oxidation).

#### Flux enrichment analysis

Flux enrichment analysis was performed using the hypergeometric one-sided test and FDR correction for multiple hypothesis testing. The metabolites associated with the biomass reaction core were mapped to obtain the corresponding correlated metabolite network or the biomass metabolite core. From this network, connections involving currency metabolites (Dataset EV1) were removed to facilitate the appropriate calculation of network topological properties. Using the CytoNCA app v.2.1.6 in Cytoscape v3.7.2, the eigenvector centrality of every metabolite in this processed network was calculated (Dataset EV1). A high eigenvector centrality score of a metabolite within a network indicates that a given metabolite is important because it is connected (correlated) to multiple, highly connected neighboring metabolites.

### Cell lines and culture

#### Human primary endothelial cells

Human umbilical vein endothelial cells (HUVECs, UZ Leuven, Leuven, Belgium) and Human umbilical artery endothelial cells (HUAECs, UZ Leuven, Leuven, Belgium) were freshly isolated from umbilical cords obtained from different donors under the ethical approval protocol S57123 (Ethics Committee Research UZ/KU Leuven) and with informed consent from all subjects, as previously described (De Bock et al, 2013; Schoors et al, 2015). Cells were isolated using standardized protocols as previously reported. Unless specified, ECs were routinely cultured in endothelial cell basal medium (EGM2; PromoCell, Heidelberg, Germany) supplemented with endothelial cell growth medium supplement pack (ECGM-2; PromoCell, Heidelberg, Germany) and 100 IU/mL penicillin and 100 mg/mL streptomycin (Thermo Fisher Scientific, Geel, Belgium). Cell cultures were regularly tested for mycoplasma contamination. In addition, cell identity was regularly authenticated by immunostaining for appropriate endothelial markers. In all experiments, HUVECs and HUAECs were used as single-donor cultures and were used between passages 1 and 4. Each experiment was performed with ECs from different donors (biological repeats). Unless otherwise indicated, experiments were done on subconfluent EC cultures. All experiments involving patient material were conducted in accordance with the ethical principles of the World Medical Association Declaration of Helsinki and the U.S. Department of Health and Human Services Belmont Report.

#### Endothelial and cancer cell lines

Murine bEnd3 and MCF7 cells were cultured in Dulbecco’s modified Eagle’s medium (DMEM; Thermo Fisher Scientific) supplemented with 10% fetal bovine serum (FBS), 100 IU/mL penicillin, and 100 µg/mL streptomycin. A549 and H2009 cells were cultured in RPMI medium (Gibco) supplemented with 10% FBS, 100 IU/mL penicillin, and 100 µg/mL streptomycin. Pericytes were cultured in Pericyte Growth Medium 2 (PromoCell). Human cardiac microvascular endothelial cells (HCMEC; PromoCell) were cultured in ECGM-MV Kit (PromoCell). Human coronary artery endothelial cells (HCAEC; Lonza) and human lung microvascular endothelial cells (HMVEC-L; Lonza) were cultured in EGM-2 BulletKit™ (Lonza). Cell lines were obtained from authenticated commercial repositories or suppliers. Established cell lines were obtained directly from the ATCC, whereas primary human endothelial cells and pericytes were purchased from Lonza or PromoCell. All cell lines were routinely tested and confirmed to be free of mycoplasma contamination. No additional authentication was performed after receipt.

### Knockdown strategy

#### In vitro

To generate short hairpin RNA (shRNA) vectors against *SLC25A12* and *SLC25A13*, oligonucleotides were cloned into the pLKO-shRNA2 vector (PLKO) (No. PT4052-5; Clontech). *SLC25A12* (NM_003705) and *SLC25A13* (NM_014251) shRNA were ordered from Origene. A nonsense scrambled shRNA sequence was used as a negative control. Lentiviral particles were produced in 293 T cells as previously described (Cantelmo et al, 2016). For lentiviral transduction of HUVECs, a multiplicity of infection (MOI) of 30 was used. Transductions were performed on day 0 and after 24 h the cells were refed with fresh medium. Unless otherwise indicated, all experiments were performed from day 3 or day 4 after transduction onwards. Knockdown efficiency was monitored for each experiment at the RNA and protein levels. To silence the expression of *SLC25A13* in vitro in bEnd3 cells, siRNA duplexes against (siSlc25a13; mm.Ri.Slc25a13.13.1) or control DsiRNA (siNC1) (IDT Integrated DNA Technologies) were used. To induce the silencing, bEnd3 cells were transfected using Lipofectamine RNAiMAX transfection kit (Thermo Fisher Scientific, catalog no.13778150) as described by the manufacturer with the following modifications: the volume of Lipofectamine was reduced by 30%, and the concentration of DsiRNA was increased to 20 μM (instead of the recommended 10 μM) in order to obtain an efficient knockdown with limited toxicity effect on the bEnd3 cells. Twenty hours after transfection with DsiRNA, the cells were washed and cultured for at least 24 h before use in the in vitro functional assays. The silencing efficiency was quantified by Western blot and quantitative RT-qPCR.

#### In vivo

To silence the target gene in the CNV model (see below) in vivo, siRNA duplexes directed against *Slc25a13* were tested for silencing in the murine bEnd3 cell line (ATCC), and the siRNA showing the best knockdown efficiency was selected for in vivo testing. This pre- selected siRNA duplex (siSlc25a13; mm.Ri.Slc25a13.13.1) or control DsiRNA (siNC1) (IDT Integrated DNA Technologies) was intravitreally injected (1 μg/eye, with the in vivo-jetPEI® reagent according to the manufacture’s instruction) in 7- to 10-week-old C57BL6/J mice immediately after induction of the laser burns (see below) and re-injected (to sustain the transient effect of siRNA treatment) (Guzman-Aranguez et al, 2013) at day 4 after induction of the laser burns with the same DsiRNA concentration (1 μg/eye) (Rohlenova et al, 2020; Schoors et al, 2014). On day 7 post-induction, the eyes were enucleated and processed for analysis of the neovascular area as described below. In order to check the genetic silencing in ECs upon DsiRNA treatment, on day 7 post-induction, choroidal ECs were isolated, single-cell suspensions were generated as described below, and alive, single-cell, CD45-, CD31+ , CD102+ choroidal ECs were FACS-sorted (BD FACSAria III). The sorted ECs were promptly subjected to RNA extraction for subsequent RT-qPCR analysis.

### RNA isolation and gene expression analysis

Total RNA was extracted using PureLink RNA mini kit (Invitrogen) or RNeasy Micro Kit (QIAGEN; for RNA isolation from choroidal ECs) according to the manufacturer’s instructions and converted to cDNA using the iScript cDNA synthesis kit (Bio-Rad) or SuperScript III First Strand cDNA synthesis kit (Thermo Fisher Scientific; for RNA isolation from choroidal ECs), respectively. RNA expression analysis was performed by Taqman Fast Universal PCR Master Mix (Thermo Fisher Scientific) using premade primer sets (Applied Biosystems, Carlsbad, CA and IDT, Belgium): *HPRT* Hs.PT.58.2145446 (NM_000194); *SLC25A12* Hs.PT.58.22913049 (NM_003705(2)); *SLC25A13* Hs.PT.58.25777150 (NM_001160210(3)); *Actb* Mm.PT.39a.22214843.g (NM_007393); *Slc25a13* Mm.PT.58.9683680 (NM_001177572(2)).

### Western blot

Protein extraction and Western blot analysis were performed using a modified RIPA lysis buffer (125 mM Tris-HCl (pH 7.4), 150 mM NaCl, 1% NP-40, 0.1% SDS, and 10% glycerol) in the presence of protease and phosphatase inhibitors (Roche). Lysates were separated by a 10% SDS-PAGE under reducing conditions, transferred to a nitrocellulose membrane, and analyzed by Western blot. Primary and secondary antibodies used were: rabbit anti- SLC25A12 (ab200201 Abcam; 1/500), rabbit anti-SLC25A13 (ab167166 Abcam; 1/500), rabbit anti-GAPDH (clone 14C10: #2118 Cell signaling; 1/1000), anti-rabbit Ig (#7074 Cell signaling; 1/2000), anti-mouse Ig (#7076 Cell signaling; 1/2000). All antibodies were used in 5% bovine serum albumin (BSA) diluted in TBST buffer. Signal was detected using the ECL and/or Femto systems (Amersham Biosciences, GE Healthcare) according to the manufacturer’s instructions. Densitometric quantification of bands was done with Fiji software (https://fiji.sc).

### In vitro functional assays

#### Proliferation

##### Thymidine incorporation

EC proliferation was quantified by incubating the cells with 1 mCi/mL [^3^H]-thymidine in full EGM2 as previously described (Kalucka et al, 2018). Thereafter, cells were fixed with 100% ethanol for 15 min at 4 °C, precipitated with 10% trichloroacetic acid for 10 min at 4 °C, and lysed with 0.1 N NaOH. The amount of [^3^H]-thymidine incorporated into DNA was measured by scintillation counting. [^3^H]-thymidine was added together with the supplement 6 h before cell lysis. The data were normalized to total protein content, determined by a bicinchoninic acid (BCA) assay.

##### EdU incorporation

Briefly, cells were labeled with 10 μM EdU over 24 h, collected by trypsinization, and fixed with 4% paraformaldehyde (Heirendt et al, 2019). The incorporated EdU was detected by a ‘Click-IT reaction’ with Alexa Fluor 647 according to the manufacturer’s instructions (Invitrogen, Life Technologies). Numbers of EdU-positive cells were measured in the different conditions using flow cytometry analysis.

##### Measuring cell confluence

Previously transduced HUVECs with *SLC25A13* or control shRNA constructs were seeded in the 24-well plate at low confluency (20%) in the EGM2 medium. The next day, the medium was refreshed, and the cells were immediately cultured at 37 °C at 5% CO_2_ in the Incucyte incubator, and live imaging (phase contrast) was performed every 2 h. The proliferation was quantified during 72 h after the medium refreshment, by confluency calculation (% relative to time 0—time when the supplemented medium was added). Rescuing cell confluence: Previously transduced HUVECs with *SLC25A13* or control shRNA constructs were seeded in the 24-well plate at low confluency (20%) in the EGM2 medium. Next day, the medium was replaced for EGM2 supplemented with (a) dNTPs (end concentration: 1 mM), (b) dNTPs (end concentration: 1 mM) + glutamic acid (end concentration: 3 mM), (c) glutamic acid (end concentration: 3 mM), (d) methyl-β-cyclodextrin cholesterol (end concentration: 0.09 mM) (Shu et al, 2019), (e) mevalonate (end concentration: 300 µM) or (f) PBS (control conditions). The cells were immediately placed in the 37 °C Incucyte incubator with 5% CO_2_ and monitored by phase contrast imaging at a 2 h interval. The proliferation was quantified 24 or 72 h after the cells were incubated with supplemented medium, by confluency calculation (% relative to time 0 – time when the supplemented medium was added).

#### Apoptosis and cell cycle analysis

The Annexin V/Propidium Iodide (PI) staining kit was used to assess cell apoptosis according to the manufacturer’s protocol. Cells were harvested, washed twice with cold PBS, and resuspended in binding buffer. In total, 5 µL of Annexin V-FITC and 5 µL of PI were added to 100 µL of the cell suspension. The mixture was gently vortexed and incubated for 15 min at room temperature in the dark. After incubation, 400 µL of binding buffer was added, and the samples were immediately analyzed by flow cytometry.

Cell cycle analysis was performed using Propidium Iodide (PI) staining according to the standard protocol. Cells were collected, washed twice with cold PBS, and fixed in 70% ethanol for 30 min. After fixation, cells were washed again with PBS and treated with RNase A (100 µg/mL). Finally, cells were stained with PI (50 µg/mL). DNA content was analyzed by flow cytometry, and cell cycle distribution was determined by quantifying the percentage of cells in G0/G1, S, and G2/M phases using Flowjo.

#### Scratch wound migration assay

A scratch wound was applied on confluent EC monolayers (pretreated for 8 h with 1 μg/mL Mitomycin C to block proliferation) using a 20 μL tip, 24 h after seeding. After scratch wounding and photography (T0), the cultures were further incubated in fully supplemented EGM2 medium for 16 h (until near closure was reached in the control condition) and imaged again (T16) using a Leica DMI6000 microscope (Leica Microsystems). Migration was measured with the NIH ImageJ software package and is expressed as a percentage of wound closure (gap area at T0 minus gap area at T16 in % of gap area at T0).

#### Spheroid capillary sprouting assay

In all, 1000 ECs were incubated overnight in 25 μL hanging drops of EGM-2 medium containing methylcellulose (20% volume of a 1.2% solution of methylcellulose 4000 cP) (Sigma-Aldrich, Bornem, Belgium) to form spheroids. For mitotic inactivation, Mitomycin C (2 μg/mL) was added to this medium. Spheroids were then embedded in collagen gel as described (De Bock et al, 2013) and cultured for 20 h to induce sprouting. Cultures were fixed with 4% PFA at room temperature and imaged under bright field using a Leica DMI6000 microscope (Leica Microsystems, Mannheim, Germany). Analysis of the number of sprouts per spheroid and the total sprout length (cumulative length of primary sprouts and branches per spheroid) was performed on phase contrast images using the Fiji/ImageJ analysis software.

#### Intracellular ROS analysis

Intracellular ROS levels were measured using CM-H2DCFDA (Thermo Fisher Scientific) according to the manufacturer’s instructions. The ROS levels were determined by pre-incubation of the ECs for 30 min with the dye in serum-free M199 medium (Thermo Fisher Scientific) (Garcia-Caballero et al, 2019). The fluorescent intensity was measured by flow cytometry. Data were analyzed using FlowJo software.

#### NADH/NAD assay

NAD and NADH levels were measured using the NAD/NADH Assay Kit (Sigma-Aldrich, Cat. No. MAK4681KT) following the manufacturer’s instructions. Samples were prepared by homogenizing cells in the NAD or NADH Extraction Buffer provided in the kit and heated at 60 °C for 5 min. The homogenates were centrifuged at 14,000×*g* for 5 min at 4 °C. Absorbance was measured at 450 nm using a microplate reader, and concentrations were calculated from a standard curve generated with the NADH standard provided in the kit.

### Bulk RNA-sequencing

#### RNA extraction and sequencing

RNA of scrambled control and SLC25A13^KD^ ECs was extracted 6 days after seeding using the TRIzol kit (Life Technologies) following the manufacturer’s instructions; quality and quantity were measured on a Nanodrop (Thermo Fisher Scientific). Sequencing libraries were prepared with the Illumina TruSeq Stranded mRNA Library prep kit according to the manufacturer’s protocol. Samples were indexed to allow for multiplexing. Library quality and size range were assessed using a Bioanalyzer (Agilent Technologies) with the DNA 1000 kit (Agilent Technologies) according to the manufacturer’s recommendations. Libraries were sequenced on an Illumina NovaSeq6000 platform according to the manufacturer’s recommendations. Single-end reads of 100 bp length were produced with a minimum of 20 M reads per sample. Quality control of raw reads was performed with FastQC v0.11.9. TruSeq adapters were filtered with Trimmomatic 0.39 using default parameters. Splice-aware alignment was performed with STAR v2.6.1 d against the human reference genome hg38 using the default parameters (Dobin et al, 2013). Reads mapping to multiple loci in the reference genome were discarded. Quantification of reads per gene was performed with HT- Seq v2.7.14. Sample-wise quantified reads were integrated into a gene expression matrix, which was provided for further downstream processing and analysis. The gene expression accounted for 12 samples (6 for CTRL and 6 for SLC25A13^KD^ ECs).

#### Normalization and differential gene expression analysis

All genes that were expressed in at least 6 of the total 12 samples and had at least 5 reads were retained for further analysis. The ENSEMBL gene IDs were first mapped to HUGO gene symbols using the *bioMaRt* package. Next, gene expression was normalized across samples using the trimmed mean of *M*-values (TMM) normalization implemented in the *edgeR* package. Differential gene expression analysis was performed to compare the transcriptomes of control ECs transduced with scrambled RNA (CTRL) with SLC25A13^KD^ ECs, again using the *edgeR* package.

#### Self-contained gene set enrichment analysis

The rotation gene set tests (ROAST) implemented in the *limma* package were performed on 25 metabolic gene-sets extracted from the subsystem slot of the Recon3D human metabolic reconstruction (Brunk et al, 2018; Wu et al, 2010). The enrichment analysis was performed by computing the weighted mean of the gene-wise statistics and also correcting for *P* values using the *mroast* function. The average gene-wise t-statistic for each process was plotted and compared visually by generating a lollipop plot in the *ggplot* package. The mentioned analyses for transcriptome data analysis were performed in R v.4.0.1.

### Single-cell transcriptome dataset analysis and radial plots

Radial plots were generated using publicly available single-cell or single-nucleus RNAseq (sc-/sn-RNAseq) data from inflammatory and tumor datasets (incorporating publications until December 2021 and October 2023, respectively). The radial plots indicate the PMIDs of all datasets included in the analysis (Fig. EV5A,B). Original papers were included only if they contained sc-/sn-RNAseq data on freshly isolated (non-cultured) human cells. Studies that did not contain ECs or for which the data were not publicly available were excluded. Datasets with unprocessed raw sequence reads, with low quality data or metadata, from non-adult (i.e. <16 years of age) samples, with less than 750 ECs after in silico EC selection were excluded. The following quality control steps were performed for each dataset: (i) exclusion of genes expressed in less than 3 cells (or nuclei) were removed; (ii) removal of cells (or nuclei) expressing less than 250 genes (low quality) and cells (or nuclei) expressing more than 6000 genes (potential doublets); (iii) removal of cells (or nuclei) with a detected fraction of mitochondrial genes >20% (indicative of low quality cells, likely due to cell damage/death). Endothelial clusters were annotated based on the expression of canonical EC and non-EC marker genes, and only the in silico selected ECs were used to perform downstream analyses. The results from differentially expressed genes (DEG) analyses were used to construct radial plots. Differential gene expression analyses were performed using the *FindMarkers()* function, as provided in Seurat v5, comparing normalized gene expression data in the experimental group versus the control group within each individual dataset. Genes with an adjusted *P* value ≤ 0.05 and a log2FC ≥ 0.25 (upregulated DEGs) or ≤ −0.25 (downregulated DEGs) were considered differentially expressed. Datasets were ranked on the radial plot by log2FC (log2FC determined both the color as well as the position of the marker on the radial axis) and marker size was determined based on the adjusted *P* value (crosshair marker for non-significant results, dots for significant results, with dot sizes scaled by adjusted *P* value (<0.0001, <0.001, <0.01 or <0.05 corresponding to sizes 50, 30, 20, or 15 respectively).

### Metabolomics

#### ^13^C tracer experiments and metabolite levels

For ^13^C-carbon incorporation from glutamate in metabolites, cells were incubated for 48 h with [U-^13^C] glutamine in a 100% labeling way (glucose: 5.5 mM; glutamine: 2 mM). Metabolites from 3 × 10^5^ cells were extracted in 300 μL cellular extraction buffer (80% methanol with 2 μM d27 myristic acid). Next, the extracts were centrifuged at 4 °C for 15 min at 20,000 × *g*, and the supernatants were used for Liquid chromatography–mass spectrometry (LC–MS) (ion-pairing mode). Measurements were performed using a Dionex UltiMate 3000 LC System (Thermo Fisher Scientific) in-line connected to a Q-Exactive Orbitrap mass spectrometer (Thermo Fisher Scientific) (Huang et al, 2017). 40 μL of sample was injected and loaded onto a SeQuant ZIC/pHILIC Polymeric column (Merck-Millipore). A linear gradient was carried out starting with 84% solvent A (95-5 acetonitrile- H_2_O, 2 mM ammonium acetate pH 9.3) and 16% solvent B (2 mM ammonium acetate pH 9.3). From 2 to 29 min, the gradient changed to 75% B and was kept at 75% until 34 min. Next, a decrease to 16% B was carried out to 42 min, and then, 16% B was maintained until 58 min. The solvent was used at a flow rate of 100 μL/min, and the column temperature was kept constant at 25 °C. The mass spectrometer operated in negative ion mode; settings of the HESI probe were as follows: sheath gas flow rate at 25, auxiliary gas flow rate at 5 (at a temperature of 260 °C). Spray voltage was set at 4.8 kV, temperature of the capillary at 300 °C, and S-lens RF level at 50. Normal full scan (resolution of 35,000 and scan range of *m/z* 50–1050) was applied. In case of low metabolite levels (such as dNTPs), a targeted Selected Ion Monitoring (SIM) method was applied. Data collection (total metabolite abundances as well as ^13^C isotopic incorporation) was performed with the Xcalibur software (Thermo Fisher Scientific). For the calculation of the total carbon/nitrogen contribution, we corrected for naturally occurring isotopes using the method of Fernandez et al (Fernandez et al, 1996). For relative metabolite levels, the total ion count was normalized to the protein content. The total contribution of carbon/nitrogen was calculated using the following equation (Fendt et al, 2013):

$${{Total}}\,{{contribution}}\,{{of}}\,{{carbon}}={\sum }_{i=0}^{n}i\times {m}_{i}/\left(n{\sum }_{i=0}^{n}{m}_{i}\right)$$

Where *n* is the number of carbons in each metabolite, *I* represents the different mass- isotopomers, and *m* refers to the abundance of a certain mass. The fractional contribution of a metabolite is expressed as a percentage for relative comparison.

#### ^13^C glucose labeling and G6P, cholesterol quantification

For split injection gas chromatography-mass spectrometry (GC–MS) methods, 50  μL of the supernatant was transferred and evaporated to dryness on a speedvac. A 2% methoxyamine (MOX) solution was prepared by dissolving methoxyamine hydrochloride (Supelco, ref 226904-5 g) in pyridine (Sigma-Aldrich, ref 270970-1 L). 12.5 MOX solution was added to each sample, then left to react for 1.5 h at 37 °C for methoximation of aldehyde and ketone groups. Afterward, 40 N,O-Bis(trimethylsilyl)trifluoroacetamide (BSTFA) with 1% trimethylchlorosilane (TMCS) (Supelco, ref 15238) was added and left to react for 45 min at 60 °C for trimethylsilylation of amino, alcohol, or acid groups. The samples were left in the dark overnight to maximize the reaction efficiency; then they were divided into 2 aliquots in different mass spec vials for GC-EI-MS and GC-SICRIT-MS analyses.

One microliter of each methoximated and trimethylsilylated sample was injected by 1:1 split injection by a TriPlus RSH SMART Autosampler autosampler (Thermo Fischer Scientific, Waltham, MA USA) into an Trace 1610 gas chromatograph equipped with a DB-5MS-DG column (30 m 0.25 mm i.d fused silica capillary column with a chemically bonded 0.25 µm DB-5MS stationary phase with additional 10 m duraguard column, Agilent J&W GC columns, Folsom; California, ref 122-5532 G). The injector temperature was 250 °C, and the septum purge flow rate was 3 mL/min. The helium flow rate through the column was 1.55 mL/min, the column temperature was held at 100 °C for 2 min, then increased by 20 °C/min to 175 °C, then by 4 °C/min to 230 °C, and was held there for 3 min. Subsequently, the temperature was increased by 40 °C/min to 300 °C, held for 5.75 min, then by 100 °C to 325 °C and held for 1.75 min. The GC was linked by a transfer line (Plasmion GmbH, Augsburg, Germany) to the SICRIT GC/SPME module (Plasmion GmbH, Augsburg, Germany), which serves to guide the helium gas exiting the GC column to a SICRIT cold plasma ion source (Plasmion GmbH, Augsburg, Germany), installed on an Exploris 120 Orbitrap mass spectrometer (Thermo Fischer Scientific, Waltham, MA, USA). The transferline and GC/SPME module were heated to 280 °C, while the MS supplied a N_2_ gas stream of 2.31 L/min (2 arbitrary units in the Exploris 120 Tune Application) that was humidified by bubbling through milliQ water before entering the GC/SPME module. There, it carried the column effluent to the plasma in the SICRIT ion source. The plasma was generated by an AC electric field of 1500 V at 15 kHz. This soft ionization setup allows primarily [M + H + ]+ ions to be generated. The transfer capillary temperature was 250 °C. The mass spectrometer operated in positive mode with three scan ranges at 15k resolution: (1) [90–900] at 1 microscan with AGC target at at 100% at max injection time at for minutes 6–30 to detect internal standards and lighter molecules; (2) [400–1200] at 3 microscans with AGC target at 1000% at max injection time at 90 ms for the internal standards for minutes 6–28.5 for sensitive detection of heavier molecules; (3) [335–1200] at 3 microscans with AGC target at 1000% at max injection time at 90 ms for the internal standards for minutes 28.5–30 for sensitive detection of heavier molecules including TMS- cholesterol that loses TMS-OH upon ionization. Data collection was performed using the Xcalibur software (Thermo Scientific). The data analyses were performed by integrating the peak areas of unfragmented ions (El-Maven–Elucidata).

#### Relative abundance of metabolites

The relative change of metabolite abundances (asparagine, proline, N-carbamoyl-L-aspartate, thymidine triphosphate, uridine diphosphate, mevalonate, NAD + , NADH) was calculated between the CTRL and SLC25A13^KD^ ECs. The raw abundance of a given metabolite is calculated as the sum of all isotopologue areas per metabolite noted in raw intensity and is normalized to the protein content of a cell, determined by the BCA assay.

#### Cellular energy status

HUVECs (CTRL and SLC25A13^KD^) were collected in 300 μL cold (chilled on dry ice) 50:30:20 (methanol: acetonitrile: 20 mM Tris, pH 9.3) extraction buffer, and 100 μL of the mixture was injected onto an Agilent 1260 HPLC equipped with a C18-Symmetry column (150 × 4.6 mm; 5 μm) (Waters), thermostatted at 22.5 °C (Kalucka et al, 2018). The flow rate was kept constant at 1 mL/min. A linear gradient using solvent A (50 mM NaH_2_PO_4_, 4mMtetrabutylammonium, adjusted to pH 5.0 using H_2_SO_4_) and solvent B (50 mM NaH_2_PO_4_, 4 mM tetrabutylammonium, 30% CH_3_CN, adjusted to pH 5.0 using H_2_SO_4_) was accomplished as follows: 95% A for 2 min, from 2 to 25 min linear increase to 100% B, from 25 to 27 min isocratic at 100% B, from 27 to 29 min linear gradient to 95% A and finally from 29 to 35 min at 95% A. Detection of ATP, ADP and AMP occurred at 259 nm (Kalucka et al, 2018). The energy charge was calculated using the following equation:

$${Energy}\,{charge}=\frac{\left[{ATP}\right]\,+\,\frac{1}{2}\,[{ADP}]}{\left[{ATP}\right]\,+\,\left[{ADP}\right]\,+\,[{AMP}]}$$

### Oxygen consumption

HUVECs (CTRL and SLC25A13^KD^) were seeded at 1.5 × 10^5^ cells per well on Seahorse XF24 tissue culture plates (Seahorse Bioscience Europe). Oxygen consumption (OCR) measurements were performed at 6-min intervals (2 min mixing, 2 min recovery, 2 min measuring) in a Seahorse XF24 device (XF Reader 1.8.1.1 software). Treatment with oligomycin (1.2 μM final) was performed to enable quantification of ATP-coupled OCR (OCRATP).

### Glucose oxidation assay

HUVECs (CTRL and SLC25A13^KD^) were incubated for 6 h in EGM2 medium containing 0.55 μCi/mL [6-^14^C]D-glucose. After that, 250 μL of 2 M perchloric acid was added to each well to stop cellular metabolism and to release ^14^CO_2_, which was captured overnight at room temperature in 1× hyamine hydroxide-saturated Whatman paper. The radioactivity in the paper was determined by liquid scintillation counting (QuantaSmart V4 PerkinElmer).

### Glutamine oxidation assay

HUVECs (CTRL and SLC25A13^KD^) were incubated for 6 h with EGM2 medium containing 0.5 μCi/mL [U-^14^C] glutamine. In total, 250 mL of 2 M perchloric acid was added to the cells to stop cellular metabolism and release ^14^CO_2_. Trapping of ^14^CO_2_ was done as described for ^14^C-glucose oxidation.

### Fatty acid oxidation assay

HUVECs (CTRL and SLC25A13^KD^) were incubated in fully supplemented EGM2 medium with 100 μM unlabeled palmitate and 50 μM carnitine. Cells were incubated for 2 h in growth medium containing 2 μCi/mL [9,10-^3^H]-palmitate (Dagher et al, 2001; Schoors et al, 2015; Wang et al, 2011). Thereafter, the supernatant was transferred into glass vials sealed with rubber stoppers. ^3^H_2_O was captured in hanging wells containing a Whatman paper soaked with H_2_O over a period of 48 h at 37 °C to reach saturation. Radioactivity was determined by liquid scintillation counting.

### Glycolysis flux measurements

HUVECs (CTRL and SLC25A13^KD^) were cultured for 6 h in EGM2 medium supplemented with 0.4 μCi/mL [5-^3^H] D-glucose (PerkinElmer), after which the supernatant was transferred into glass vials sealed with rubber stoppers. ^3^H_2_O was captured in hanging wells containing a Whatman paper soaked with H_2_O for 48 h at 37 °C to reach saturation. The paper was then used for liquid scintillation counting (QuantaSmart V4 PerkinElmer).

### RNA synthesis assay

Transduced HUVECs were seeded at 30,000 cells/well in a 24-well plate on 0.1% gelatin-coated glass bottom plates in full EGM2 medium. RNA synthesis was determined by incubation of cells with a uridine analog (5′-ethynyl uridine, EU, Molecular Probes) for 24 h in full medium. Cells were trypsinized, PFA fixed, and staining with RNA label/DAPI was performed according to the manufacturer’s instructions. The plates were then analyzed by fluorescence microscope imaging using a Zeiss LSM780 confocal microscope (Carl Zeiss) with five fields of vision per well at ×20 magnification.

### Protein synthesis assay

Transduced HUVECs were seeded at 30,000 cells/well in a 24-well plate on 0.1% gelatin-coated glass bottom plates in methionine-free EGM2 medium. Protein synthesis was determined by incubation of cells with a methionine analog (50 µM homopropargylglycine) for 24 h in full medium. Cells were trypsinized, PFA fixed, and incorporated HPG was detected by a Click-IT reaction with Alexa fluor 647 according to the manufacturer’s instructions. The plates were then analyzed by fluorescence microscope imaging using a Zeiss LSM780 confocal microscope (Carl Zeiss) with 5 fields of vision per well at ×20 magnification.

### In vivo choroidal neovascularization (CNV) mice

#### Mouse model of choroidal neovascularization

Experiments were performed in 7- to 10-week-old C57BL6/J mice obtained from the KU Leuven animal facility. No statistical method was used to predetermine the sample size. Animal housing and all experimental procedures were approved by the Institutional Animal Ethics Committee of the KU Leuven (Belgium) under protocol number P077/2021. Choroidal neovascularization (CNV) was induced by laser burns as previously described (Schoors et al, 2014) using a Purepoint Laser (Alcon, Fort Worth, USA). Ten or eight impacts (for choroidal endothelial cell isolation or histological analysis, respectively), rupturing the Bruch’s membrane, were made around the optical nerve using a laser with a diameter of 100 μm, power 0.320 W, and exposure time 0.05 s in both eyes. On day 7, at the height of the angiogenic response (Lambert et al, 2013; Martinez and Peplow, 2021), mice were euthanized by cervical dislocation (three mice (six eyes) per condition), and the eyes were enucleated by inserting scissors along the eye into the orbital cavity. The four optical muscles and the optic nerve, which appear as a white cord behind the eye, were cut. The dissected eyes were then placed in PBS, and the periocular tissue was removed. The retinal pigment epithelium (RPE)-choroid-sclera complex was dissected from the enucleated eyes by peeling off the vitreous body and retina. For histological analysis, the eyes were collected 10 min after retrobulbar injection with Fluorescein isothiocyanate (FITC)-conjugated dextran (Mr 2,000,000) (Sigma-Aldrich) and fixed in 2% PFA. Choroids were dissected, flat-mounted (ProLong Gold antifade reagent, Thermo Fisher Scientific), and imaged using a Leica TCS SPE confocal microscope (Leica Microsystems). Analysis of the neovascular area was performed with the Leica MM AF morphometric analysis software (Leica Microsystems) and expressed as the FITC–dextran positive area in percent of the total CNV lesion area. This procedure was repeated for 4–5 independent replicate experiments, each with three mice (six eyes) per condition.

#### Murine choroid EC isolation

The isolation of the choroids was performed as described above, and choroidal endothelial cells were isolated based on the protocol published by (Conchinha et al, 2021), omitting the magnetic cell sorting steps. The choroids were dissociated into single-cell suspension in a digestion buffer (0.3% (w/v) collagenase I, DNase I (7.5 μg/mL) and dispase (0.25 U/mL) in Knock-OutTM DMEM-medium (Thermo Fisher Scientific) supplemented with 1 mM sodium pyruvate, 1× MEM NEAAs, ECGF/Heparin, antibiotic/antimycotic (2x) and 1% (v/v) penicillin/streptomycin) for 30–40 min at 37 °C with manual pipetting every 10 min. The reaction was stopped with 5 mL of wash buffer, and the cell suspension was filtered through a 100 and 40-μm cell strainer. Next, choroidal ECs were sorted by FACS (Conchinha et al, 2021). To do so, single-cell suspensions were stained with fluorescently labeled antibodies (CD45 (PE-Cy7, dilution 1:500), CD31 (AF488, dilution 1:100), CD102/ICAM2 (APC, dilution 1:50)) and a viability dye (VD, eFluorTM 450, dilution 1:1000, labeling dead cells) for 30 min. Next, FACS-sorted viable single cells (VD − , CD45 − , CD102+, and CD31+) were directly collected into lysis buffer from the RNeasy Micro Kit (Qiagen). We based the selection of ECs on both CD31 and CD102 to increase purity. The choroidal ECs were sorted using fluorescence sorting as described in Conchinha et al, 2021 and subjected to RNA isolation (RNeasy Micro Kit (QIAGEN); SuperScript III First Strand cDNA synthesis kit (Thermo Fisher Scientific)) and quantitative RT-qPCR (see above). This procedure was repeated for 4–5 independent replicate experiments, each with three mice (six eyes) per condition.

### Statistical analysis and visualization

Unless otherwise stated, statistical analyses and bar plot visualizations related to the in vitro and in vivo experiments were performed using GraphPad Prism v.8. No blinding was performed during the in vitro or in vivo experiments. Primary HUVECs from each donor were allocated to parallel control and knockdown treatment conditions according to the experimental design. For the in vivo studies, mice were randomly assigned to receive either control siRNA or siRNA targeting the gene of interest. Samples were included in the analysis only when effective knockdown of the target gene was confirmed (>40% reduction); samples failing this criterion were excluded from the corresponding analysis. Comparison of conditions was performed using two-tailed *t* test or one-sample *t* test as indicated in the figure legends. For HUVECs, individual donors were used with parallel control and KD treatment, and therefore, a pairwise comparison was performed. Comparison between multiple groups was performed using one-way ANOVA. A *P* value < 0.05 was considered statistically significant. The scheme of the catabolic pathways predicted from EC-GEM was generated in Inkscape v.0.91. The synopsis figure was created using BioRender (under a licensed subscription). The flux correlation network figure was generated in Cytoscape v.3.7.2. The lollipop plots were generated using the ggplot R package. The heatmap for comparing gene expression across samples was generated using the ComplexHeatMap R package.

## Supplementary information

Peer Review File

Dataset EV1

Dataset EV2

Source data Fig. 1

Source data Fig. 2

Source data Fig. 3

Source data Fig. 4

Expanded View Figures

## Data Availability

Raw sequencing data generated in this article are available in ArrayExpress under accession number E-MTAB-14591 (https://seqout.org/p/E-MTAB-14591). The EC-GEM model, together with the script files, is available in Zenodo at https://doi.org/10.5281/zenodo.21817828. Raw and derived metabolomics data are available in MetaboLights under accession number MTBLS15306. The source data of this paper are collected in the following database record: biostudies:S-SCDT-10_1038-S44321-026-00524-3.
