# Supplementary material for: Endothelial SLC25A13 links glutamine metabolism to angiogenesis
Source: EMBO Mol Med. 2026 Sep 26;18(11):4357–85. doi: 10.1038/s44321-026-00524-3 (PMC13748631; doi:10.1038/s44321-026-00524-3)
Supplement: Supplementary file 1 [file 44321_2026_524_MOESM1_ESM.pdf]

EMBO Molecular Medicine

ENDOTHELIAL SLC25A13 LINKS GLUTAMINE METABOLISM TO ANGIOGENESIS

Abhishek Subramanian, Anne Cuypers, Patricia Yagüe Sanz, Mathias Declercq, Joris Souffreau, Pieter-Jan Coolen, Liliana Sokol, Anh-Co Truong, Mila Borri, Koen Veys, Sabine Wyns, Els Gils, Stefan Vinckier, Katleen Brepoels, Ling Jin, Huberte Moreau, Mira Mousa, Bart Ghesquière, Baharak Hosseinkhani, Guy Eelen, Mieke Dewerchin, Massimiliano Mazzone, and Peter Carmeliet

Corresponding author: Peter Carmeliet (peter.carmeliet@kuleuven.be)

Review Timeline:

Submission Date:	4th Nov 24
Editorial Decision:	27th Nov 24
Revision Received:	1st May 26
Editorial Decision:	29th May 26
Revision Received:	16th Jul 26
Editorial Decision:	24th Jul 26
Revision Received:	21st Aug 26
Accepted:	28th Aug 26

Editor: Lise Roth

Transaction Report:

(Note: With the exception of the correction of typographical or spelling errors that could be a source of ambiguity, letters and reports are not edited. Depending on transfer agreements, referee reports obtained elsewhere may or may not be included in this compilation. Referee reports are anonymous unless the Referee chooses to sign their reports.)

27th Nov 2024

Dear Prof. Carmeliet,

Thank you for the submission of your manuscript to EMBO Molecular Medicine. We have now heard back from the referees who agreed to evaluate your manuscript. As you will see below, the reviewers raise substantial concerns on your work, which unfortunately preclude its publication in EMBO Molecular Medicine in its current form.

Briefly, referee #1 is overall supportive of revisions, but requests experiments on the role of SLC25A13 in cell cycle progression and cell death, as well as information on the potential interplay between SLC25A13 and SLC25A12, on the type of EC expressing SLC25A13 and cellular localization, and discussion on the role of SLC25A13 and its medical relevance. Referee #2 is more critical and finds that the manuscript has major flaws, including limited novelty, descriptive nature, unproven endothelial specificity, need for additional types of endothelial cells and for additional in vivo models of physiological angiogenesis, deeper metabolic analyses, etc. Finally, ref. #3 is supportive of the manuscript but raises questions on the initial modelling, and use of a single type of endothelial cells.

If you feel you can satisfactorily address the points listed by the referees, you may wish to submit a revised version of your manuscript. Please attach a covering letter giving details of the way in which you have handled each of the concerns. A revised manuscript will once again be subject to review, and we cannot guarantee at this stage that the eventual outcome will be favorable.
Revising the manuscript according to the referees' recommendations appears to require a lot of additional work and experimentation, and given the potential interest of your findings, we are ready to extend the deadline to 6 months.

If you would like to discuss further the points raised by the referees or your revision plan, I am available to do so via email or video. Let me know if you are interested in this option.

EMBO Molecular Medicine encourages a single round of revision only and therefore, acceptance or rejection of the manuscript will depend on the completeness of your responses included in the next, final version of the manuscript. For this reason, and to save you from any frustrations in the end, I would strongly advise against returning an incomplete revision. Should you find that the requested revisions are not feasible within the constraints outlined here and prefer, therefore, to submit your paper elsewhere, we would welcome a message to this effect.

When submitting your revised manuscript, please carefully review the instructions that follow below. We perform an initial quality control of all revised manuscripts before re-review; failure to include requested items will delay the evaluation of your revision.

We require:

1) A .docx formatted version of the manuscript text (including legends for main figures, EV figures and tables). Please make sure that the changes are highlighted to be clearly visible.

2) Individual production quality figure files as .eps, .tif, .jpg (one file per figure). For guidance, download the 'Figure Guide PDF' (https://www.embopress.org/page/journal/17574684/authorguide#figureformat).

3) At EMBO Press we ask authors to provide source data for the main figures. Our source data coordinator will contact you to discuss which figure panels we would need source data for and will also provide you with helpful tips on how to upload and organize the files.

4) A .docx formatted letter INCLUDING the reviewers' reports and your detailed point-by-point responses to their comments. As part of the EMBO Press transparent editorial process, the point-by-point response is part of the Review Process File (RPF), which will be published alongside your paper.

5) A complete author checklist, which you can download from our author guidelines (https://www.embopress.org/page/journal/17574684/authorguide#submissionofrevisions). Please insert information in the checklist that is also reflected in the manuscript. The completed author checklist will also be part of the RPF.

6) All Materials and Methods need to be described in the main text using our 'Structured Methods' format. According to this format, the Methods section includes a Reagents and Tools Table (listing key reagents, experimental models, software and relevant equipment and including their sources and relevant identifiers) followed by a Methods and Protocols section describing the methods, ideally using a step-by-step protocol format. The aim is to facilitate adoption of the methodologies across labs. Please download and fill our Reagents and Tools Table template (.docx), which you can find in our author guidelines: https://www.embopress.org/page/journal/14693178/authorguide#structuredmethods.
When submitting your revised manuscript, please do not include the Reagents and Tools Table in the Methods section of the

manuscript but upload it as a separate file choosing the file type "Reagent Table".
An example of a Method paper with Structured Methods can be found here:
https://www.embopress.org/doi/10.15252/msb.20178071

7) It is mandatory to include a 'Data Availability' section after the Materials and Methods. Before submitting your revision, primary datasets produced in this study need to be deposited in an appropriate public database, and the accession numbers and database listed under 'Data Availability'. Please remember to provide a reviewer password if the datasets are not yet public (see https://www.embopress.org/page/journal/17574684/authorguide#dataavailability).

In case you have no data that requires deposition in a public database, please state so in this section. Note that the Data Availability Section is restricted to new primary data that are part of this study.

8) For data quantification: please specify the name of the statistical test used to generate error bars and P values, the number (n) of independent experiments (specify technical or biological replicates) underlying each data point and the test used to calculate p-values in each figure legend. The figure legends should contain a basic description of n, P and the test applied. Graphs must include a description of the bars and the error bars (s.d., s.e.m.). Please provide exact p values.

9) Our journal encourages inclusion of *data citations in the reference list* to directly cite datasets that were re-used and obtained from public databases. Data citations in the article text are distinct from normal bibliographical citations and should directly link to the database records from which the data can be accessed. In the main text, data citations are formatted as follows: "Data ref: Smith et al, 2001" or "Data ref: NCBI Sequence Read Archive PRJNA342805, 2017". In the Reference list, data citations must be labeled with "[DATASET]". A data reference must provide the database name, accession number/identifiers and a resolvable link to the landing page from which the data can be accessed at the end of the reference. Further instructions are available at .

10) We replaced Supplementary Information with Expanded View (EV) Figures and Tables that are collapsible/expandable online. A maximum of 5 EV Figures can be typeset. EV Figures should be cited as 'Figure EV1, Figure EV2" etc... in the text and their respective legends should be included in the main text after the legends of regular figures.
- For the figures that you do NOT wish to display as Expanded View figures, they should be bundled together with their legends in a single PDF file called *Appendix*, which should start with a short Table of Content. Appendix figures should be referred to in the main text as: "Appendix Figure S1, Appendix Figure S2" etc.
- Additional Tables/Datasets should be labeled and referred to as Table EV1, Dataset EV1, etc. Legends have to be provided in a separate tab in case of .xls files. Alternatively, the legend can be supplied as a separate text file (README) and zipped together with the Table/Dataset file.
See detailed instructions here:

11) The paper explained: EMBO Molecular Medicine articles are accompanied by a summary of the articles to emphasize the major findings in the paper and their medical implications for the non-specialist reader. Please provide a draft summary of your article highlighting
- the medical issue you are addressing,
- the results obtained and
- their clinical impact.
This may be edited to ensure that readers understand the significance and context of the research. Please refer to any of our published articles for an example.

12) Author contributions: CRediT has replaced the traditional author contributions section because it offers a systematic machine readable author contributions format that allows for more effective research assessment. Please remove the Authors Contributions from the manuscript and use the free text boxes beneath each contributing author's name in our system to add specific details on the author's contribution. More information is available in our guide to authors.

13) Disclosure statement and competing interests: We updated our journal's competing interests policy in January 2022 and request authors to consider both actual and perceived competing interests. Please review the policy https://www.embopress.org/competing-interests and update your competing interests if necessary.

14) Every published paper now includes a 'Synopsis' to further enhance discoverability. Synopses are displayed on the journal webpage and are freely accessible to all readers. They include a short stand first (maximum of 300 characters, including space) as well as 2-5 one-sentences bullet points that summarizes the paper. Please write the bullet points to summarize the key NEW findings. They should be designed to be complementary to the abstract - i.e. not repeat the same text. We encourage inclusion of key acronyms and quantitative information (maximum of 30 words / bullet point). Please use the passive voice. Please attach these in a separate file or send them by email, we will incorporate them accordingly.

Please also suggest a visual abstract to illustrate your article as a PNG file 550 px wide x 300-600 px high. A cropped portion of this image will serve as thumbnail for the table of content on our webpage.

15) As part of the EMBO Publications transparent editorial process initiative (see our Editorial at http://embomolmed.embopress.org/content/2/9/329), EMBO Molecular Medicine will publish online a Review Process File (RPF) to accompany accepted manuscripts.
In the event of acceptance, this file will be published in conjunction with your paper and will include the anonymous referee reports, your point-by-point response and all pertinent correspondence relating to the manuscript. Let us know whether you agree with the publication of the RPF and as here, if you want to remove or not any figures from it prior to publication.
Please note that the Authors checklist will be published at the end of the RPF.

EMBO Molecular Medicine has a "scooping protection" policy, whereby similar findings that are published by others during review or revision are not a criterion for rejection. Should you decide to submit a revised version, I do ask that you get in touch after three months if you have not completed it, to update us on the status.

I look forward to receiving your revised manuscript.

Yours sincerely,

Lise Roth

Lise Roth, PhD
Senior Editor
EMBO Molecular Medicine

***** Reviewer's comments *****

Referee #1 (Comments on Novelty/Model System for Author):

excellent methodology. The weak point is that no EC-specific knockout mouse was generated.

Referee #1 (Remarks for Author):

This manuscript reports for the first time about a role of the glutamate-aspartate antiporter SLC25A13 in endothelial cells. The Carmeliet group used their previously described large data sets of endothelial gene expression and bioinformatic tools to predict genes controlling endothelial cell metabolism including SLC25A13. There are no publications in PubMed reporting about this gene in endothelial cells. The authors studied this gene using shRNAs to inhibit its expression in primary endothelial cell cultures. In addition, they treated the mouse retina (AMD model) with such constructs to achieve a gene knockdown, however not exclusively in endothelial cells. The experiments revealed that this glutamate transporter plays important roles for biomass production including cholesterol, a finding which was not reported before in other cell types. A thorough state of the art investigation of cellular metabolism confirmed this and suggested the biochemic mechanism. In addition, silencing of SLC25A13 impaired endothelial proliferation, migration and tube formation. As such the data are very interesting. They demonstrate a novel aspect of endothelial biomass production which could potentially inhibited to interfere with angiogenesis. The data are clearly presented and the conclusions are clear, the images are of high quality, the manuscript is very well written. However, some major points have to be addressed.

1) SLC25A13 obviously is important for proper EC proliferation. It remained unclear whether this is due to impaired biomass and membrane production or due to cell death. The authors should investigate how SLC25A13 deficiency alters cell cycle progression, apoptosis, necroptosis and necrosis.

2) It is surprising that only silencing of SLC25A13 but not SLC25A12 had such strong effects. Is there gene compensation? Is there upregulation of SLC25A13 upon loss of SLC25A12 and does this rescue the phenotype?

3) Unfortunately, it remained completely unclear which EC express SLC25A13. The authors have access to large databases allowing a detailed bioinformatic expression analysis. This should include information about organ-specific SLC25A13 expression, the type of EC expressing SLC25A13 (e.g. continuous, sinusoidal, etc. but also venous, capillary, arterial).

4) At this stage the medical relevance of this investigation is unclear. The paper would gain importance if the authors could again use in silico analysis to investigate whether the EC expression pattern of SLC25A13 changes in disease conditions e.g. cancer.

5) The authors show convincing data that SLC25A13 is needed for biomass production (including nucleotides) but not for mitochondrial respiration and ATP production (The reviewer suggests to add this information to Figure 5). The reason for this is not convincingly shown. It should at least be better discussed.

6) With reference to point 5), where exactly is SLC25A13 expressed in EC? Is expression limited to the cell membrane or is it (also) part of the mitochondrial membrane?

Referee #2 (Remarks for Author):

In this manuscript, SUBRAMANIAN et al. describe the metabolic function of the glutamate-aspartate antiporter SLC25A13 in angiogenesis. Using EC-GEM, a bionformatic approach previously described by the same group (PMID: 32268117), they found which metabolic pathways are necessary for biomass production in endothelial cells (ECs). Through various bioinformatic considerations, glutamate was revealed to be the most central metabolite, with 27.4% being used for aspartate biosynthesis, a finding already described by another group (PMID: 35580611). From the EC-GEM, the authors predicted SLC25A12 and SLC25A13 to be linked to the mitochondrial export of aspartate. Using silencing approaches in vitro, Subramanian demonstrated that SLC25A13 and not SLC25A12 is crucial to support EC proliferation and migration capacity. They also performed metabolomic analyses in HUVEC to demonstrate that SLC25A13 is critical in coordinating glutamine utilization primarily for cholesterol synthesis and other biomass precursors.
Overall, the work is descriptive in nature and is far to show a critical essential role of SLC25A13 in angiogenesis. Also, the endothelial specificity function (e.g. compared to other tissues) of SLC25A13 is not addressed.

Major issues

The authors identified two potential mitochondrial aspartate-glutamate transporters, SLC25A12 and SLC25A13 by EC-GEM analyses. Can the authors determine the protein levels of both transporters in different endothelial cell lines (HUAEC, HUVEC, CADMEC/HMVEC, etc.) and in other non-vascular cell types where their expression is expected to be high?

How the authors explain the increase in 6-3H thymidine incorporation in SLC25A12KD vs. CTRL (Fig. S2.C)?

The authors analyzed the role of SLC25A13 in a model of laser-induced neovascularization using siRNA tested in vitro in bEnd3 cells. A) Did the authors evaluate the protein levels of SLC25A13 and SLC25A12 in vivo endothelial cells murine (mECs)? B) What happens in a model of physiological angiogenesis, such as retinal angiogenesis, when SLC25A13 is deleted?
These in vivo experiments should be performed with SLCA13 conditional allele Tg animals since with siRNA a non EC autonomous function of SLCA13 can not be excluded.

Considering that the authors observe an increase in the glycolysis (Fig. 4I), endothelial cells (ECs) need to regenerate oxidized NAD+ when mitochondrial function is impaired. The authors also showed a significant increase in the MDH1 enzyme, which converts oxaloacetate (OAA) into malate while oxidizing NADH. Could be interesting analyze the fluxomic of glucose in SLC25A13 vs. CTRL. How did you explain the increase of SLC25A11, the mitochondrial transporter of malate? And, a reduction of SLC25A1 a citrate carrier connected with citrate-malate shuttle?

In the experiments of rescue using an excess of cholesterol, the treatment induced an increase of proliferation of CTRL ECs, because that it is not a functional rescue of the phenotype (Fig. 4D). The link with cholesterol synthesis, although novel, is not supported by the data. Cholesterol supplementation is able to increase proliferation also in wt cells at the same rate as silenced cells, blunting a specific rescue effect on siSLC25A13 compared to controls. Also, cholesterol is not the only mevalonate final products: mevalonate could be used for rescue as well.
Similar comments and conclusion for the effect of dNTP+Glu treatments (Fig. 4E).

How the authors explain metabolically the increase in migration of ECs under SLC25A13 KD?

The graph of NADH/NAD present a lot of dispersion, since it is a key point of the paper it should be improved (Fig. 4J).

The authors stated that RNA seq analyses have been performed on HUVEC silenced for SLCA13 for 6 days. The level of SLCA13 downregulation in these samples is not provided.

Minor

Introduction: Paragraphs 1 and 2 should be combined into one paragraph. And, clarify in the introduction that SLC25A13 is one of the transporters involved in the malate-aspartate shuttle (MAS). The introduction currently lacks an explanation of this metabolic shuttle and the role of AGC and OGC.

Recently, it has been described that aspartate-derived glutamine is crucial for supporting EC proliferation (PMID: 35580611). In fact, it has been shown through 13C fluxomic analysis that glutamine is essential for synthesizing aspartate. The authors should cite and comment previous work carefully when mentioning 'in previous studies in non-ECs...'

Please, add the flux of electrons in Figure 1F considering that the main role MAS is to transport electrons across the mitochondrial membrane, not merely the passage of metabolites.

Please, cancel "Consistently, the reaction associated with mitochondrial to cytosolic aspartate transport ..." In the subsequent sentence, use the abbreviation RKo for the "in-silico reaction knockout" mentioned above.

The authors used ASPLGUm as the abbreviation for mitochondrial aspartate-glutamate transporter. Please use AGC instead.

Fig. 2A, correct the (%.) for (%) in the legend of EdU positive cells graph.

Please, add the scale bar Fig. 2C.

Please, cancel 'which is consistent with the negative enrichment of angiogenesis...' in the last paragraph of the section Results - SCL25A13 is essential for EC proliferation and angiogenesis-, because the analysis of RNAseq is describe in the follow section. Please change 'glutamate-aspartate shuttling' to 'malate-aspartate shuttling.' When referring to the transporter, use 'carrier' or 'transporter'.

Some graphs of 13C-Q fluxomic analysis, including alpha-ketoglutarate (M+5) (Fig. 3E) and aspartate (M+4)(Fig. 3F) levels are significant when CTRL vs. SLC25A12KD are compared, please can you check this information.

In the discussion, in the second paragraph please correct this sentence: 'It has been documented that different' by 'It has been documented two different...'

In the discussion, in the fourth paragraph the authors suggest a shift of glutamine oxidative phenotype, but the results did not indicate and increase of oxidative glutaminolysis (fluxomics and decrease of GLS). So, remove the sentence.

M&M section: it is not clear what this statement means: "All genes that were expressed in at least 6 of the total 12 samples .... were retained for further analysis". Es: Could be that 5 samples have been used as controls on 1 sample in the KD conditions? Please explain precisely how and which samples of ctrl and KD have been used for transcriptomics analyses.

Referee #3 (Comments on Novelty/Model System for Author):

Minor comments are in the remarks to the author.

Referee #3 (Remarks for Author):

The manuscript is logical and well written. Beyond further highlighting the importance of cell metabolism for energy and biomass production, the manuscript is a nice application of "modeling" to guide efficient experiments.

This reviewer would offer a few suggestions for consideration by the authors.

1) It seems the modeling led to the identification of two transporters. The silencing of one resulted in the finding and the silencing of the other had no effect. Can the inverse of this be established, specifically can the modeling identify a point that is not predicted to have an effect and can the negative predicitve value of the modeling be established. This would not require the entire battery of experiments, merely enough to extend the validation utility of the modeling.

2) Is data available on the extent of modulation of the target that was required for the experimental read-outs to be appreciated? Perhaps this was in the modeling and could be emphasized. It could be fair to say that metabolism is a "tightly-regulated" process. Understanding the extent of variation in target expression/activity is needed would be helpful in the

generalizability/applicability of the (very interesting) findings.

3. The purely in-vitro parts of the study were in one EC type. As from the above, there would be no need to repeat the entire study in another cell line (or in male vs. female) but some data confirming that findings are not restricted to HUVEC would be ideal.

4) Assuming that the modeling and thus the experiments that emerged from the modeling were not done across experimental conditions (i.e. states of high glucose or hypoxa) perhps this could be added to the limitations.

Referee #1 (Remarks for Author):

This manuscript reports for the first time about a role of the glutamate-aspartate antiporter SLC25A13 in endothelial cells. The Carmeliet group used their previously described large data sets of endothelial gene expression and bioinformatic tools to predict genes controlling endothelial cell metabolism including SLC25A13. There are no publications in PubMed reporting about this gene in endothelial cells. The authors studied this gene using shRNAs to inhibit its expression in primary endothelial cell cultures. In addition, they treated the mouse retina (AMD model) with such constructs to achieve a gene knockdown, however not exclusively in endothelial cells. The experiments revealed that this glutamate transporter plays important roles for biomass production including cholesterol, a finding which was not reported before in other cell types. A thorough state of the art investigation of cellular metabolism confirmed this and suggested the biochemic mechanism. In addition, silencing of SLC25A13 impaired endothelial proliferation, migration and tube formation. As such the data are very interesting. They demonstrate a novel aspect of endothelial biomass production which could potentially inhibited to interfere with angiogenesis. The data are clearly presented and the conclusions are clear, the images are of high quality, the manuscript is very well written. However, some major points have to be addressed.

Response: We thank the reviewer for their positive evaluation of our work. We have conducted additional experiments to address the questions raised. Figures for reviewers as response to their questions are indicated by Fig. RL. We indicated when these data were included in the revised figures.

1) SLC25A13 obviously is important for proper EC proliferation. It remained unclear whether this is due to impaired biomass and membrane production or due to cell death. The authors should investigate how SLC25A13 deficiency alters cell cycle progression, apoptosis, necroptosis and necrosis

Response: We thank the reviewer for their comment and agree that a deeper analysis of the cell cycle and cell death would provide a more comprehensive understanding of whether the change in EC proliferation is due to impaired biomass production or increased cell death. To address this, we examined six HUVEC donors using Annexin V and PI staining kit by FACS (Fig. RL1A-B). An increase in apoptosis (%) (gated as PI- Annexin AF488+ population) was observed

24 hours following SLC25A13 silencing, with no corresponding changes in necrosis (gated as PI+ Annexin AF488+ population). To investigate the potential induction of necroptosis, necrostatin was used as an inhibitor. However, no significant changes in necroptosis were detected upon SLC25A13 silencing (Fig. RL1C). Despite the observed increase in apoptosis, the reduction in proliferation was more pronounced, with a 48% decrease, which is also reflected in the cell cycle profile (Fig. RL1A-B). Silencing of SLC25A13 led to S-phase arrest, indicating alterations in the cell cycle. Biomass impairment has been associated with nucleotide deprivation, which can cause cells to arrest in the S phase, accompanied by a decrease in the G2/M phase (Fig. RL1D-E).

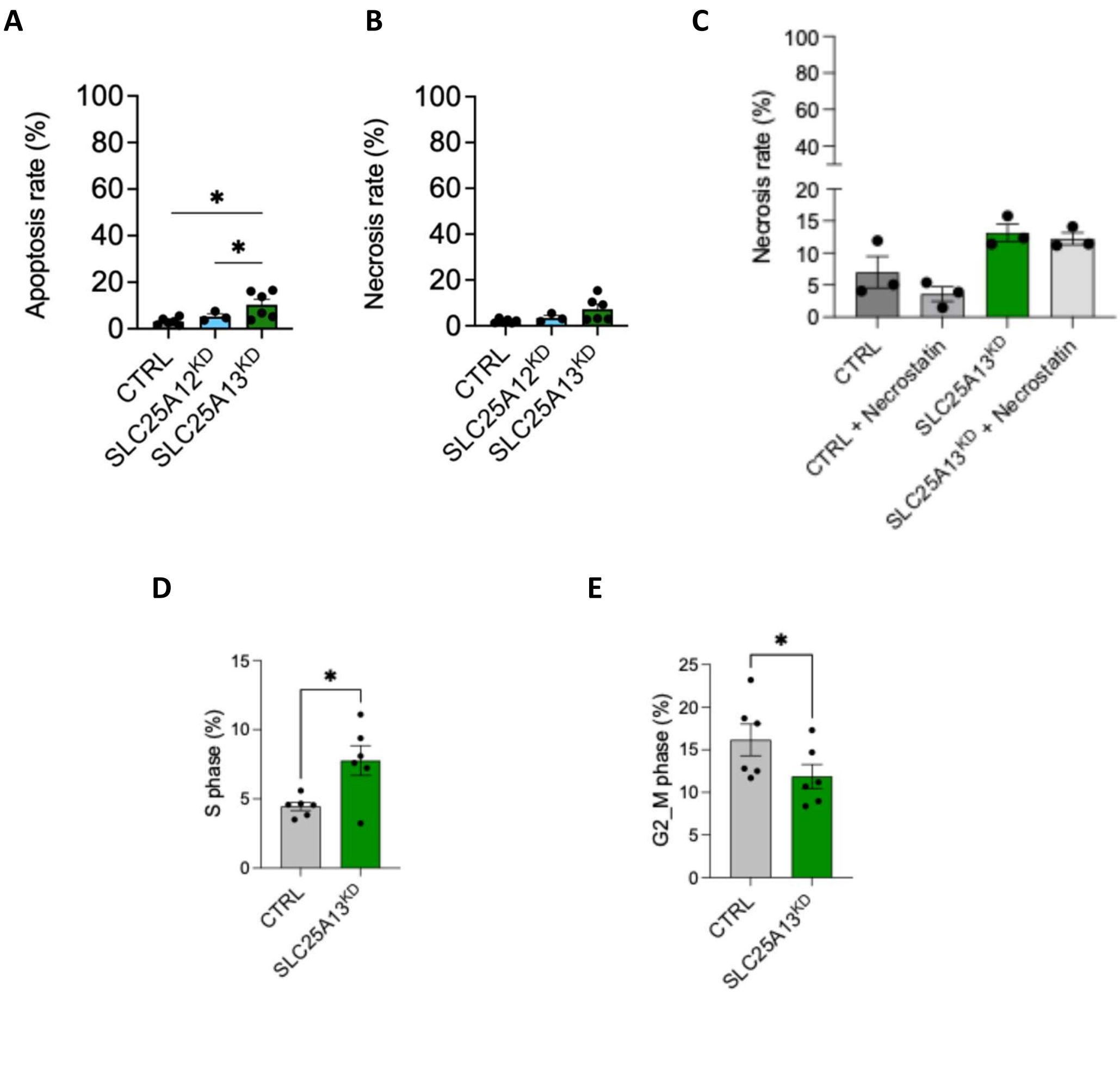

Figure RL1: A. Quantification of apoptosis with Annexin/PI test. I. B. Quantification of necrosis with Annexin/PI test after treatment with Necrostatin-1. C. Quantification of necroptosis with Annexin/PI test. D. Quantification of cell cycle S-phase E. Quantification of cell cycle G2_M-

phase Data are means ± SEM; *p<0.05, **p<0.01 by one sample t-test and Wilcoxon test (A, B); *p<0.05, **p<0.01, ****p<0.0001 by a t-test (C); by a two-tailed paired t-test (C, D, E, F); by a one-way ANOVA (C).

(**Figure RL1 has been included as Figs. EV2F-J in the revised manuscript)**

2) It is surprising that only silencing of SLC25A13 but not SLC25A12 had such strong effects. Is there gene compensation? Is there upregulation of SLC25A13 upon loss of SLC25A12 and does this rescue the phenotype?

Response: We understand the reviewer's concern regarding the disparity in the impact of silencing SLC25A13 and SLC25A12, given their documented roles in the malate-aspartate shuttle - a pathway that facilitates the transport of metabolites between cellular compartments. qPCR analysis confirmed that silencing SLC25A12 does not lead to a compensatory upregulation of SLC25A13 (Fig. RL2A-B). Notably, SLC25A13 is expressed across tissues involved in amino acid metabolism and energy production like the liver, kidney (Fig. RL2C). In contrast, SLC25A12 is predominantly expressed in the brain, where it plays roles in neurodevelopment and neurodegeneration, although it is also present in other tissues like tongue, stomach, colon, heart muscle, skeletal muscle (Fig. RL2D). In HUVECs, basal expression of SLC25A12 is lower than that of SLC25A13. We hypothesize that these differences in tissue distribution and expression levels might contribute to the divergent outcomes observed upon silencing each gene.

A

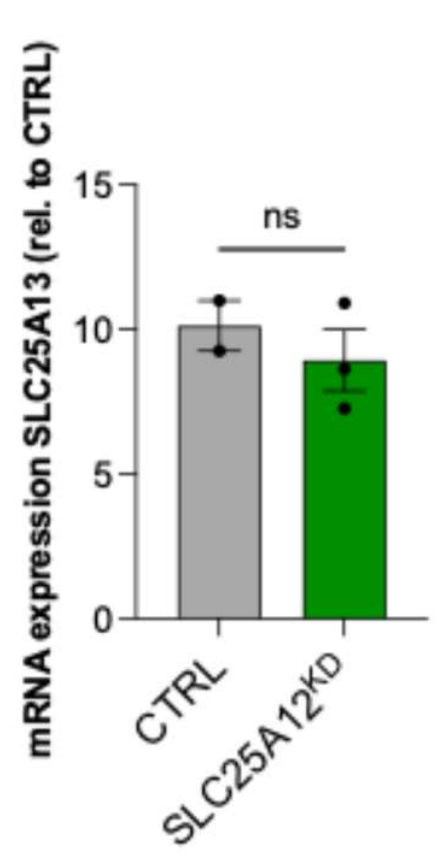

B

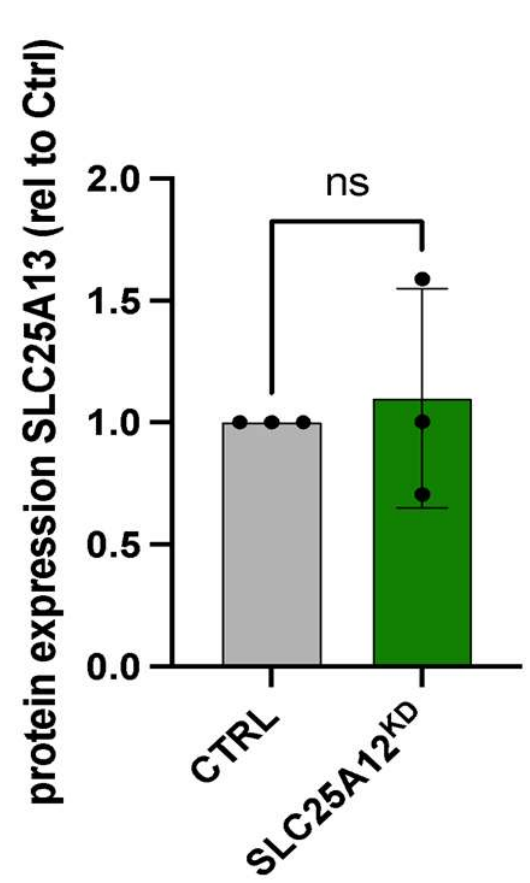

C. SLC25A13 expression

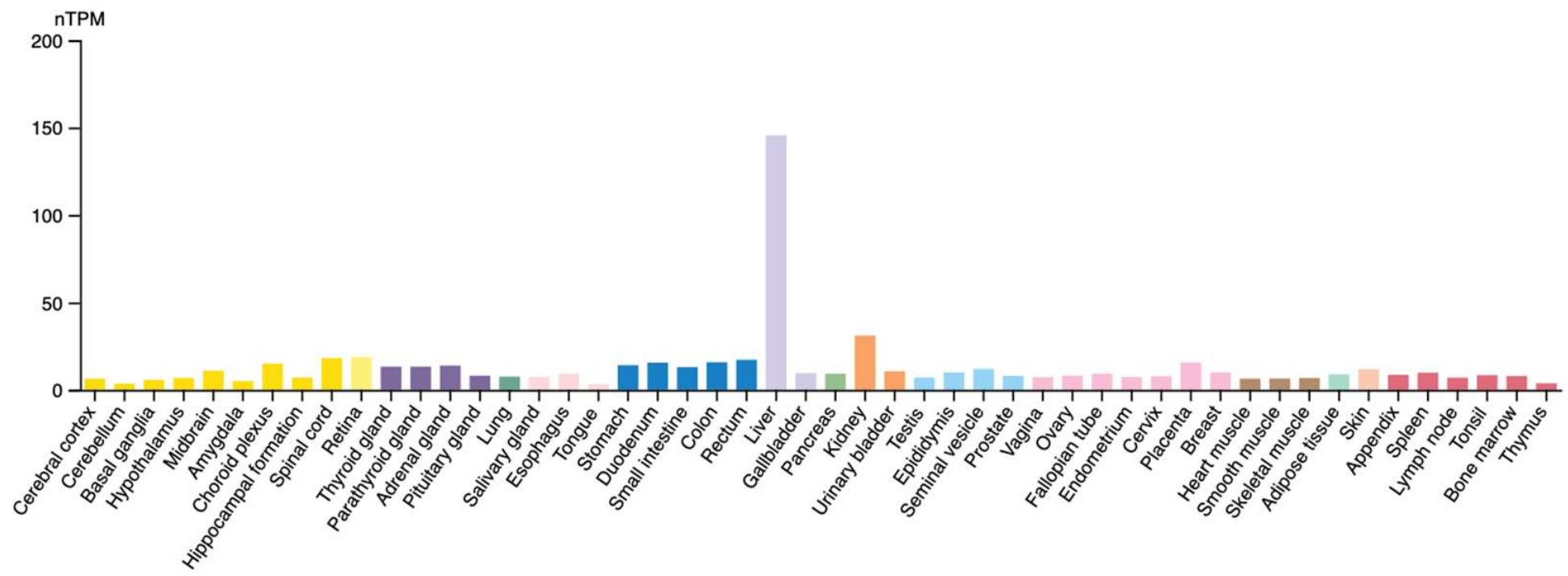

D. SLC25A12 expression

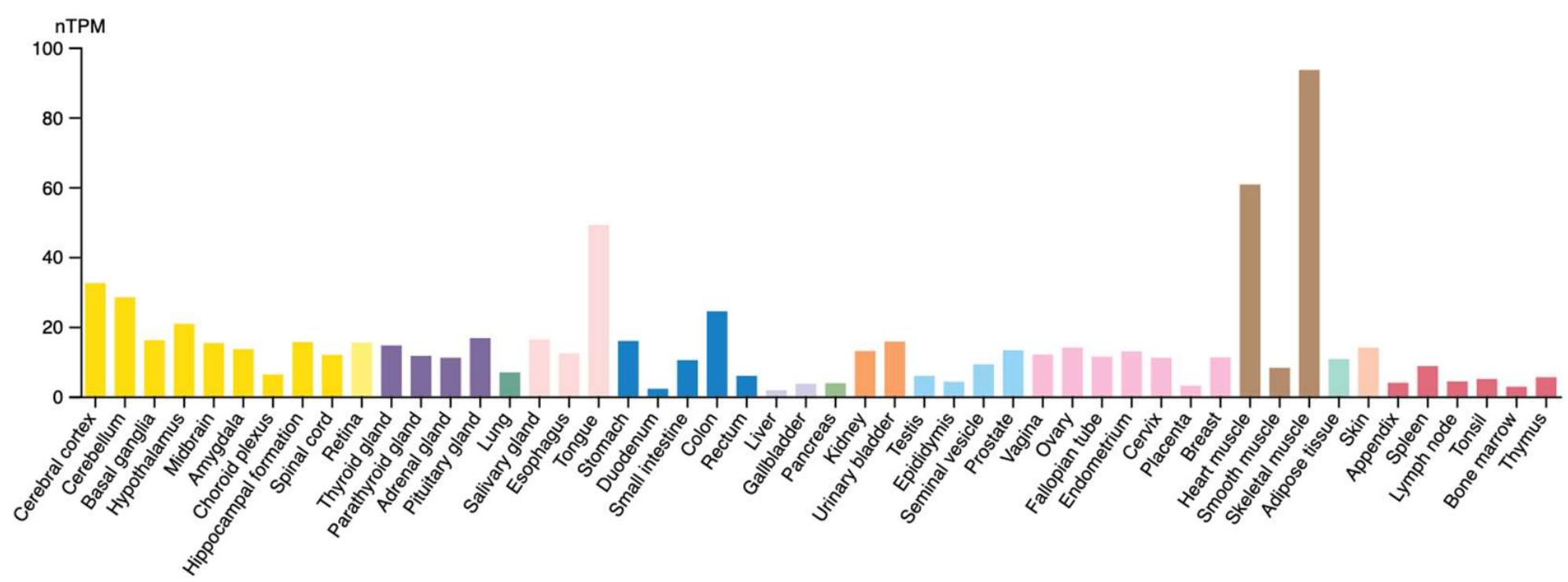

Figure RL2: A. Quantification of SLC25A13 mRNA expression levels in CTRL and SLC25A12KD ECs (expressed relative to control) (n=2-3). B. Densitometric quantification of SLC25A13 protein levels in CTRL and SLC25A12KD ECs (expressed relative to control) (n=3). C. SLC25A13 expression in various human tissues (Consensus dataset, Human Protein Atlas). D. SLC25A12 expression in various human tissues (Consensus dataset, Human Protein Atlas).

(**Figs. RL2 A and B are included as Figs. EV2D-2E in the revised manuscript)**

3) Unfortunately, it remained completely unclear which EC express SLC25A13. The authors have access to large databases allowing a detailed bioinformatic expression analysis. This should include information about organ-specific SLC25A13 expression, the type of EC expressing SLC25A13 (e.g. continuous, sinusoidal, etc. but also venous, capillary, arterial).

Response: We thank the reviewer for their suggestion and performed a detailed bioinformatic expression analysis. As mentioned in the previous question, SLC25A12 and SLC25A13 are expressed in distinct tissues. However, we analyzed large-scale single-cell transcriptomic datasets focusing on endothelial cells (ECs) and found no evidence of selective SLC25A13

expression in specific EC subpopulations. In particular, the number of ECs within the specific subpopulations (e.g. proliferative ECs) is too low to allow meaningful comparisons between tumor and adjacent regions, preventing any definitive conclusions about specific EC subtypes. However, we do observe that SLC25A13 displays a homogeneous expression pattern across different ECs (Fig. RL3), and its expression is also detected in various other cell types beyond ECs. Overall, SLC25A13 seems to show a higher expression in vascular and non-vascular cell lines as compared to SLC25A12.

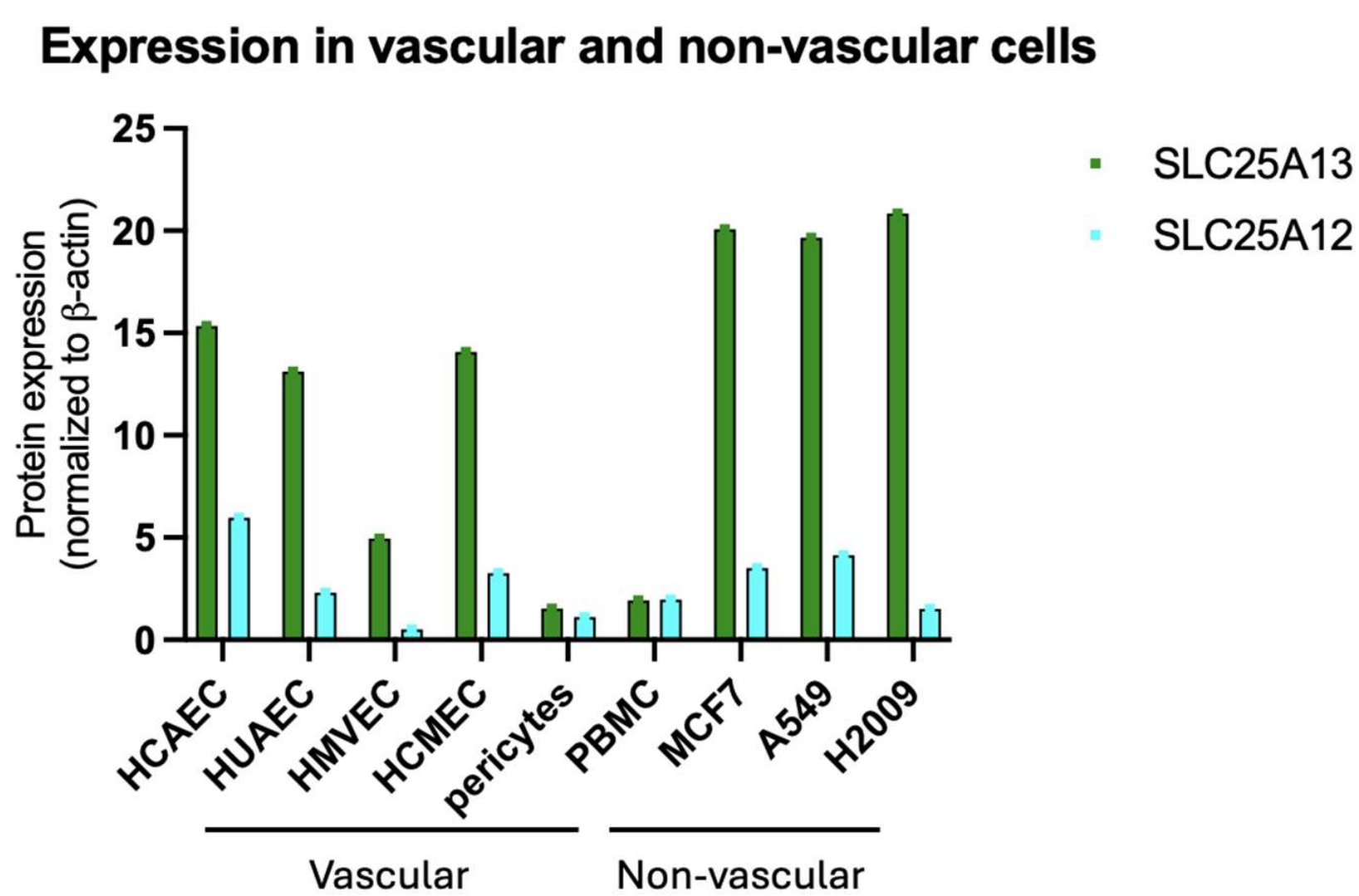

Figure RL3: SLC25A13 and SLC25A12 protein expression normalized to β - actin in vascular and non-vascular cells.

(Figure RL3 is included as Fig. EV5C in the revised manuscript)

4) At this stage the medical relevance of this investigation is unclear. The paper would gain importance if the authors could again use in silico analysis to investigate whether the EC expression pattern of SLC25A13 changes in disease conditions e.g. cancer.

Response: We greatly appreciate this suggestion. An *in silico* analysis was conducted to examine the expression of SLC25A13 in ECs across multiple datasets, including those related to inflammatory diseases (Fig. RL4A) and cancer (Fig. RL4B). The results revealed an upregulation of EC related SLC25A13 expression in certain inflammatory conditions, including COVID-19, as well as a significant increase in various cancer subtypes, such as lung, kidney, and intestinal cancers. Moreover, analysis of a lung cancer dataset (Lambrechts *et al*, 2018)

indicated that SLC25A13 expression is more pronounced in ECs in cancer conditions as compared to other cell types (Fig. RL4C). These findings suggest that SLC25A13 may play a key role in supplying metabolites for biomass synthesis to support the proliferation of tumor-associated ECs.

SLC25A13 expression in ECs across multiple datasets:

A

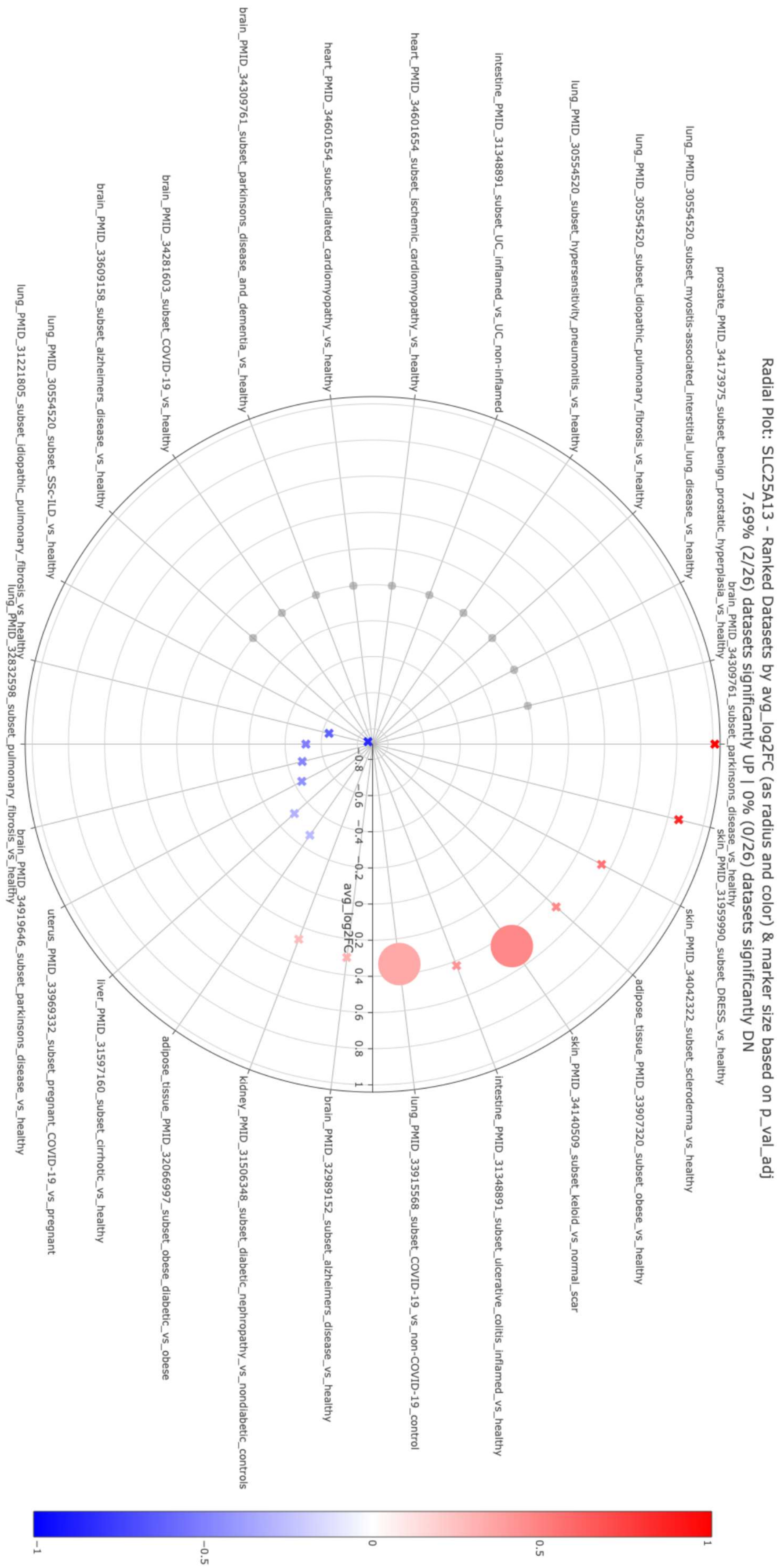

Radial Plot: SLC25A13 - Ranked Datasets by avg_log2FC (as radius and color) & marker size based on p_val_adj
7.69% (2/26) datasets significantly UP | 0% (0/26) datasets significantly DN
avg_log2FC
prostate_PMID_34173975_subset_benign_prostatic_hyperplasia_vs_healthy
lung_PMID_30554520_subset_myositis-associated_interstitial_lung_disease_vs_healthy
lung_PMID_30554520_subset_idiopathic_pulmonary_fibrosis_vs_healthy
lung_PMID_30554520_subset_hypersensitivity_pneumonitis_vs_healthy
intestine_PMID_31348891_subset_UC_inflamed_vs_UC_non-inflamed
heart_PMID_34601654_subset_ischemic_cardiomyopathy_vs_healthy
heart_PMID_34601654_subset_dilated_cardiomyopathy_vs_healthy
brain_PMID_34309761_subset_parkinsons_disease_and_dementia_vs_healthy
brain_PMID_34281603_subset_COVID-19_vs_healthy
brain_PMID_33609158_subset_alzheimers_disease_vs_healthy
lung_PMID_30554520_subset_SSc-ILD_vs_healthy
lung_PMID_31221805_subset_idiopathic_pulmonary_fibrosis_vs_healthy
lung_PMID_32832598_subset_pulmonary_fibrosis_vs_healthy
brain_PMID_34919646_subset_parkinsons_disease_vs_healthy
uterus_PMID_33969332_subset_pregnant_COVID-19_vs_pregnant
liver_PMID_31597160_subset_cirrhotic_vs_healthy
adipose_tissue_PMID_32066997_subset_obese_diabetic_vs_obese
kidney_PMID_31506348_subset_diabetic_nephropathy_vs_nondiabetic_controls
brain_PMID_32989152_subset_alzheimers_disease_vs_healthy
lung_PMID_33915568_subset_COVID-19_vs_non-COVID-19_control
intestine_PMID_31348891_subset_ulcerative_colitis_inflamed_vs_healthy
skin_PMID_34140509_subset_keloid_vs_normal_scar
adipose_tissue_PMID_33907320_subset_obese_vs_healthy
skin_PMID_34042322_subset_scleroderma_vs_healthy
skin_PMID_31959990_subset_DRESS_vs_healthy
brain_PMID_34309761_subset_parkinsons_disease_vs_healthy
−0.8 −0.6 −0.4 −0.2 0 0.2 0.4 0.6 0.8 1
−1 −0.5 0 0.5 1

B

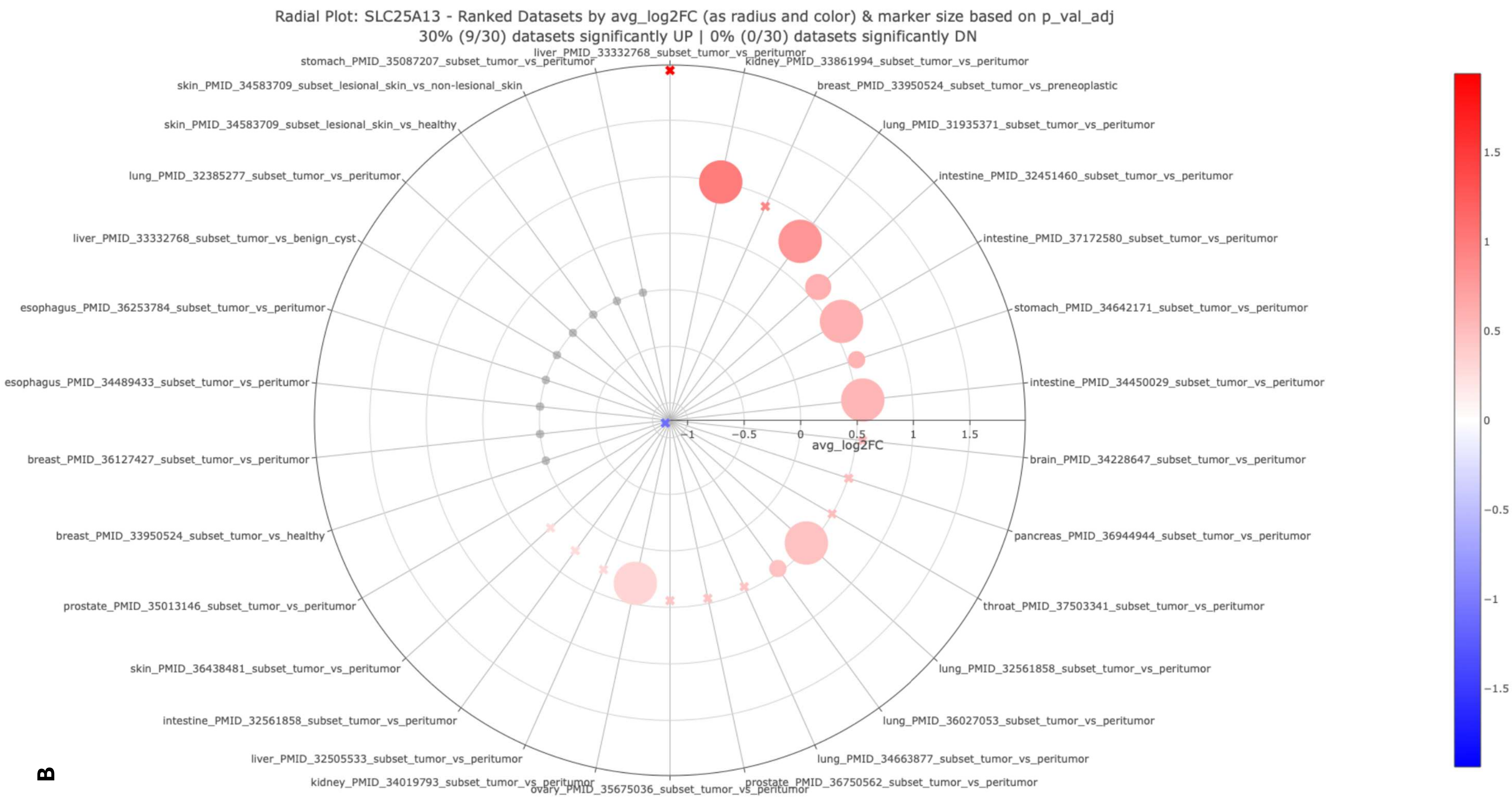

Radial Plot: SLC25A13 - Ranked Datasets by avg_log2FC (as radius and color) & marker size based on p_val_adj
30% (9/30) datasets significantly UP | 0% (0/30) datasets significantly DN
liver_PMID_33332768_subset_tumor_vs_peritumor
kidney_PMID_33861994_subset_tumor_vs_peritumor
breast_PMID_33950524_subset_tumor_vs_preneoplastic
lung_PMID_31935371_subset_tumor_vs_peritumor
intestine_PMID_32451460_subset_tumor_vs_peritumor
intestine_PMID_37172580_subset_tumor_vs_peritumor
stomach_PMID_34642171_subset_tumor_vs_peritumor
intestine_PMID_34450029_subset_tumor_vs_peritumor
brain_PMID_34228647_subset_tumor_vs_peritumor
pancreas_PMID_36944944_subset_tumor_vs_peritumor
throat_PMID_37503341_subset_tumor_vs_peritumor
lung_PMID_32561858_subset_tumor_vs_peritumor
lung_PMID_36027053_subset_tumor_vs_peritumor
lung_PMID_34663877_subset_tumor_vs_peritumor
prostate_PMID_36750562_subset_tumor_vs_peritumor
ovary_PMID_35675036_subset_tumor_vs_peritumor
kidney_PMID_34019793_subset_tumor_vs_peritumor
liver_PMID_32505533_subset_tumor_vs_peritumor
intestine_PMID_32561858_subset_tumor_vs_peritumor
skin_PMID_36438481_subset_tumor_vs_peritumor
prostate_PMID_35013146_subset_tumor_vs_peritumor
breast_PMID_33950524_subset_tumor_vs_healthy
breast_PMID_36127427_subset_tumor_vs_peritumor
esophagus_PMID_34489433_subset_tumor_vs_peritumor
esophagus_PMID_36253784_subset_tumor_vs_peritumor
liver_PMID_33332768_subset_tumor_vs_benign_cyst
lung_PMID_32385277_subset_tumor_vs_peritumor
skin_PMID_34583709_subset_lesional_skin_vs_healthy
skin_PMID_34583709_subset_lesional_skin_vs_non-lesional_skin
stomach_PMID_35087207_subset_tumor_vs_peritumor
−1
−0.5
0
0.5
1
1.5
avg_log2FC
1.5
1
0.5
0
−0.5
−1
−1.5

C **Expression of SLC25A13 – ECs vs. Non-ECs**

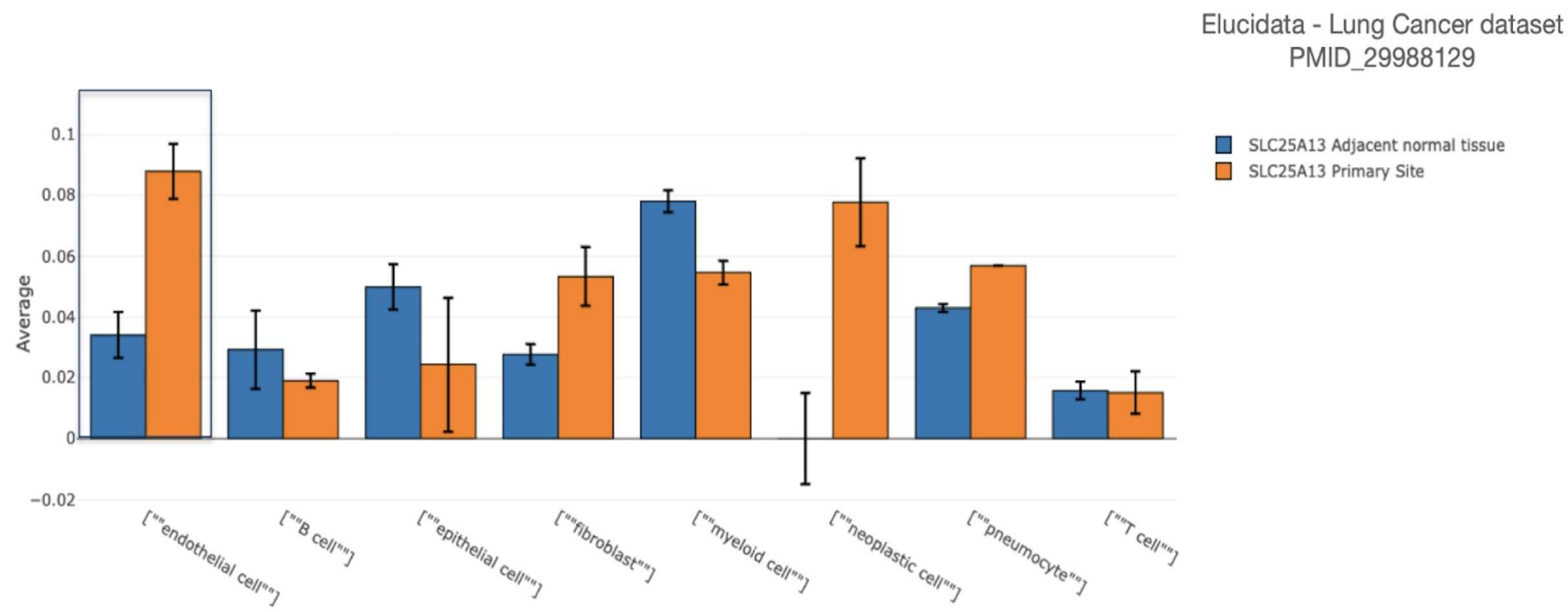

Figure RL4: A. Radial plot of SLC25A13 expression in inflammatory datasets. B. Radial plot of SLC25A13 expression in tumor datasets. C. Comparison of SLC25A13 expression between tumor (primary site) and peritumoral (adjacent normal tissue) regions across cell types in ECs and other non-vascular cell types in a lung cancer dataset

(Figures RL4A and RL4B are included as Figs. EV5A-B in the revised manuscript)

This text referred to a figure for reviewers that was removed because it displayed unpublished data. The text also referred to this data and was consequently removed.

Figure for reviewers removed

5) The authors show convincing data that SLC25A13 is needed for biomass production (including nucleotides) but not for mitochondrial respiration and ATP production (The

reviewer suggests to add this information to Figure 5). The reason for this is not convincingly shown. It should at least be better discussed.

Response: We acknowledge the reviewer's concern that perturbing SLC25A13 might lead to a change in redox balance and affect mitochondrial respiration and ATP production. Contrary to the expectations, we observed no significant difference either in the glucose-labelled carbon fraction of glucose-6-phosphate as measured through ^{13}C glucose-based isotope tracing or glycolytic flux measured from the glycolytic flux assay (Fig. RL6A, Fig. 4N). In other words, like healthy ECs, SLC25A13^{KD} ECs probably retain their ability to sustain ATP synthesis through glycolysis (Fig. 4L, M of the main manuscript).

Similarly, no significant alterations were captured in ^{13}C-glucose carbon labelled TCA cycle intermediates (pyruvate, citrate and malate) (Fig. RL6B). However, as shown in the previous draft, our ^{13}C glutamine tracing results revealed significant reduction in both oxidation and reductive carboxylation of glutamine to succinate and significant reduction in the reductive carboxylation of glutamine to aspartate (Fig. 3F,G of the main manuscript). Overall, these data show that oxidative glucose metabolism remains unaltered with the SLC25A13^{KD}, preferentially altering glutamine catabolism.

Also, no significant change in the NADH/NAD^+ ratio was observed (Fig. EV4E). Similarly, CM-H_2DCFDA fluorescence – a proxy for ROS levels - remains unchanged following SLC25A13 silencing. This probably suggests a compensatory role of glucose oxidation in sustaining redox balance in perturbed ECs. This finding suggests that maintaining overall redox balance is not the primary function of SLC25A13.

Rescue experiments using mevalonate, a precursor of cholesterol, and unchanged glucose-derived cholesterol levels establish SLC25A13's role in selectively mediating glutamine catabolism-linked mevalonate synthesis (Fig. RL6D). Taken together, these data point out that biomass synthesis is the main function of SLC25A13 in ECs and it selectively mediates glutamine catabolism for sustaining mevalonate / cholesterol biosynthesis.

Based on the above observations, the Discussion section in the revised manuscript has been adapted. Accordingly, Figure RL7, Figure 5 in the main manuscript has also been revised.

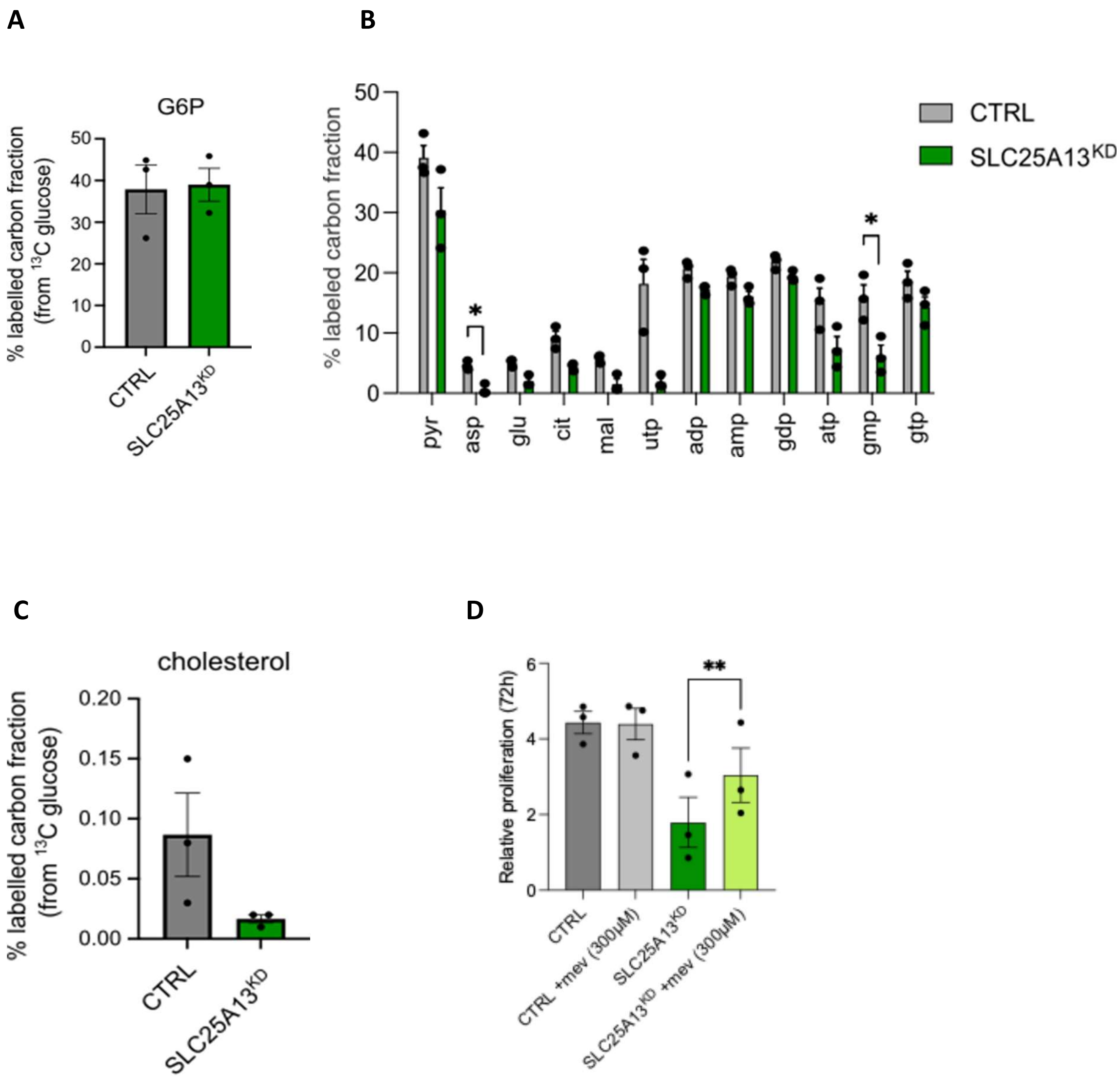

Figure RL6: A. Carbon fraction of glucose-6-phosphate (G6P) labelled from U-13C glucose (n=3). B. Bar plots representing the total fraction of [U-13C]-glucose carbon in pyruvate (pyr), amino acids (asp, glu), TCA metabolites (cit, mal), nucleotides (ump, adp, amp, gdp, atp, gmp, gtp) and cholesterol precursor (mev) (n=3). C. Carbon fraction of cholesterol labelled from U-13C glucose (n=3). D. Rescue with excess Mevalonate: Barplots demonstrating the comparison between CTRL and SLC25A13KD ECs grown in EGM2 + 300µM Mevalonate after 72h (n=3). Data are means ± SEM; **p<0.01 by one-way ANOVA. adp: adenosine diphosphate, atp: adenosine triphosphate, amp: adenosine monophosphate, asp: aspartate, cit: citrate, gdp: guanosine diphosphate, glu: glutamate, gmp: guanosine monophosphate, mal: malate, pyr: pyruvate, utp: uridine triphosphate. Data are means ± SEM; not significant, by a two-tailed

paired t-test. Rescue with excess Mevalonate: Barplots demonstrating the comparison between CTRL and SLC25A13KD ECs grown in EGM2 + 300µM Mevalonate after 72h (n=3). Data are means ± SEM; **p<0.01 by one-way ANOVA. **(Figs. RL6A - C is implemented as Figs. EV3C - 3E respectively. Fig. RL6D is included as Fig. 4E in the revised manuscript)**

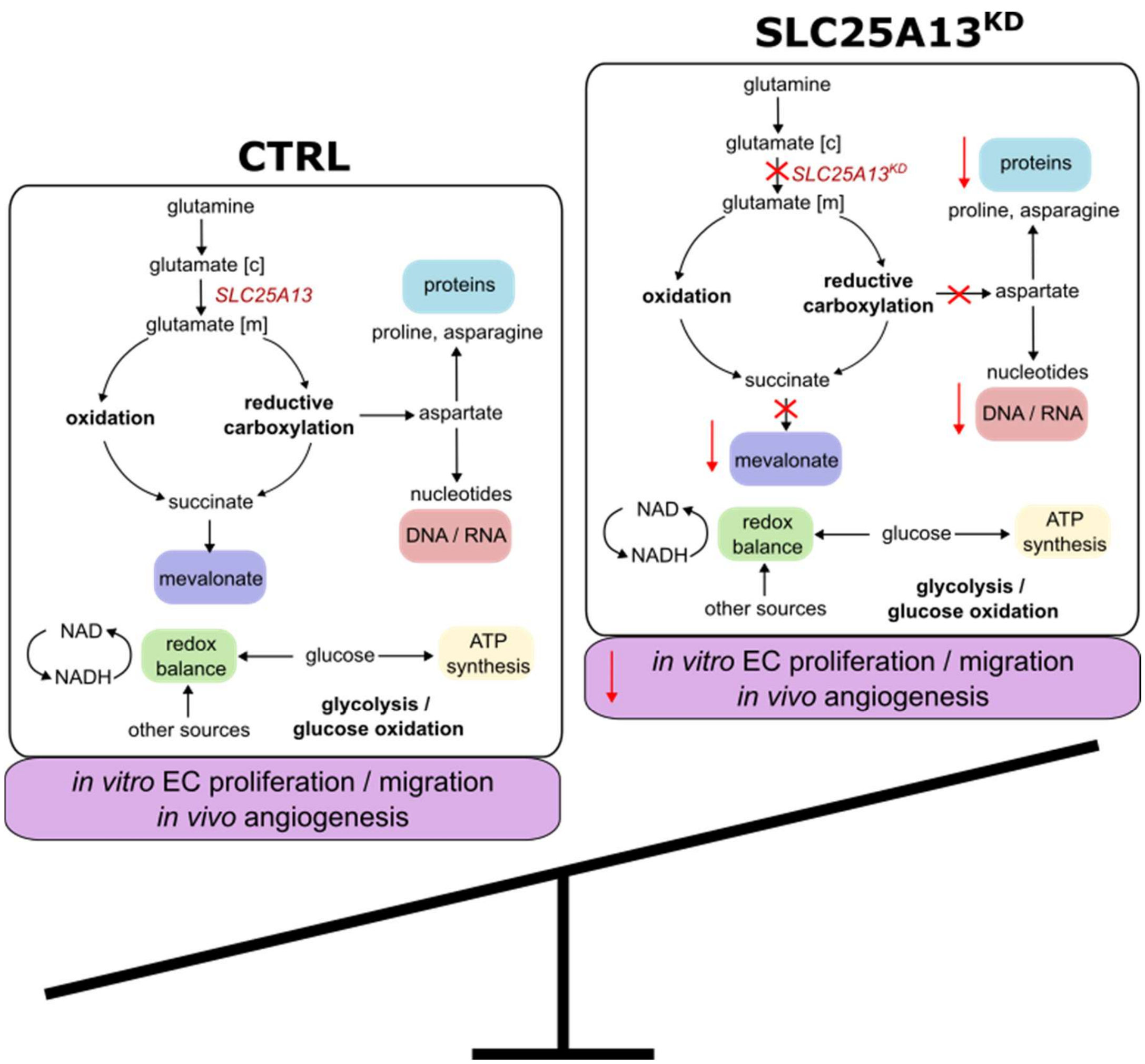

Figure RL7: Working model for explaining the metabolic role of SLC25A13KD in ECs. A scheme representing the metabolic role of SLC25A13KD vs. control in ECs. **(Figure RL7 is included as Fig. 5 in the revised manuscript).**

6) With reference to point 5), where exactly is SLC25A13 expressed in EC? Is expression limited to the cell membrane or is it (also) part of the mitochondrial membrane?

Response: We thank the reviewer for this important point. In endothelial cells, SLC25A13 is not localized to the plasma membrane but to mitochondria. In our experiments using human lung microvascular endothelial cells (HMVEC-L), we confirmed its mitochondrial localization,

with expression detected at the mitochondrial membrane (Fig. RL8A). Functionally, SLC25A13 mediates the exchange of aspartate (from mitochondria to the cytosol) and glutamate + H^+ (from the cytosol to mitochondria).

This observation is consistent with previous reports and database annotations in other cell types. SLC25A13 is a well-characterized mitochondrial carrier protein localized to the inner mitochondrial membrane (Palmieri, 2004; Palmieri et al., 2001; Human Protein Atlas) (Fig. RL8B).

A **B**

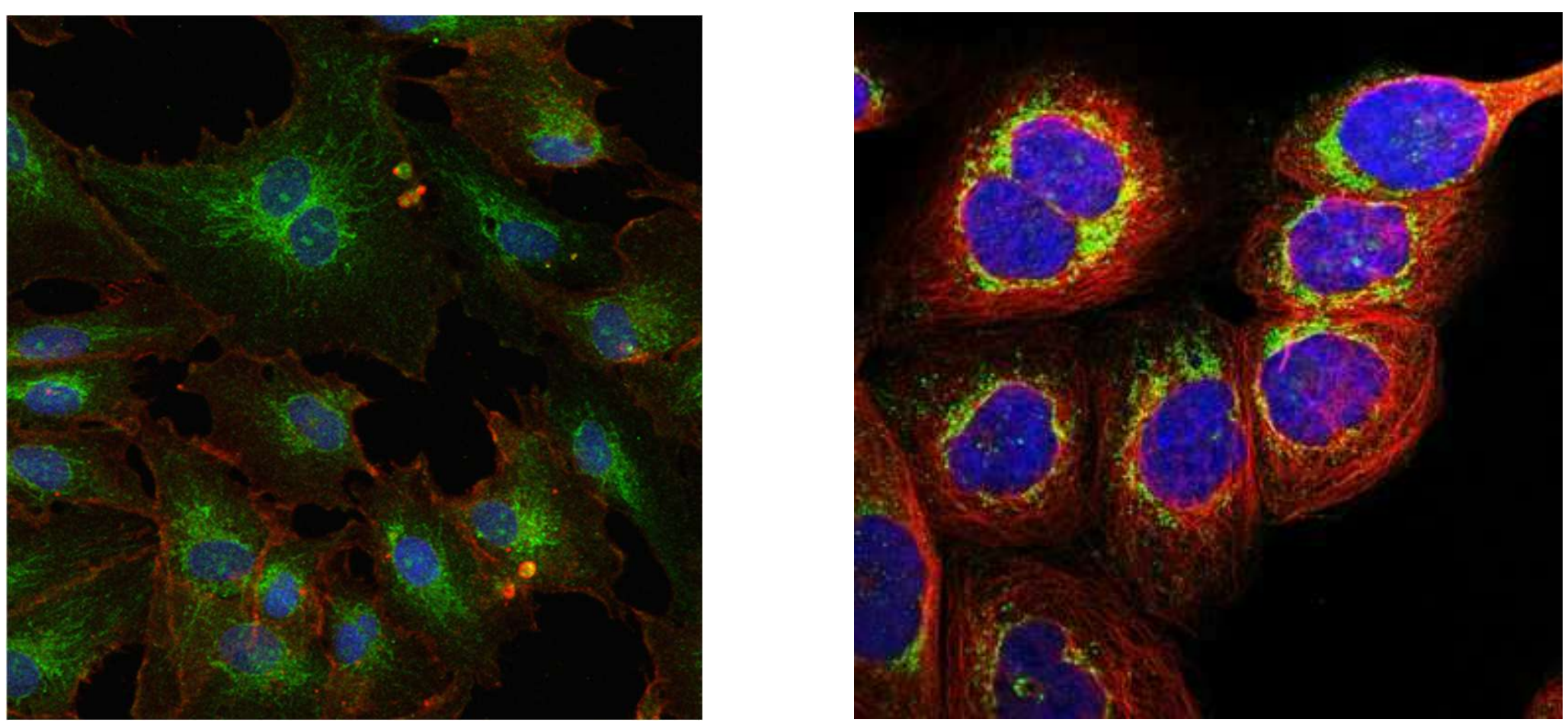

Fig. RL8. A. Representative SLC25A13 staining of HMVEC-L using ab96303 antibody (Abcam). Nucleus (Blue), SLC15A13 (green), CD31 (red) B. Representative SLC25A13 staining of A-431 cell line using HPA018997 antibody (Sigma-Aldrich). Nucleus (Blue), SLC15A13 (green), microtubules (red) (Source: Human Protein Atlas).

Referee #2 (Remarks for Author):

In this manuscript, SUBRAMANIAN et al. describe the metabolic function of the glutamate-aspartate antiporter SLC25A13 in angiogenesis. Using EC-GEM, a bioinformatic approach previously described by the same group (PMID: 32268117), they found which metabolic pathways are necessary for biomass production in endothelial cells (ECs). Through various bioinformatic considerations, glutamate was revealed to be the most central metabolite, with 27.4% being used for aspartate biosynthesis, a finding already described by another group (PMID: 35580611). From the EC-GEM, the authors predicted SLC25A12 and SLC25A13 to be linked to the mitochondrial export of aspartate. Using silencing approaches in vitro, Subramanian demonstrated that SLC25A13 and not SLC25A12 is crucial to support EC proliferation and migration capacity. They also performed metabolomic analyses in HUVEC to demonstrate that SLC25A13 is critical in coordinating glutamine utilization primarily for cholesterol synthesis and other biomass precursors. Overall, the work is descriptive in nature and is far to show a critical essential role of SLC25A13 in angiogenesis. Also, the endothelial specificity function (e.g. compared to other tissues) of SLC25A13 is not addressed.

Major issues

1) The authors identified two potential mitochondrial aspartate-glutamate transporters, SLC25A12 and SLC25A13 by EC-GEM analyses. Can the authors determine the protein levels of both transporters in different endothelial cell lines (HUAEC, HUVEC, CADMEC/HMVEC, etc.) and in other non-vascular cell types where their expression is expected to be high?

Response: As requested by the reviewer, the protein expression of both SLC25A12 and SLC25A13 was verified in several other vascular and non-vascular cell types by Western Blot (Fig. RL3). As shown in the figure, SLC25A13 expression is homogeneous, with the protein detected across all analyzed cell lines. Expression has been confirmed in various EC types, including aortic, coronary artery, and lung microvascular endothelial cells. Additionally, SLC25A13 is expressed in immune cells such as peripheral blood mononuclear cells (PBMCs), as well as in tumor-derived cell lines from breast and lung cancers, including MCF7, A549, and H2009 (Wu *et al*, 2025). These findings are consistent with existing literature and the Human Protein Atlas, which reports widespread expression of SLC25A13 across diverse tissues (please

see answer to Questions 2 & 3 of Reviewer 1). A comparative analysis of SLC25A13 and SLC25A12 expression levels shows that SLC25A12 is expressed at significantly lower levels in all examined cell types. This may explain the more subtle effects observed upon SLC25A12 silencing, as SLC25A13 likely compensates and maintains its functional activity.

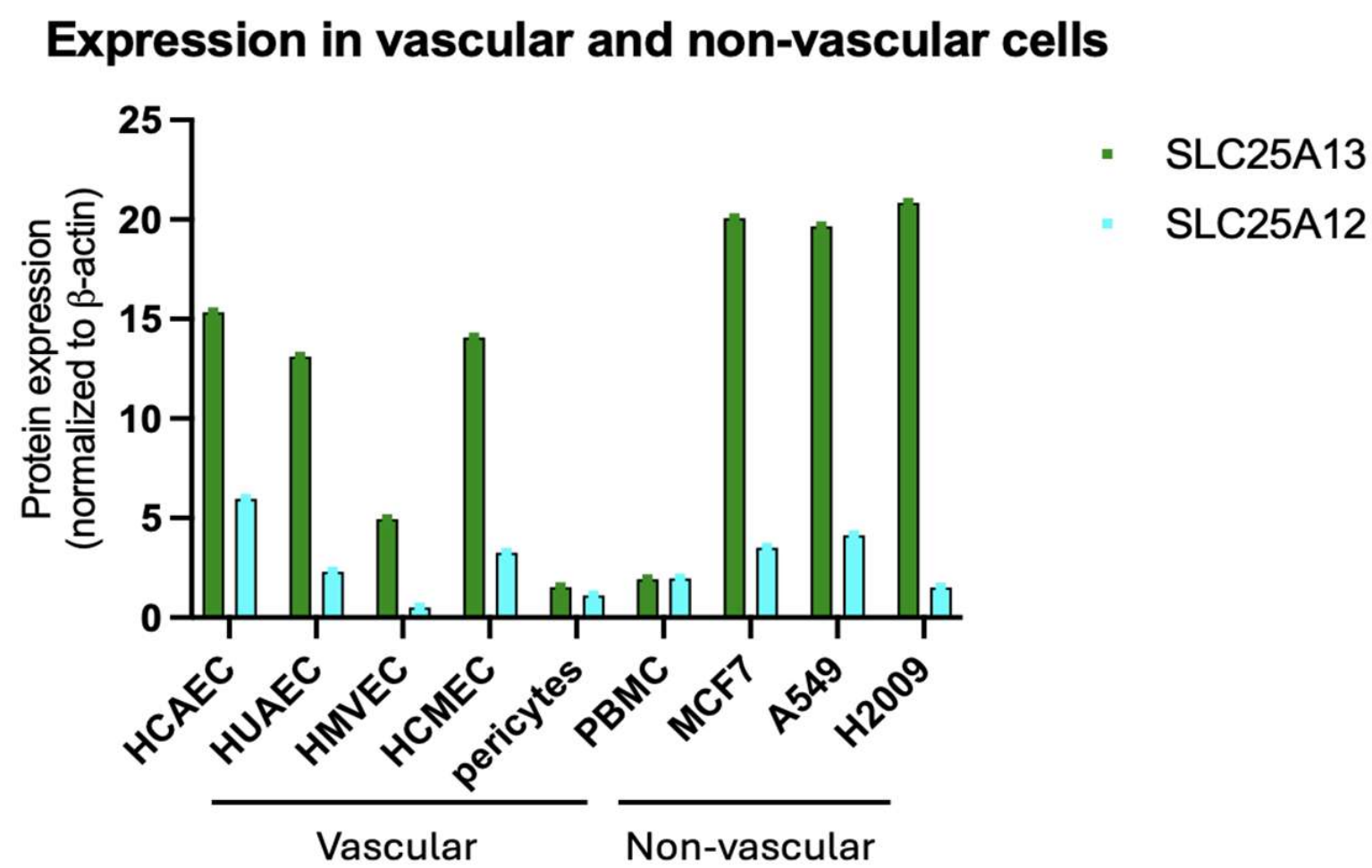

Figure RL3: SLC25A13 and SLC25A12 protein expression normalized to β - actin in vascular and non-vascular cells. **(Figure RL3 is included as Fig. EV5C in the revised manuscript)**

2) How the authors explain the increase in 6-3H thymidine incorporation in SLC25A12KD vs. CTRL (Fig. EV2.C)?

Response: Although SLC25A12 and SLC25A13 share equivalent substrate specificity, previous studies highlighted distinct features of each isoform under both physiological and pathological conditions (Gutierrez-Aguilar & Baines, 2013). Despite these insights, little is known about the regulatory mechanisms governing SLC25A12 and SLC25A13 gene expression or the factors responsible for their differential expression across tissues. Both genes are paralogs within the mitochondrial carrier family, and while they facilitate the exchange of aspartate and glutamate across the mitochondrial inner membrane, several studies demonstrated that — despite their co-localization — SLC25A12 and SLC25A13 fulfil non-redundant functions (Discussion section in the revised manuscript).

Importantly, cell proliferation appears to be dependent on the presence of one isoform or the other. In our study, knockdown of SLC25A12 resulted in increased proliferation, as confirmed by thymidine incorporation assays, consistent with our prior findings (Figure RL9A,

RL9B). However, when we performed a double knockdown ($SLC25A12^{KD}$ and $SLC25A13^{KD}$) and compared it to $SLC25A12^{KD}$ alone, no additional changes in proliferation were observed (Figure RL9B), suggesting that SLC25A12 acts independently of SLC25A13. Moreover, SLC25A13 expression did not increase in response to SLC25A12 knockdown (Figure RL9C), suggesting a lack of compensatory regulation. These findings imply that, in this particular cell type, proliferation is more dependent on SLC25A13 than on SLC25A12.

It is important to note that this dependency is not universal across all cell types. For example, in hepatocellular carcinoma, proliferation has been shown to rely on SLC25A12 (Infantino *et al*, 2019). Additionally, epigenetic mechanisms likely play a key role in regulating the expression of these mitochondrial transporters. These mechanisms enable cells to upregulate transporters in response to changing biosynthetic demands—such as protein, nucleotide and fatty acid synthesis—thus supporting survival and growth. Further investigation into the epigenetic regulation and reprogramming of SLC25A12 and SLC25A13 is warranted to elucidate their distinct roles in endothelial cells.

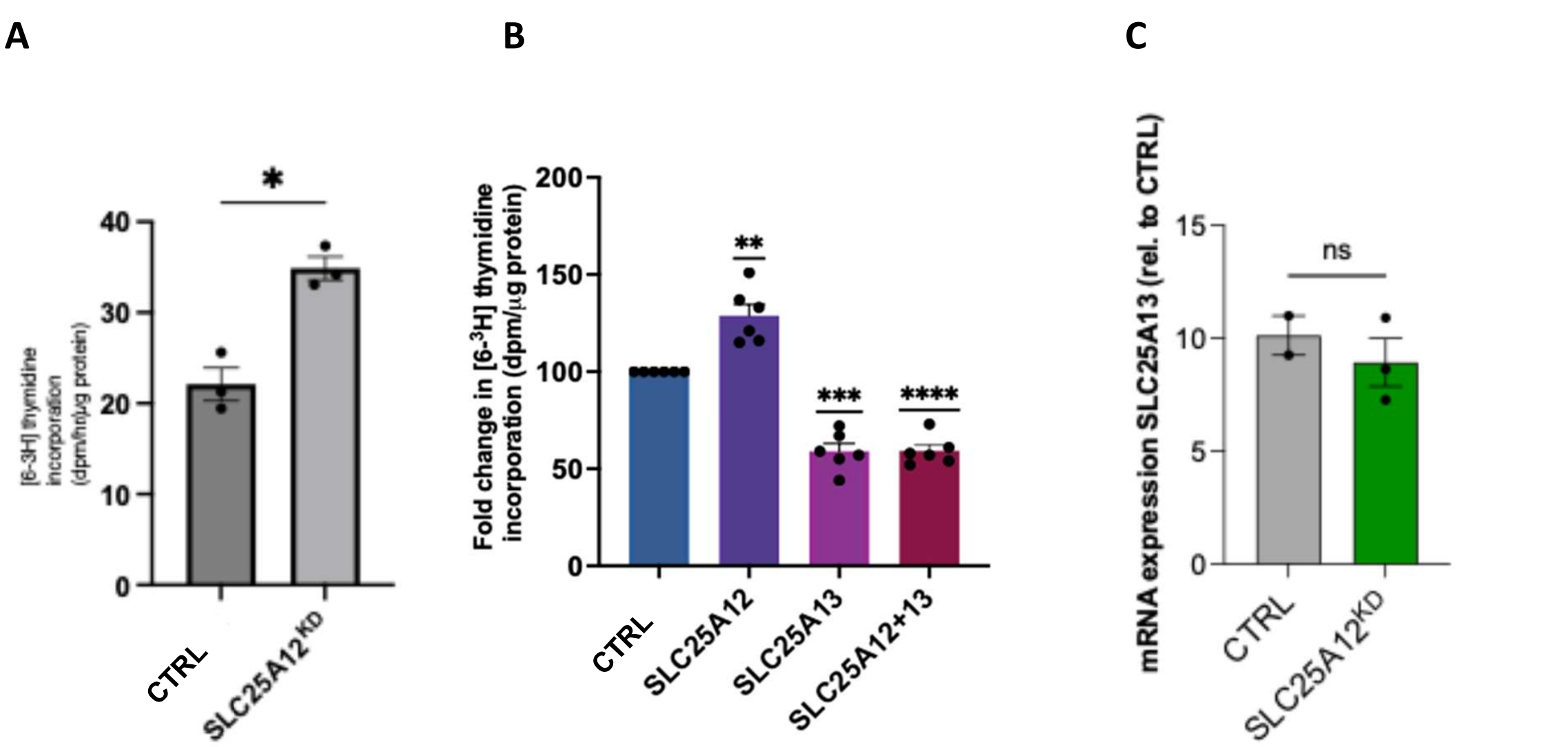

Figure RL9. A.[6-3H]-Thymidine and EDU incorporation into DNA in CTRL and $SLC25A12^{KD}$ ECs (n=3). B. [6-3H]-Thymidine and EDU incorporation into DNA in CTRL, $SLC25A12^{KD}$, $SLC25A13^{KD}$ and $SLC25A12^{KD}$ / $SLC25A13^{KD}$ ECs (n=6). C**.** mRNA expression (RT-qPCR) of SLC25A13 in CTRL and $SLC25A12^{KD}$ ECs (n=3). Data are means ± SEM; not significant, *p<0.05 by two-tailed paired t-test (A, C). **p<0.01 ***p<0.001, **** p<0.0001 by one-way ANOVA (B)**.**

(Figure RL9.C is included as Figures EV2D in the revised manuscript)

3) The authors analyzed the role of SLC25A13 in a model of laser-induced neovascularization using siRNA tested in vitro in bEnd3 cells. A) Did the authors evaluate the protein levels of SLC25A13 and SLC25A12 in vivo endothelial cells murine (mECs)?

Response: In vivo, the levels of SLC25A13 were only checked at the RNA level (after EC sorting) because the amount of isolate ECs was not sufficient to also check the protein level. However *in vitro*, we checked both RNA and protein until 48h after silencing for SLC25A13 in other murine cell types. As shown in the following figures (Figs. RL10, RL11), silencing of SLC25A13 indeed causes a significant reduction in the RNA and protein levels.

In vivo* (mRNA) levels*:

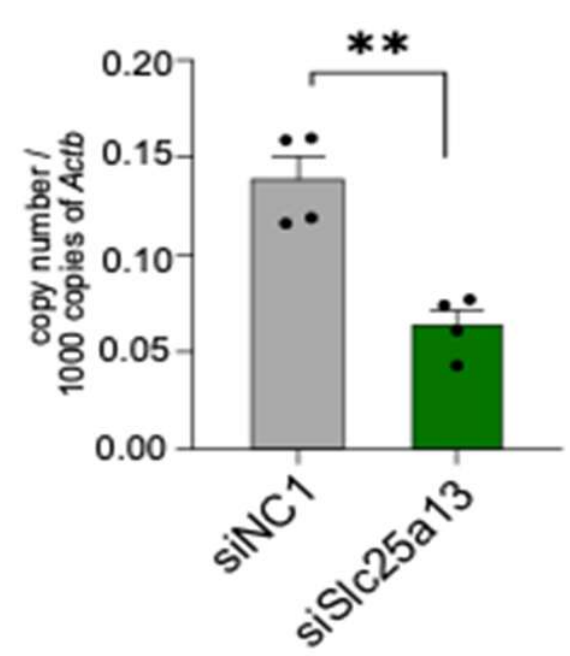

Figure RL10. (Fig. EV2E in the revised manuscript) Transcript levels of Slc25a13 in ECs isolated from siNC1 or siSLC25A13 treated neovascularized choroids (n=4). Data are means ± SEM; **p<0.01, by two-tailed paired t-test .

In vitro in murine MS1 and H5V cells:

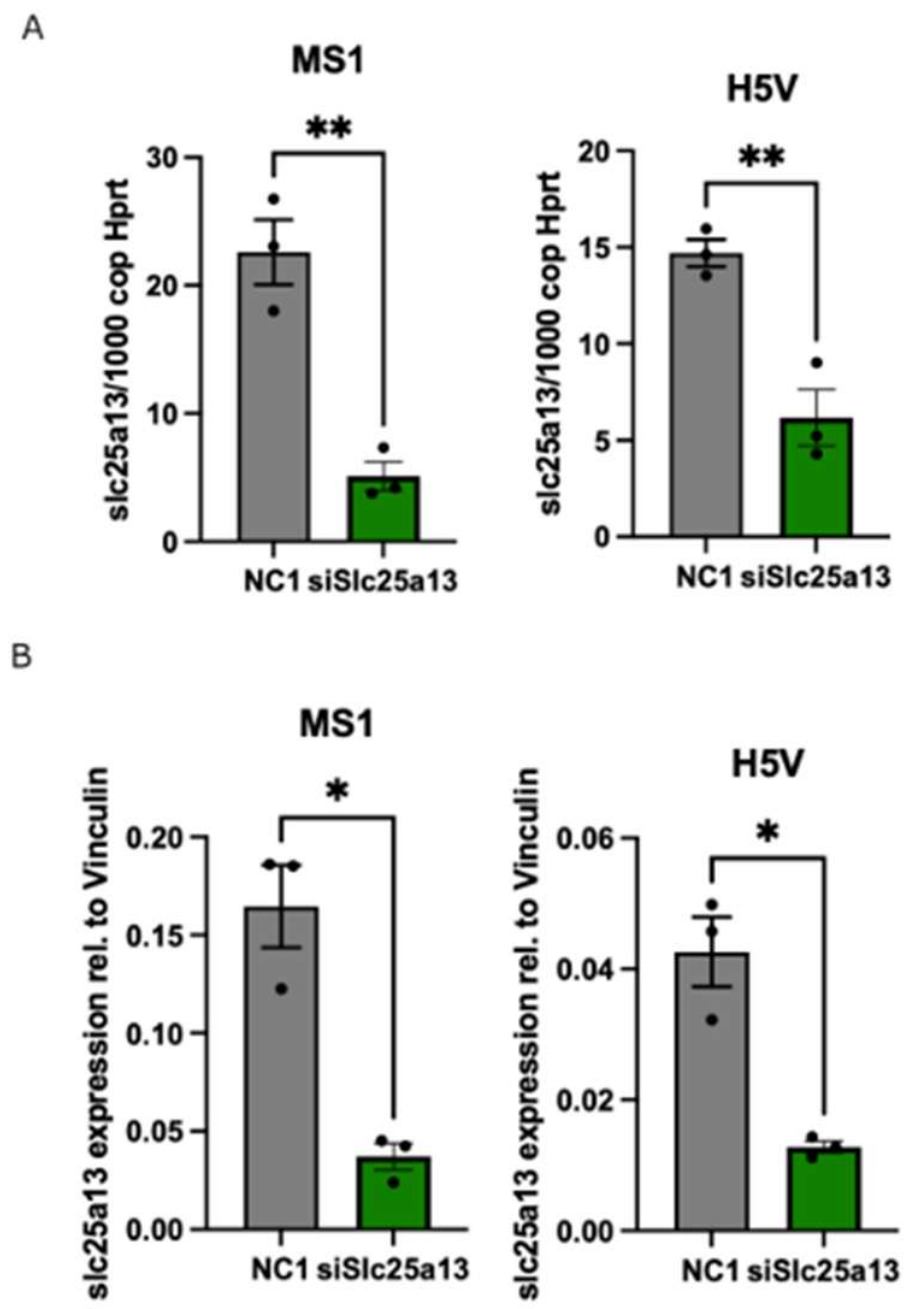

Figure RL11. A. mRNA expression (RT-qPCR) of slc25a13 in siNC1 (negative control) or Slc25a13-specific siRNA transfected MS1 and H5V murine cells after 48h (n=3). B.Protein expression (western blot) of slc25a13 in siNC1 (negative control) or Slc25a13-specific siRNA transfected MS1 and H5V murine cells after 48h (n=3). Values are data from densitometric analysis of western blots for slc25a13, normalized to Vinculin (n=3). Data are means ± SEM; not significant, *p<0.05, **p<0.01, by two-tailed paired t-test (A,B).

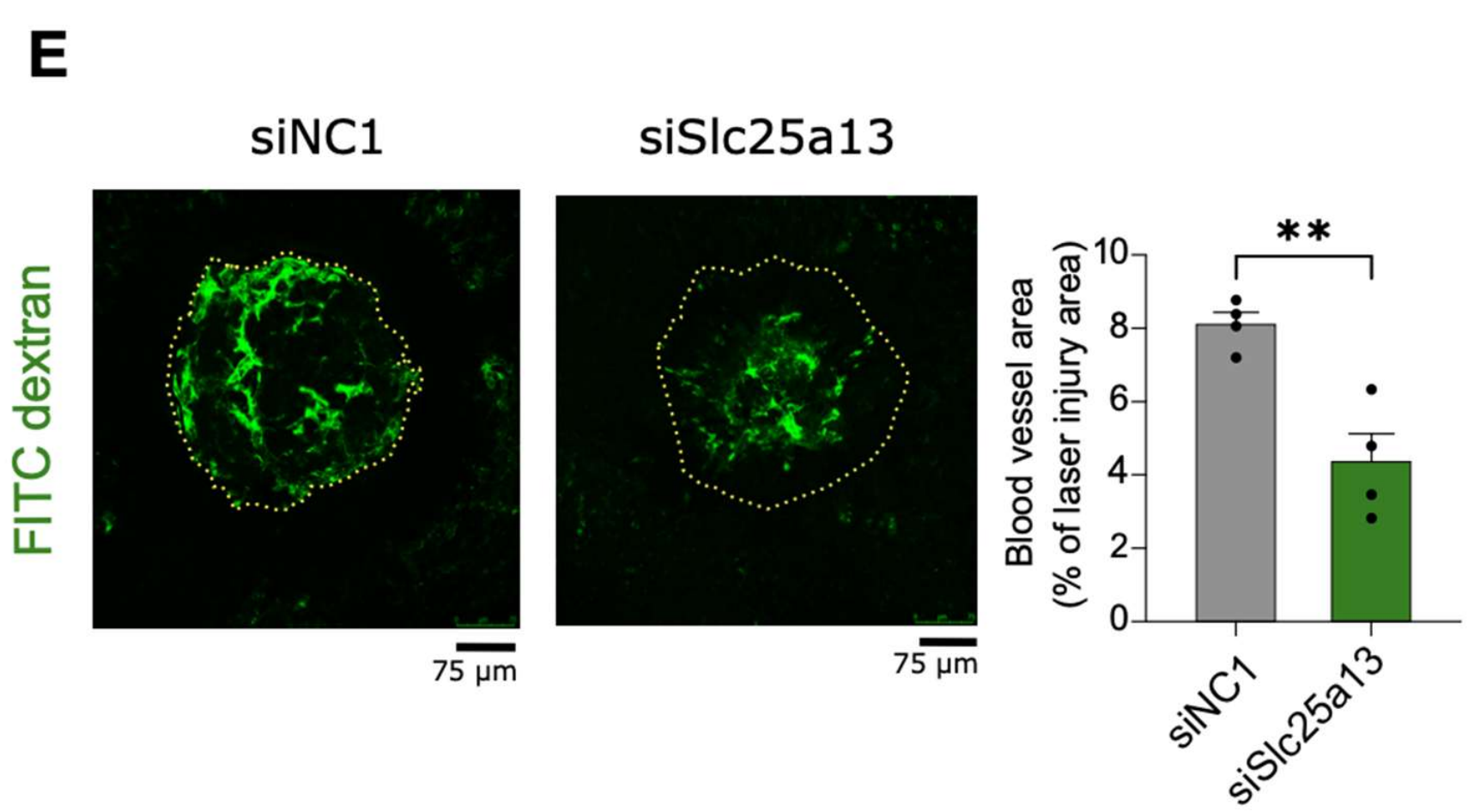

Figure 2E. In vivo choroidal neovascularization upon intravitreal injection of a siNC1 (negative control)) or Slc25a13-specific siRNA (siSlc25a13). Left: representative images of FITC-Dextran stained (green, visualizing the neovasculature) choroidal flatmounts. Right: Quantification of

choroidal neovascularization (blood vessel area; green) normalized to laser injury area (demarcated by dotted line in the images) (n=4) (each datapoint represents the mean of the three mice of an independent replicate experiment). Scale bars: 75 µm. Data are means ± SEM; *p<0.05, **p<0.01, ***p<0.001 by two-tailed paired t-test (A, C, D); ****p<0.0001 by simple linear regression analysis (B); **p<0.005 by two-tailed unpaired t test (E). CTRL – HUVECs transfected with control (nonsense scrambled) shRNA.

B) What happens in a model of physiological angiogenesis, such as retinal angiogenesis, when SLC25A13 is deleted?

These in vivo experiments should be performed with SLCA13 conditional allele Tg animals since with siRNA a non EC autonomous function of SLCA13 can not be excluded.

Response: As previously agreed with the editor, generating and phenotyping transgenic endothelial cell-specific conditional knockout mouse is costly (30-50K€ for a workable colony of syngeneic strains) and requires several years and was agreed on not necessary to include for this revision. In addition, the retinal angiogenesis model is not (always) predictive for pathological angiogenesis in disease (Gariano & Gardner, 2005; Liu *et al*, 2017).

From the numerous papers on EC metabolism, it is very clear that the in vitro vessel sprouting data (using EC spheroids) is highly predictive for the angiogenesis phenotype in vivo – in other words, whenever we detected a sprouting defect in vitro, we noticed an angiogenic defect in vivo (Chrzanowska-Wodnicka *et al*, 2008; De Bock *et al*, 2013; Vandekeere *et al*, 2018; Wimmer *et al*, 2012).

4) Considering that the authors observe an increase in the glycolysis (Fig. 4I), endothelial cells (ECs) need to regenerate oxidized NAD+ when mitochondrial function is impaired. The authors also showed a significant increase in the MDH1 enzyme, which converts oxaloacetate (OAA) into malate while oxidizing NADH. Could be interesting analyze the fluxomic of glucose in SLC25A13 vs. CTRL. How did you explain the increase of SLC25A11, the mitochondrial transporter of malate? And, a reduction of SLC25A1 a citrate carrier connected with citrate-malate shuttle?

Response: We first would like to emphasize that glycolytic flux remains unchanged as shown in Fig. 4N of the main manuscript. If the reviewer is referring to glucose oxidation, we had previously shown an increase in glucose oxidation using the glucose oxidation assay (Fig. 4J). In accordance with the reviewer's suggestions, we performed a ^{13}C isotope glucose tracing experiment to verify the roles of glycolysis and glucose oxidation during SLC25A13 perturbation. Here are some key observations of the ^{13}C glucose isotope tracing experiment:

(i) No significant change in NADH / NAD ratio or ROS levels: Although a decreasing trend in NAD/NADH ratio is observed, the changes are not statistically significant. We confirmed this result using an independent NADH/NAD kit (see response to Question 7 or Reviewer 2). Similarly, CM-H_2DCFDA fluorescence – a proxy for ROS levels - remains unchanged following SLC25A13 knockdown. This finding suggests that overall redox balance and hence, global reactive oxygen species (ROS) production, are not substantially affected. Consistent with this, no significant alterations were captured in ^{13}C-glucose carbon labelled TCA cycle intermediates (pyruvate, citrate and malate) (Figure RL6B) suggestive of the role of glucose oxidation in maintaining redox balance. At the transcription level, MDH1 and SLC25A1 show slight but statistically significant changes in transcript expression (MDH1 log2FC=0.143 and SLC25A1 log2FC=-0.22) but SLC25A11 is not significantly altered (Table EV2). Taken together, the observed upregulation of MDH1 or reduction in SLC25A1 might be a compensatory transcriptional response towards $SLC25A13^{KD}$ rather than an actual metabolic shift.

(ii) Significant change in aspartate and nucleotides: As observed from the ^{13}C glucose and ^{13}C glutamine isotope tracing experiments (Fig 4A – ump, utp and ctp from ^{13}C glutamine and Fig. EV3D – gmp from ^{13}C glucose), glucose- and glutamine-derived aspartate and nucleotide levels are reduced upon SLC25A13 perturbation confirming its role in biomass synthesis.

(iii) No change in cholesterol levels: There is no significant change in ^{13}C glucose labelled cholesterol levels upon $SLC25A13^{KD}$ (Figure RL6C). Moreover, the percent level of cholesterol labelled by glucose is minimal suggesting cholesterol is primarily formed from other sources. Glutamine-labelled mevalonate levels are drastically reduced upon $SLC25A13^{KD}$ (Fig. 4C). $SLC25A13^{KD}$ drastically reduces cholesterol abundance in ECs (Fig. 4D). $SLC25A13^{KD}$ cell proliferation is also rescued partially by mevalonate (a precursor of cholesterol synthesis), suggesting a glutamine-linked role of SLC25A13 in biomass

synthesis. This is also consistent with the transcriptional down- regulation of squalene and cholesterol synthesis gene sets.

To summarize, we hypothesize that SLC25A13 plays an essential role in overall biomass synthesis, although selectively acting through glutamine-degradation-linked mevalonate synthesis.

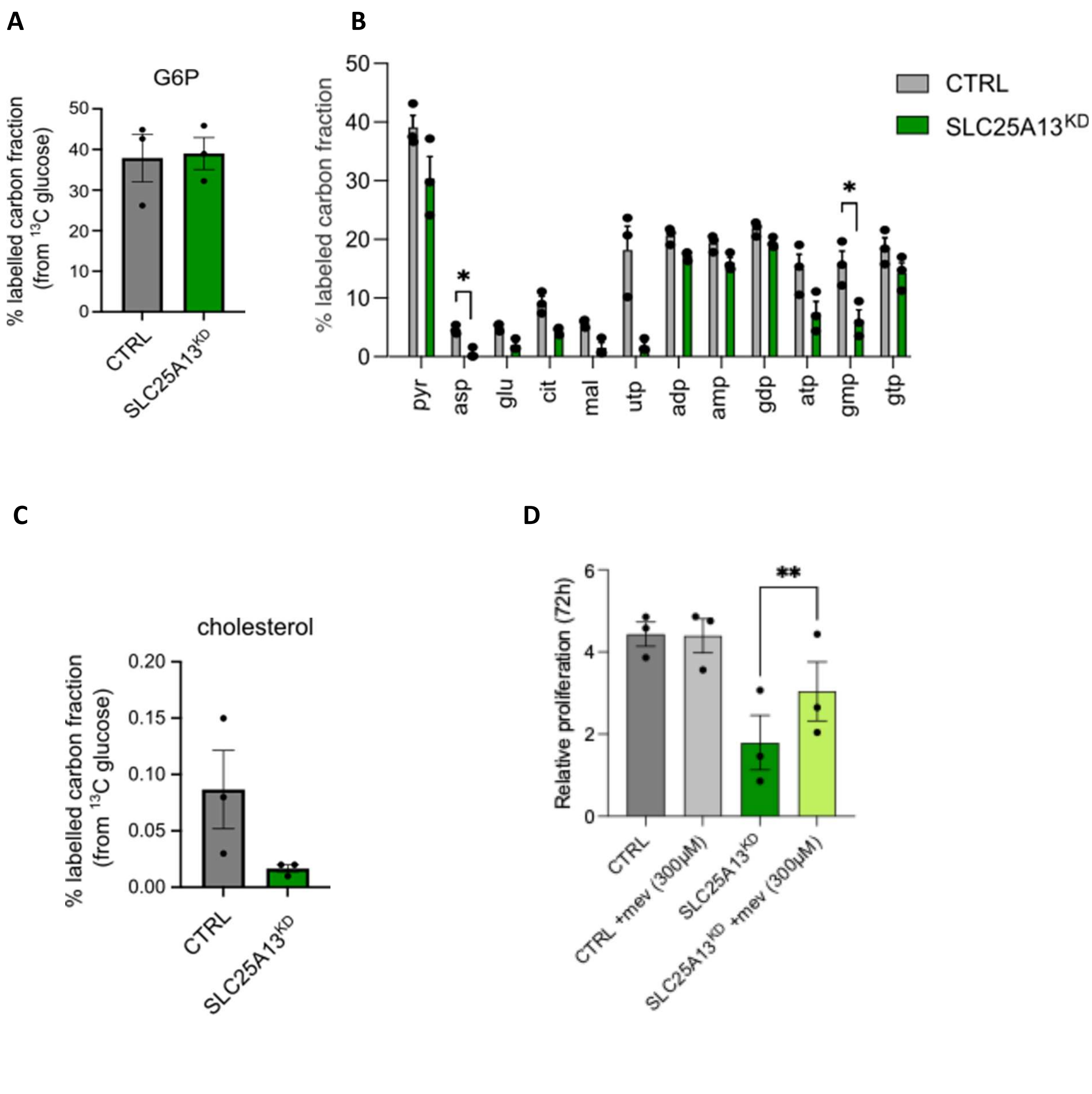

Figure RL6. A. Carbon fraction of glucose-6-phosphate (G6P) labelled from U-13C glucose (n=3). B. Bar plots representing the total fraction of [U-13C]-glucose carbon in pyruvate (pyr), amino acids (asp, glu), TCA metabolites (cit, mal), nucleotides (ump, adp, amp, gdp, atp, gmp, gtp) and cholesterol precursor (mev) (n=3). C. Carbon fraction of cholesterol labelled from U-13C glucose (n=3). D. Rescue with excess Mevalonate: Barplots demonstrating the comparison between CTRL and SLC25A13KD ECs grown in EGM2 + 300µM Mevalonate after 72h (n=3). Data are means ± SEM; **p<0.01 by one-way ANOVA. adp: adenosine diphosphate, atp: adenosine triphosphate, amp: adenosine monophosphate, asp: aspartate, cit: citrate, gdp: guanosine diphosphate, glu: glutamate, gmp: guanosine monophosphate, mal: malate, pyr: pyruvate, utp: uridine triphosphate. Data are means ± SEM; not significant, by a two-tailed paired t-test. Rescue with excess Mevalonate: Barplots demonstrating the comparison between CTRL and SLC25A13KD ECs grown in EGM2 + 300µM Mevalonate after 72h (n=3). Data are means ± SEM; **p<0.01 by one-way ANOVA. **(Figs. RL6A - C is implemented as Figs. EV3C - 3E respectively. Fig. RL6D is included as Fig. 4E in the revised manuscript)**

5) In the experiments of rescue using an excess of cholesterol, the treatment induced an increase of proliferation of CTRL ECs, because that it is not a functional rescue of the phenotype (Fig. 4D). The link with cholesterol synthesis, although novel, is not supported by the data. Cholesterol supplementation is able to increase proliferation also in wt cells at the same rate as silenced cells, blunting a specific rescue effect on siSLC25A13 compared to controls. Also, cholesterol is not the only mevalonate final products: mevalonate could be used for rescue as well. Similar comments and conclusion for the effect of dNTP+Glu treatments (Fig. 4E).

Response: Although cholesterol is a downstream product of mevalonate, its supplementation did not specifically rescue the SLC25A13^{KD} ECs (Fig. EV4B). As rightfully suggested by the reviewer, cholesterol is not an exclusive product of mevalonate and can also form non-sterol metabolites. Thus we attempted to rescue SLC25A13^{KD} EC proliferation directly using mevalonate itself. We thank the reviewer for this insightful comment. As suggested, we performed additional experiments using mevalonate supplementation (300 µM) to evaluate whether the rescue effect was specific to mevalonate (Figure RL12). Notably, mevalonate treatment resulted in a selective and statistically significant partial rescue of proliferation in

SLC25A13^{KD} cells. This indeed reflects a specific dependence of the knockdown phenotype on mevalonate-derived metabolites. This also supports the decrease in cholesterol abundance observed in SLC25A13^{KD} ECs as mevalonate serves as a precursor for cholesterol synthesis (Fig. 4D). As shown previously using ^{13}C glutamine-labelling experiments, glutamine-derived succinate mediated by SLC25A13 is responsible for mevalonate biosynthesis, a novel pathway previously unexplored in ECs (Fig. 3G, Fig. 4C). We hypothesize that restoring mevalonate might promote the recovery of both sterol and non-sterol metabolites. Therefore, the rescue achieved by administering mevalonate to the cells more directly demonstrates that the observed effects result from inhibition of the mevalonate pathway rather than cholesterol depletion alone. We have included the new mevalonate rescue data in the revised manuscript (Fig. 4E) and updated the discussion accordingly.

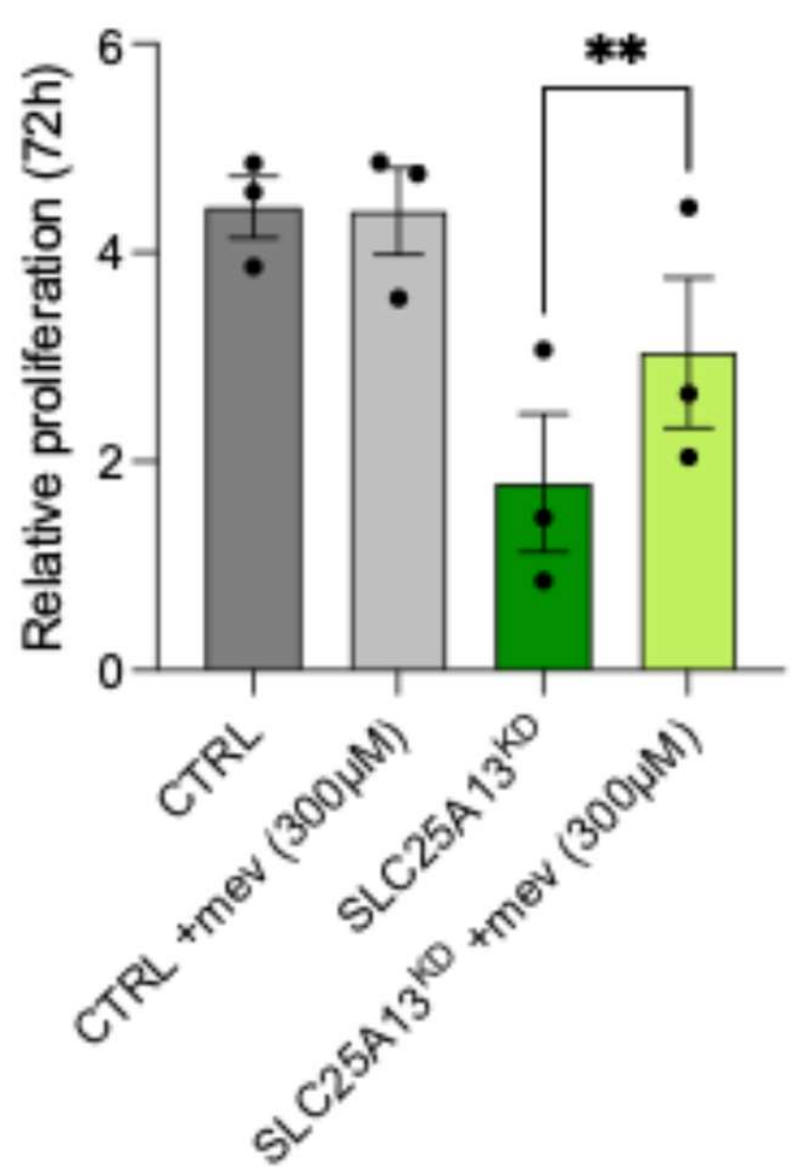

Figure RL12. Rescue with excess Mevalonate: Barplots demonstrating the comparison between CTRL and SLC25A13KD ECs grown in EGM2 + 300µM Mevalonate after 72h (n=3). Data are means ± SEM; **p<0.01 by one-way ANOVA.

(Figure RL12 is included as Fig. 4E in the revised manuscript)

6) How the authors explain metabolically the increase in migration of ECs under SLC25A13 KD?

Response: This appears to be a misunderstanding. In our experiments, SLC25A13 knockdown actually leads to a decrease in wound closure capacity and reduced blood vessel branching, indicating impaired, not enhanced, EC migration (Figure 2 in the main manuscript). Also, it is important to note that SLC25A13 knockdown leads to strong defects in mevalonate and cholesterol (Figs. 4C, 4D), which might impact membrane changes during EC migration. Similarly, reduced amino acids (Figs. 4A, 4B) impact protein synthesis (Fig. 4H) that might be required for sustaining signaling pathways and protein turnover during migration.

7) The graph of NADH/NAD present a lot of dispersion, since it is a key point of the paper it should be improved (Fig. 4J).

Response: We thank the reviewer for this insightful comment. We acknowledge that the NADH/NAD^+ ratio data presented (see below) displays a degree of variability across biological replicates. This variability likely reflects the intrinsic metabolic heterogeneity of ECs from different human donors, especially under conditions of metabolic stress such as SLC25A13 knockdown. To confirm whether NADH/NAD^+ variability is indeed biological in nature and not an experimental artefact, we performed an additional experiment using an independent NADH / NAD assay kit. The overall trend still indicated no significant change in the NADH/NAD^+ ratio (Figure RL13), which is reproducible across independent experiments. Similarly, CM-H_2DCFDA fluorescence – a proxy for ROS levels, remains unchanged following SLC25A13 perturbation (Fig. 4K). This finding suggests that maintaining overall redox balance is not the primary function of SLC25A13. We believe this highlights a specific metabolic vulnerability rather than a generalized stress response.

NADH/NAD ratio as measured by metabolomics:

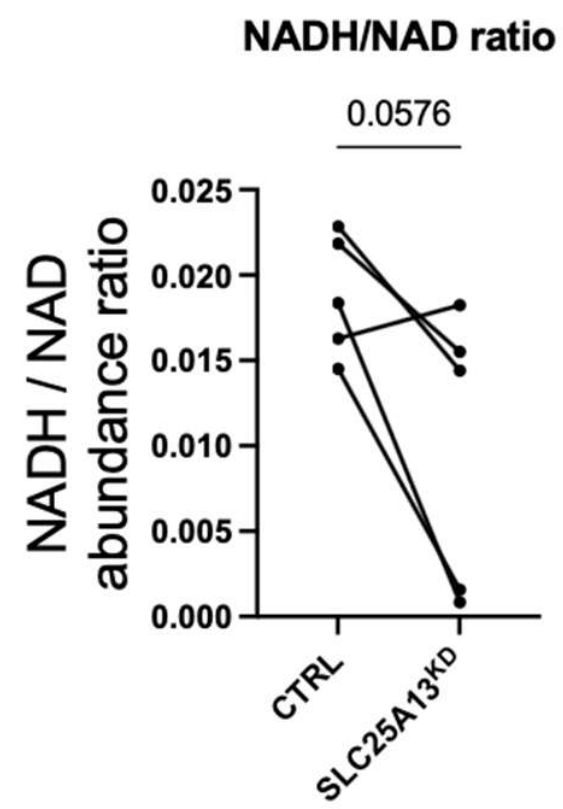

Our observation of unchanged NADH/NAD ratio were confirmed by a different assay, more specifically by the use of a NADH/NAD kit:

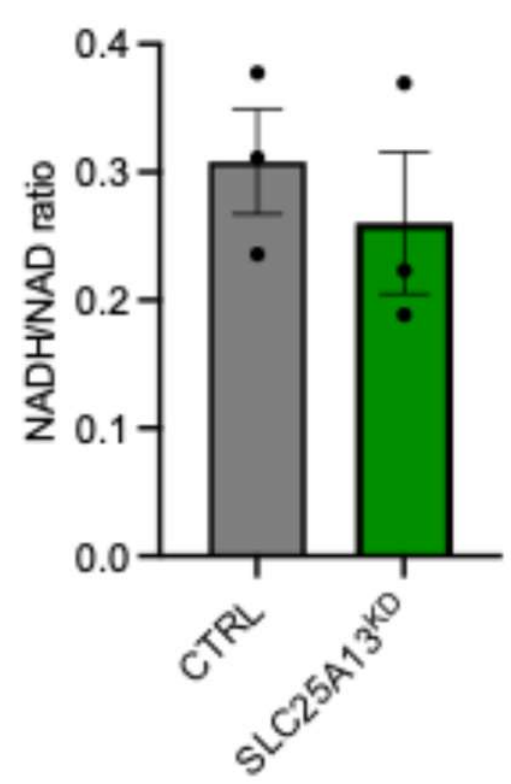

Figure RL13. Change in NADH/NAD+ ratio between CTRL and SLC25A13KD ECs. Data are means ± SEM; no significance by two-tailed paired t-test.

(Figure RL13 is included as Figure EV4E in the revised manuscript)

8) The authors stated that RNA seq analyses have been performed on HUVEC silenced for SLC25A13 for 6 days. The level of SLC25A13 downregulation in these samples is not provided.

Response: SLC25A13 expression levels were checked on the 6th day by RT-qPCR in HUVECs silenced with shRNA (Fig. RL14A). Besides, we also observed the same silencing from our bulk RNA seq data (Fig. RL14B). This expression decrease leads to a prominent decrease at the protein level (Western Blot) (Fig. RL15).

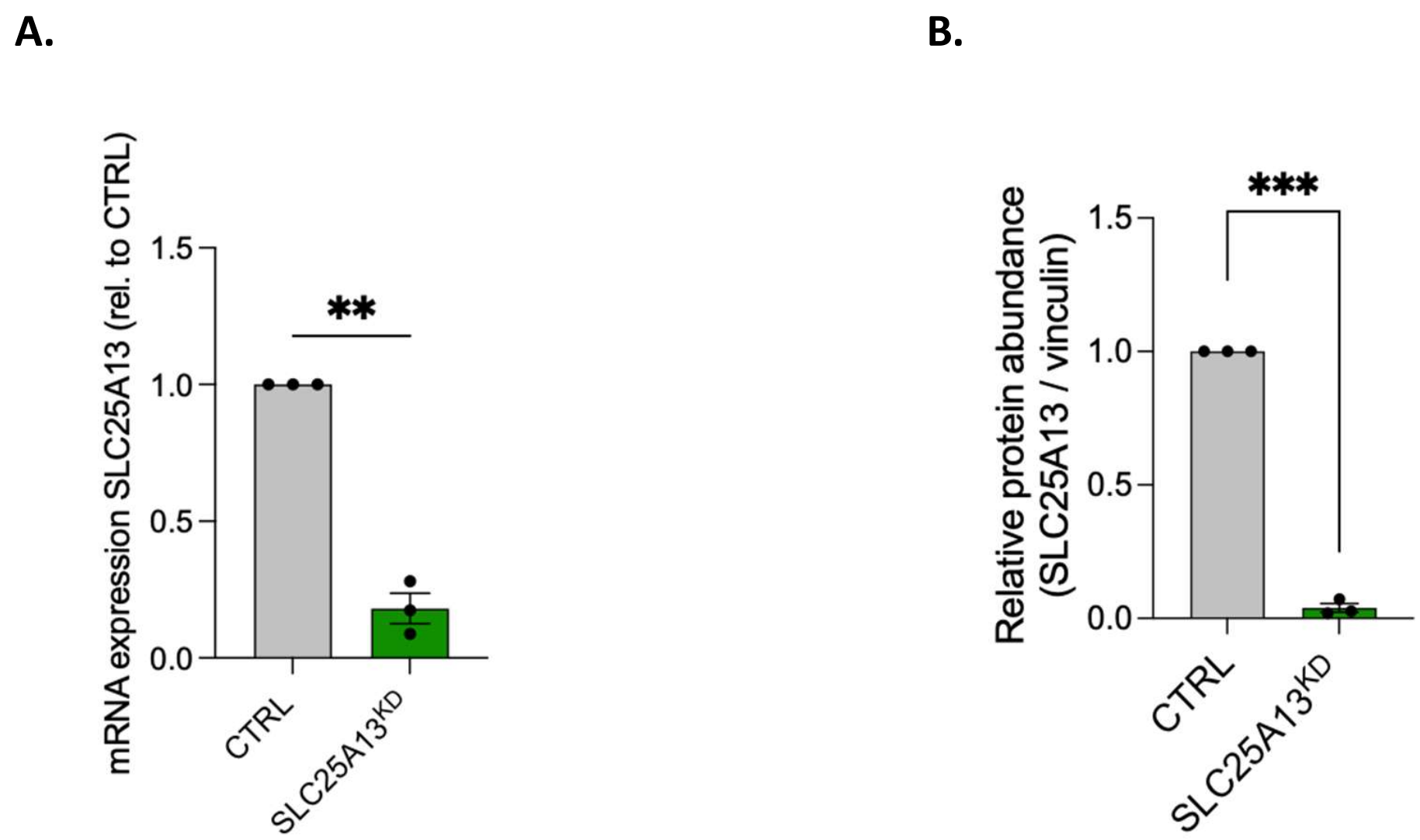

Figure RL14. A. mRNA expression (RT-qPCR) of SLC25A13 in CTRL and SLC25A13KD ECs (expression relative to control) (n=4-5). B. Transcript levels of SLC25A13 in HUVECs (expression normalized to HPRT). Bar graphs show bulk data. Data are means ± SEM; **p<0,01 by two-tailed paired t-test (A,B).

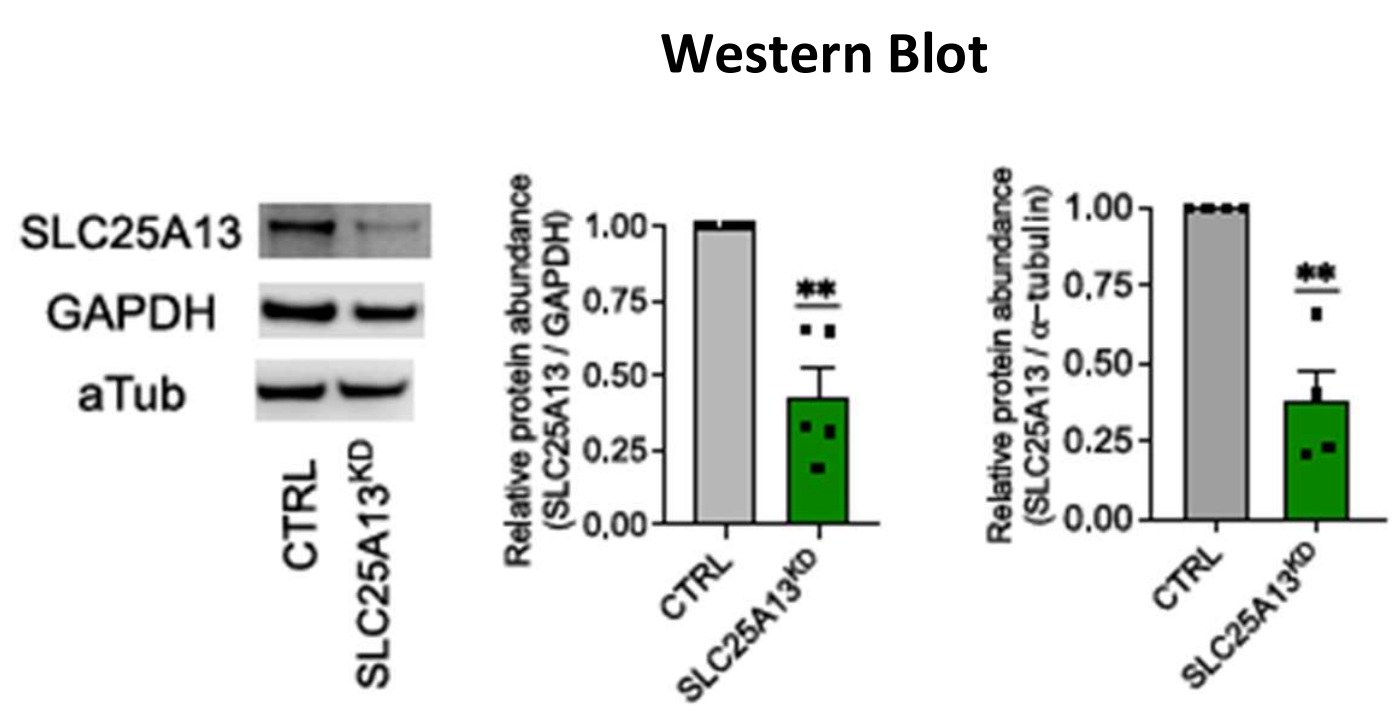

Figure RL15 (Fig. EV2B). Representative immunoblot and densitometric quantification of SLC25A13 protein levels in CTRL and SLC25A13KD ECs (expressed relative to control) (n=4 and n=5, respectively). GAPDH and α-Tubulin was used as a loading control. Data are means ± SEM; **p<0.01 by one sample t-test and Wilcoxon test.

Minor

Introduction: Paragraphs 1 and 2 should be combined into one paragraph. And, clarify in the introduction that SLC25A13 is one of the transporters involved in the malate-aspartate

shuttle (MAS). The introduction currently lacks an explanation of this metabolic shuttle and the role of AGC and OGC.

Response: As suggested by the reviewer, Paragraphs 1 & 2 were combined into one paragraph (yellow highlighted on Page 3). We also added a line introducing SLC25A13 as a component of the aspartate-malate shuttle (yellow highlighted on Page 4).

Recently, it has been described that aspartate-derived glutamine is crucial for supporting EC proliferation (PMID: 35580611). In fact, it has been shown through 13C fluxomic analysis that glutamine is essential for synthesizing aspartate. The authors should cite and comment previous work carefully when mentioning 'in previous studies in non-ECs...'

Response: As requested by the reviewer, the appropriate references were cited.

Please, add the flux of electrons in Figure 1F considering that the main role MAS is to transport electrons across the mitochondrial membrane, not merely the passage of metabolites.

Response: As per the reviewer's suggestion, Figure 1F has been revised to show the electrons associated with the malate aspartate shuttle across the mitochondrial membrane.

Please, cancel "Consistently, the reaction associated with mitochondrial to cytosolic aspartate transport ..." In the subsequent sentence, use the abbreviation RKo for the "in-silico reaction knockout" mentioned above. The authors used ASPLGUm as the abbreviation for mitochondrial aspartate-glutamate transporter. Please use AGC instead.

Response: We modified the sentence to address the reviewer's concerns (yellow highlighted on Page 7). We retained the abbreviation ASPGLUm, as this is the reaction abbreviation corresponding to the aspartate-glutamate antiport reaction that is enlisted within the EC-GEM metabolic model. This reaction abbreviation will help the reader connect the model results to the observations reported in the paper.

Fig. 2A, correct the (%.) for (%) in the legend of EdU positive cells graph. Please, add the scale bar Fig. 2C.

Response: The correction for Fig. 2A and Fig. 2C are performed and the revised figures are provided.

Please, cancel 'which is consistent with the negative enrichment of angiogenesis...' in the last paragraph of the section Results -SCL25A13 is essential for EC proliferation and angiogenesis-, because the analysis of RNAseq is describe in the follow section. Please change 'glutamate-aspartate shuttling' to 'malate-aspartate shuttling.' When referring to the transporter, use 'carrier' or 'transporter'.

Response: The corresponding sentences have been revised (yellow highlighted on Page 8).

Some graphs of 13C-Q fluxomic analysis, including alpha-ketoglutarate (M+5) (Fig. 3E) and aspartate (M+4)(Fig. 3F) levels are significant when CTRL vs. SLC25A12KD are compared, please can you check this information.

Response: Yes, we confirm that comparison between CTRL and SLC25A13^{KD} is significant in Figs. 3E and 3F.

In the discussion, in the second paragraph please correct this sentence: 'It has been documented that different' by 'It has been documented two different...'

Response: The revision was performed in the main manuscript. It is yellow highlighted on Page 13.

In the discussion, in the fourth paragraph the authors suggest a shift of glutamine oxidative phenotype, but the results did not indicate and increase of oxidative glutaminolysis (fluxomics and decrease of GLS). So, remove the sentence.

Response: As per the reviewer's suggestion, we have made the corresponding change.

M&M section: it is not clear what this statement means: "All genes that were expressed in at least 6 of the total 12 samples .... were retained for further analysis". Es: Could be that 5 samples have been used as controls on 1 sample in the KD conditions? Please explain precisely how and which samples of ctrl and KD have been used for transcriptomics analyses.

Response: The analysis was done using the "filterByExpr" command in edgeR with a min.prop of 6 and min.count of 5. This ensures that there are at least 3 samples in each condition (so, in total 6 out of 12 samples) in which a gene is expressed.

Referee #3 (Comments on Novelty/Model System for Author):

Minor comments are in the remarks to the author.

Referee #3 (Remarks for Author):

The manuscript is logical and well written. Beyond further highlighting the importance of cell metabolism for energy and biomass production, the manuscript is a nice application of "modeling" to guide efficient experiments.

This reviewer would offer a few suggestions for consideration by the authors.

Response: We thank the reviewer for acknowledging our interdisciplinary work involving the development of biologically relevant models and their validation through experiments.

1) It seems the modeling led to the identification of two transporters. The silencing of one resulted in the finding and the silencing of the other had no effect. Can the inverse of this be established, specifically can the modeling identify a point that is not predicted to have an effect and can the negative predictive value of the modeling be established. This would not require the entire battery of experiments, merely enough to extend the validation utility of the modelling.

Response: We thank the reviewer for this insightful suggestion. As provided during the initial submission, Table EV1 contains the information of 30 curated, experimentally assessed, essential and non-essential genes (enzymatic reactions) for biomass synthesis in ECs; out of which there are 6 known non-essential genes / reactions. The EC-GEM under FAO-dependent constraints predicts all the six non-essential reactions as non-essential, demonstrating perfect accuracy in identifying true negatives.

As per the reviewer's suggestion, we evaluated the Negative Predicted Value (NPV) of the EC-GEM essentiality. Under FAO-dependent conditions, the model's positive predictive value (PPV) was 1.0, and the NPV was 0.5 (revised Table EV1). The lower NPV reflects the fact that a few experimentally verified essential genes are predicted as non-essential by the model (i.e., false negatives).

Taken together, these results indicate that when the EC-GEM predicts a reaction to be essential, it is highly likely to be truly essential (high PPV). The model also demonstrates

excellent accuracy in identifying non-essential genes (true negatives). However, it might fail to capture all essential genes, highlighting an area for potential refinement.

2) Is data available on the extent of modulation of the target that was required for the experimental read-outs to be appreciated? Perhaps this was in the modeling and could be emphasized. It could be fair to say that metabolism is a "tightly-regulated" process. Understanding the extent of variation in target expression/activity is needed would be helpful in the generalizability/applicability of the (very interesting) findings.

Response: We appreciate the reviewer's thoughtful comment. The extent of modulation required to observe an experimental effect is indeed an important consideration given that metabolism is a tightly regulated process. In our study, the primary goal was to uncover novel pathways (metabolite utilization routes) and genes involved in metabolite utilization in ECs, many of which lacked prior functional characterization. For this purpose, we chose a genome-scale metabolic modelling and flux balance analysis approach that offers estimation of reaction fluxes across the entire metabolic network under the assumption of pseudo steady state. These reaction fluxes help to discover novel utilization routes of metabolites during biomass synthesis. The identification of SLC25A13, its link to glutamine utilization and contribution to biomass synthesis (e.g. cholesterol and nucleotide synthesis) through the EC-GEM analysis illustrates the benefit of using genome-scale models in hypothesis generation and validation.

However, we acknowledge that this approach does not capture the non-linear, dose-dependent effects of partial gene modulation. The linear nature of flux balance analysis limits its ability to predict how varying degrees of target knockdown or inhibition might influence biomass synthesis or other phenotypic outputs. This is an inherent limitation of the method. We explicitly acknowledge this limitation in the Discussion, under the section "Limitations". Incorporating dynamics of target modulation would require complementary modelling approaches (e.g. kinetic modelling), which we view as an important future direction.

3) The purely in-vitro parts of the study were in one EC type. As from the above, there would be no need to repeat the entire study in another cell line (or in male vs. female) but some data confirming that findings are not restricted to HUVEC would be ideal.

Response: To not only limit our findings to HUVECs, we also used siRNAs to silence SLC25A13 in ECs *in vivo* in a model of choroidal neovascularization (CNV), where we do see a decrease in blood vessel area formation, confirming our findings not only in HUVECs but also *in vivo* on angiogenesis. In addition, as per the reviewer's suggestion, we tested the silencing of SLC25A13 using reported siRNAs in multiple murine EC cell lines *in vitro*. We could see a significant silencing of SLC25A13 in multiple murine cell lines as well (Fig. RL11). The same siRNAs were then used to silence SLC25A13 *in vivo* in ECs and functionally validated (Fig. 2E). Importantly, SLC25A13 knockdown in MS1 (murine pancreatic islet endothelial cell line) and HMVEC-L (Human Lung Microvascular Endothelial Cells) cells resulted in a significant reduction in cell proliferation and migration, mirroring the effects observed in HUVECs. Altogether, these results demonstrate that our findings are not restricted to HUVECs and can be extended to other endothelial cell models (Fig. RL16).

This text referred to a figure for reviewers that was removed because it displayed unpublished data. The text also referred to this data and was consequently removed.

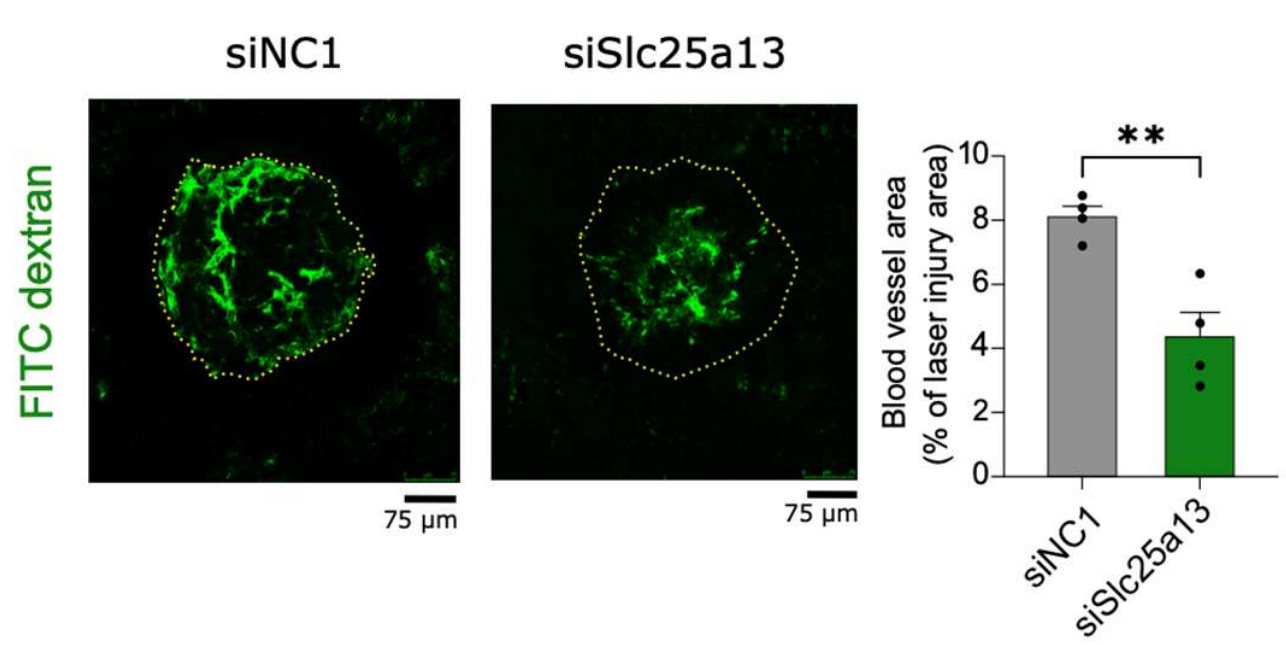

Figure 2E. In vivo choroidal neovascularization upon intravitreal injection of a siNC1 (negative control)) or Slc25a13-specific siRNA (siSlc25a13). Left: representative images of FITC-Dextran stained (green, visualizing the neovasculature) choroidal flatmounts. Right: Quantification of choroidal neovascularization (blood vessel area; green) normalized to laser injury area (demarcated by dotted line in the images) (n=4) (each datapoint represents the mean of the three mice of an independent replicate experiment). Scale bars: 75 µm. Data are means ± SEM; *p<0.05, **p<0.01, ***p<0.001 by two-tailed paired t-test (A, C, D); ****p<0.0001 by

simple linear regression analysis (B); **p<0.005 by two-tailed unpaired t test (E). CTRL – HUVECs transfected with control (nonsense scrambled) shRNA.

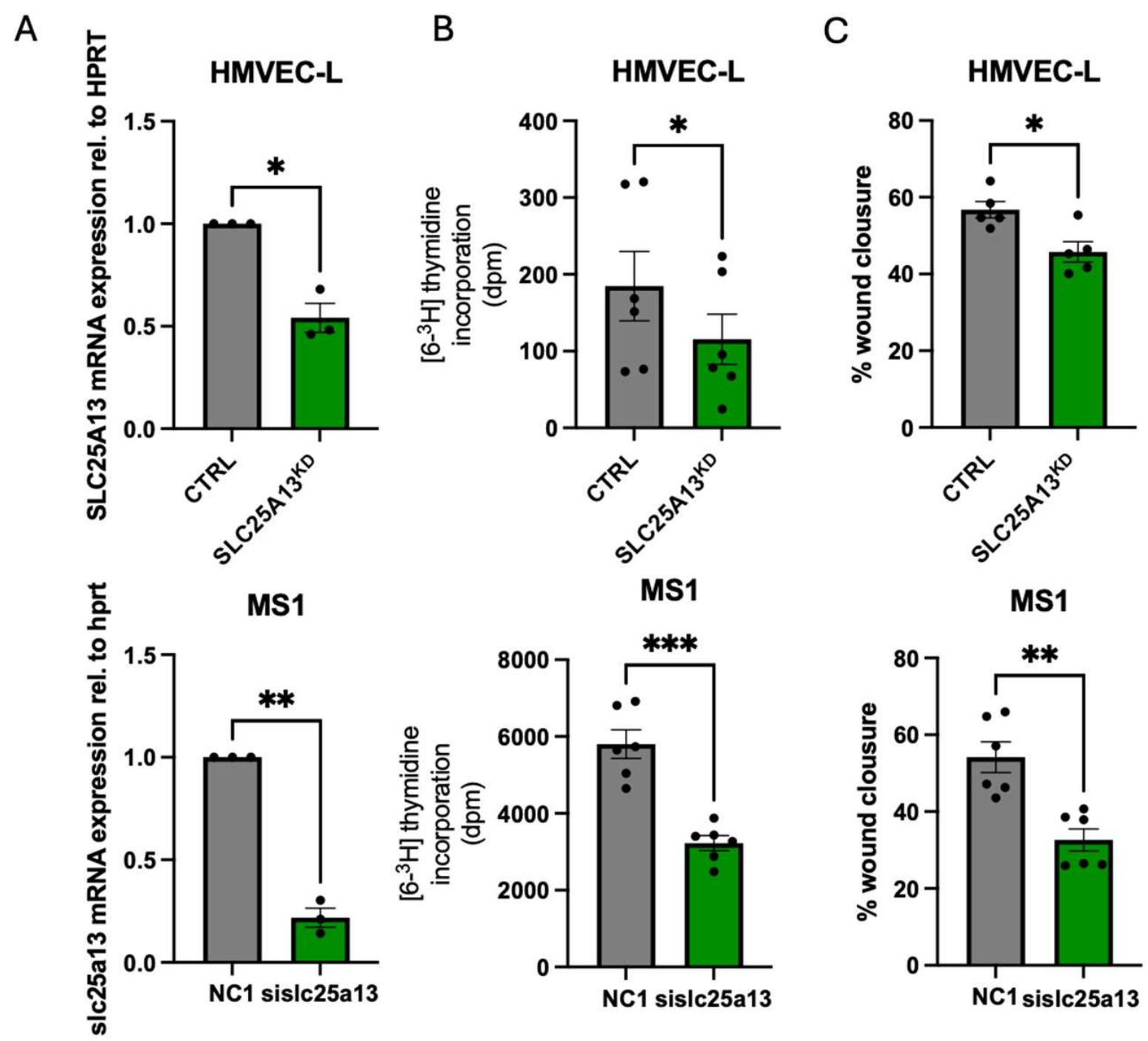

Figure RL16. A. mRNA expression (RT-qPCR) of SLC25A13/slc25a13 in CTRL (negative control) or SLC25A13^{KD}/sislc25a13 in HMVEC-L or MS1 cell lines (n=3). B. [6-^{3}H]-Thymidine incorporation into DNA in CTRL and SLC25A13^{KD} MS1 and HMVEC-L. C. Wound closure capacity (proxy of migration) of mitomycin-C treated CTRL and SLC25A13^{KD} MS1 and HMVECL. Data are means ± SEM; *p<0.05, **p<0.01, ***p<0.001 by two-tailed paired t-test (A, B, C).

Figure for reviewers removed

Figure for reviewers removed

4) Assuming that the modeling and thus the experiments that emerged from the modeling were not done across experimental conditions (i.e. states of high glucose or hypoxia) perhaps this could be added to the limitations.

Response: We thank the reviewer for these insightful suggestions. We acknowledge that both modeling and experimental validation were conducted under standard culture conditions. Exploring the shift in the role of predicted pathways and essential genes in various physiologically-relevant experimental conditions might help to further expand our understanding regarding the adaptability of endothelial cell proliferation to such stimuli. Genome-scale metabolic models are amenable to the integration of omics datasets representing various conditions (e.g. hypoxia) and further qualitative predictions. This indeed represents an important direction for future work. We have added this as a limitation under the "Limitations" section of our manuscript.

REFERENCES

Chrzanowska-Wodnicka M, Kraus AE, Gale D, White GC, 2nd, Vansluys J (2008) Defective angiogenesis, endothelial migration, proliferation, and MAPK signaling in Rap1b-deficient mice. *Blood* 111: 2647–2656

De Bock K, Georgiadou M, Schoors S, Kuchnio A, Wong BW, Cantelmo AR, Quaegebeur A, Ghesquiere B, Cauwenberghs S, Eelen G *et al* (2013) Role of PFKFB3-driven glycolysis in vessel sprouting. *Cell* 154: 651–663

Gariano RF, Gardner TW (2005) Retinal angiogenesis in development and disease. *Nature* 438: 960–966

Gutierrez-Aguilar M, Baines CP (2013) Physiological and pathological roles of mitochondrial SLC25 carriers. *Biochem J* 454: 371–386

Infantino V, Dituri F, Convertini P, Santarsiero A, Palmieri F, Todisco S, Mancarella S, Giannelli G, Iacobazzi V (2019) Epigenetic upregulation and functional role of the mitochondrial aspartate/glutamate carrier isoform 1 in hepatocellular carcinoma. *Biochim Biophys Acta Mol Basis Dis* 1865: 38–47

Lambrechts D, Wauters E, Boeckx B, Aibar S, Nittner D, Burton O, Bassez A, Decaluwe H, Pircher A, Van den Eynde K *et al* (2018) Phenotype molding of stromal cells in the lung tumor microenvironment. *Nat Med* 24: 1277–1289

Liu CH, Wang Z, Sun Y, Chen J (2017) Animal models of ocular angiogenesis: from development to pathologies. *FASEB J* 31: 4665–4681

Palmieri F (2004) The mitochondrial transporter family (SLC25): physiological and pathological implications. *Pflugers Arch* 447: 689–709

Palmieri L, Pardo B, Lasorsa FM, del Arco A, Kobayashi K, Iijima M, Runswick MJ, Walker JE, Saheki T, Satrustegui J *et al* (2001) Citrin and aralar1 are Ca(2+)-stimulated aspartate/glutamate transporters in mitochondria. *EMBO J* 20: 5060–5069

Vandekeere S, Dubois C, Kalucka J, Sullivan MR, Garcia-Caballero M, Goveia J, Chen R, Diehl FF, Bar-Lev L, Souffreau J *et al* (2018) Serine Synthesis via PHGDH Is Essential for Heme Production in Endothelial Cells. *Cell Metab* 28: 573–587 e513

Wimmer R, Cseh B, Maier B, Scherrer K, Baccarini M (2012) Angiogenic sprouting requires the fine tuning of endothelial cell cohesion by the Raf-1/Rok-alpha complex. *Dev Cell* 22: 158–171

Wu W, Liu S, Ren H, Rao Y, Nie J, Wei K, Jiang X (2025) Unveiling the oncogenic role of SLC25A13: a multi-omics pan-cancer analysis reveals its impact on glioma progression. *Cancer Cell Int* 25: 76

29th May 2026

Dear Prof. Carmeliet,

Thank you for submitting your revised manuscript to EMBO Molecular Medicine. We have now received the referees' reports. As you will see below, while Referees #1 and #3 are satisfied with the revisions, Referee #2 continues to raise substantial concerns.

We have carefully considered the reports and discussed the remaining issues both internally and with the referees. On this basis, we would like to invite a further revision of the manuscript. In particular, we encourage you to provide a more comprehensive discussion of the study's limitations and to moderate the conclusions where appropriate in light of the remaining concerns.

Please note that, at EMBO Press, we typically allow only one round of major revisions. Accordingly, this will represent the final opportunity to address the outstanding concerns raised during peer review.

Additionally, please address the editorial matters below:
- Please rename the conflict of interest to Disclosure and Competing Interests Statement
- The corresponding author's email should be provided on the title page of the manuscript.
- Author contributions should be removed from the manuscript. You will be asked to provide CRediT (Contributor Role Taxonomy) terms in the submission system instead.
- EMBO Press doesn't allow "Data not shown" or "unpublished data". All data should be presented in the main or EV figures.
- Author checklist: please use the template that you can download from our author guidelines.
- The funding information provided in the manuscript should match the information provided in the submission system. Currently, FWO (I002722N) is missing in the system; 1190922N (FWO) is missing in the Acknowledgements.
- Please check the callouts for all figures and figure panels: currently, Table EV1 and Table EV2 are not correct; "supplementary" nomenclature should not be used.
- Supplementary File S1 and Supplementary File S2 need to be updated to Dataset EV1 and Dataset EV2 in all places (source file names, legends in the file, titles in the system, manuscript callouts); the legends need to be removed from the manuscript and provided in each Excel file as a separate sheet/tab.
- Please provide The Paper Explained in the main manuscript file.
- The visual abstract should be uploaded as an individual png/tiff/jpeg file, 550px x 300 px.
- Please provide Source data for all figures/figure panels, as 1 zip file per figure.
- Supplementary figure legends should be Expanded View figure legends.
- Main and EV figures should be placed at the end, after the references.
- Please address the queries from our data editors:
1. Please define the annotated p values ****/***/**/* as well as provide the exact p-values for the same in the legend of figure EV3 D as appropriate.
2. Please note that the exact p values are not provided in the legends of figures 2A-E; 3D-G; 4A-J, EV2 A-C, F, I-L; EV4 B-D
3. Please note that information related to n is missing in the legends of figures 4D, EV2 F, G-J
4. For n=2 (Figure EV2D), please remove statistics and error bars, and only provide individual data points.

I look forward to seeing a revised form of your manuscript as soon as possible.

With kind regards,

Lise Roth

Lise Roth, Ph.D
Senior Editor
EMBO Molecular Medicine

***** Reviewer's comments *****

Referee #1 (Remarks for Author):

The authors have addressed all of my concerns and substantially improved the quality of their manuscript.

Referee #2 (Comments on Novelty/Model System for Author):

The authors showed that the mitochondrial glutamate-aspartate antiporter SLC25A13 coordinates glutamine catabolism primarily for cholesterol and nucleotide biosynthesis rather than energy production in endothelial cells, with its silencing impairing proliferation, migration, and pathological angiogenesis through preferential disruption of reductive carboxylation pathways-representing the first demonstration of glutamine-dependent cholesterol synthesis in any cell type and establishing SLC25A13 as a potential anti-angiogenic therapeutic target.

The paper's central assertion that SLC25A13 plays an endothelial-distinct metabolic role rests on literature comparisons rather than within-study parallel experiments in non-endothelial cells. This concern is amplified by established tissue distribution data showing citrin (SLC25A13) is 'predominantly expressed in non-excitable tissues: liver, kidney, heart, pancreas, suggesting broad rather than endothelial-specific function. Without performing the same silencing, 13C-glutamine tracing, cholesterol/mevalonate measurements, and proliferation assays in matched non-EC proliferating cell lines under identical culture conditions, it is difficult to exclude that the differences highlighted in Figures 3-4 reflect methodological or contextual variation rather than genuine EC-specific biology. This weakens the paper's therapeutic rationale, which depends on EC selectivity.

At least textual edits such as:
A within-study, head-to-head comparison of SLC25A13 dependence between ECs and non-EC proliferating cells under matched culture and silencing conditions was not performed, and therefore the EC-specificity of the observed biosynthetic coupling remains to be directly confirmed."

Replace "To our knowledge, the predominant SLC25A13-linked utilization of glutamine for cholesterol synthesis is a phenomenon that has not been documented in other cell types" with "To our knowledge, predominant SLC25A13-linked utilization of glutamine for cholesterol synthesis has not been reported in other cell types in prior literature; direct within-study comparisons to non-EC proliferating cells will be needed to establish EC-specificity."

Because SLC25A13 silencing alters cell-cycle distribution, the systematic cell-cycle composition shift may bias all bulk measurements used to construct the unified mechanistic model, not just any single assay. S-phase arrest (Figures EV2I-J) changes the relative abundance of cells with distinct metabolic demands, potentially producing pathway-level signals in Figure 3A-B and labeling shifts in Figures 3D-G and 4A-C that are partially secondary to composition rather than direct transporter function. This concern is supported by established evidence that cell cycle factors can explain up to 7% of gene expression variance in population-level data [1] and that cell-type proportion changes create systematic biases in bulk RNA-seq measurements [2]. Without synchronized populations, cell-cycle-matched comparisons, or single-cell resolution for at least one mechanistic readout, the paper-wide inference chain inherits this bias.

My suggestions are at First, to justify the current interpretation by noting that fractional isotopologue enrichments (used in Figures 3D-G and 4A) and rescue experiments (Figures 4E-F) are relatively robust to cell-cycle composition. For example, add: "Although SLC25A13 silencing leads to S-phase arrest (Figure EV2I-J), the fractional 13C-labeling patterns and metabolite rescue phenotypes reported here are consistent with direct metabolic effects, as fractional enrichment and functional rescue are less sensitive than absolute pool sizes to cell-cycle compositional shifts."

Next, acknowledge the limitation in the Limitations section: "SLC25A13 knockdown alters cell-cycle distribution, with an accumulation of cells in S-phase. Our bulk RNA-sequencing, steady-state metabolomics, and synthesis assays were performed on asynchronous populations, and therefore we cannot fully exclude that a portion of the observed transcriptional and metabolite-abundance changes reflects altered cell-cycle composition rather than direct metabolic consequences of SLC25A13 loss. Cell-cycle-matched or single-cell approaches will be required to fully deconvolve these contributions."

Finally, modify the main claim to reflect this nuanced perspective: Replace "Gene sets involved in cholesterol metabolism, purine catabolism, fatty acid synthesis, pyruvate metabolism, propanoate metabolism, glycerophospholipid metabolism were downregulated (mean statistic < 0) upon SLC25A13 silencing in ECs" with "Gene sets involved in cholesterol metabolism, purine catabolism, fatty acid synthesis, pyruvate metabolism, propanoate metabolism, and glycerophospholipid metabolism were downregulated upon SLC25A13 silencing in ECs; because SLC25A13 silencing also alters cell-cycle distribution (Figure EV2I-J), a portion of these bulk transcriptional changes may reflect compositional shifts in addition to direct SLC25A13-dependent regulation."

Referee #2 (Remarks for Author):

1. EC-GEM computational modeling predicts mitochondrial aspartate export and biomass synthesis to be essential in endothelial cells, but does not uniquely implicate SLC25A13 over SLC25A12. The computational analyses establish reaction-level essentiality of the glutamate/aspartate antiporter within the malate-aspartate shuttle, yet the underlying gene-reaction mapping includes both SLC25A12 and SLC25A13. Consequently, the model alone cannot distinguish which isoform is required. Both proteins function as glutamate/aspartate antiporters, and prior experimental work has shown that SLC25A12 can functionally compensate for SLC25A13 in hepatic cells, supporting their biochemical interchangeability despite tissue-specific expression patterns. Therefore, the claim that SLC25A13 itself is computationally predicted to be essential extends beyond what the modeling directly demonstrates. Rather, isoform-specific essentiality is resolved only through the subsequent experimental validation. Explicitly distinguishing reaction-level from gene-level inference would strengthen the interpretation.
2. SLC25A13 is reported to be essential for endothelial proliferation and angiogenesis both in vitro and in vivo, but the in vivo experiments do not establish endothelial-cell autonomy. Although Figure 2E demonstrates reduced neovascularization and Figure EV2L confirms decreased Slc25a13 transcript levels in sorted endothelial cells, the intravitreal siRNA delivery strategy is not endothelial specific and likely affects multiple ocular cell types, including retinal pigment epithelial cells, pericytes, and infiltrating immune cells. Established endothelial-targeted delivery systems can achieve high endothelial specificity with minimal off-target uptake, and specialized nanoparticle approaches have been developed for vascular-selective gene perturbation in vivo. Without endothelial-restricted genetic targeting using such validated approaches, the conclusion that endothelial Slc25a13 is intrinsically required for angiogenesis in vivo remains potentially confounded by contributions from non-endothelial cell populations.
3. The study lacks genetic rescue or orthogonal on-target validation to exclude off-target effects in the in vitro knockdown experiments. While Figure EV2B confirms efficient protein depletion and Figures 2A-D demonstrate substantial functional defects following SLC25A13 silencing, specificity is not established through standard orthogonal approaches such as rescue with an shRNA-resistant SLC25A13 construct, use of multiple independent siRNA/shRNA sequences producing convergent phenotypes, or CRISPR-mediated knockout followed by rescue. Off-target activity is widely recognized as a major limitation of RNAi-based experiments, and rescue experiments remain the most rigorous method for confirming on-target specificity. Similarly, use of multiple independent targeting sequences is considered best practice for RNAi validation. In the absence of these controls, the conclusion that SLC25A13 is strictly "essential" for endothelial proliferation and angiogenesis may be overstated.
4. SLC25A13 knockdown impairs endothelial wound closure and vessel sprouting, but it is unclear whether these phenotypes specifically reflect impaired migration independent of proliferation or drug-sensitivity effects. Both assays rely on mitomycin C to suppress proliferation, yet the study does not demonstrate equivalent cytostatic or cytotoxic effects across experimental groups. This is important because mitomycin C can itself alter migratory behavior in a context-dependent manner and exhibits marked variability in cellular sensitivity across different cell types. The modest increases in apoptosis and perturbations in S-phase progression observed in Figures EV2F-J could enhance differential sensitivity to mitomycin C or indirectly influence sprouting behavior independent of intrinsic motility. Without direct single-cell migration measurements, such as time-lapse tracking performed in the absence of cytostatic agents, or explicit controls demonstrating equivalent mitomycin C responses between conditions, the data do not conclusively establish a migration-specific defect (Figures 2C, 2D, EV2K, EV2F-J).
5. The conclusion that SLC25A13 silencing suppresses pathological angiogenesis in vivo is limited by the use of a single non-cell-type-specific siRNA without orthogonal validation. Although endothelial target engagement was demonstrated at the mRNA level (Figure EV2L), protein knockdown was not confirmed in vivo, and the intravitreal delivery strategy likely affects additional cell populations, including retinal pigment epithelial cells, pericytes, and immune cells. Consequently, the reduced neovascular area observed in Figure 2E cannot be definitively attributed to endothelial SLC25A13 depletion. Additional validation using independent siRNA sequences, rescue with an siRNA-resistant construct, or endothelial-restricted targeting strategies would substantially strengthen the causal interpretation. This issue is particularly important because siRNAs have previously been shown to suppress choroidal neovascularization through target-independent mechanisms such as TLR3 activation, with non-targeting siRNAs producing anti-angiogenic effects comparable to sequence-specific reagents. Furthermore, both siRNA seed effects and in vivo-jetPEI delivery systems can elicit immunostimulatory responses that may independently influence angiogenesis.
6. The study proposes that SLC25A13 preferentially regulates reductive glutamine metabolism, but the metabolic analyses do not directly quantify reductive versus oxidative fluxes. The conclusions are inferred primarily from endpoint fractional labeling patterns rather than from compartment-resolved ^13C metabolic flux analysis (MFA) or time-resolved tracing experiments capable of quantitatively distinguishing reductive and oxidative TCA cycle fluxes with statistical confidence. Fractional enrichment measurements alone can be influenced by metabolite pool sizes, reversible exchange reactions, compartmental mixing between cytosolic and mitochondrial isocitrate dehydrogenase activity, and differential metabolite turnover. Formal compartment-specific ^13C-MFA approaches have been developed specifically to resolve these limitations and provide quantitative flux estimates with confidence intervals. In this context, the unchanged ^14CO2 production observed in Figure EV3B further suggests that total oxidative metabolism may remain preserved while flux redistribution occurs within the network. Without formal flux modeling, the magnitude and specificity of the proposed reductive-carboxylation defect remain incompletely resolved.
7. The conclusion that SLC25A13 primarily coordinates glutamine utilization for cholesterol synthesis and biomass production is not directly demonstrated. Although the study shows reduced labeling of mevalonate from ^13C-glutamine (Figure 4C), it does not directly measure incorporation of glutamine-derived carbon into cholesterol itself, nor does it compare glutamine contribution against alternative substrates such as acetate or fatty acids. Consequently, the assertion that glutamine is the predominant carbon source for cholesterol synthesis remains inferential. Reduced mevalonate labeling could alternatively reflect

transcriptional downregulation of the mevalonate pathway, as suggested by the RNA-seq analyses (Figures 3A-B), rather than a direct limitation in glutamine carbon flux. While unchanged glucose-derived cholesterol labeling (Figure EV3E) argues against glucose as a major contributor, it does not distinguish glutamine from other established cholesterol precursors. Direct cholesterol isotopologue analysis following ^13C-glutamine tracing would therefore be required to substantiate the proposed substrate predominance.

8. The claim that SLC25A13 function is uncoupled from glycolytic flux and ATP production may be premature given the limited metabolic conditions tested. Measurements of glycolysis and ATP levels were restricted to basal steady-state conditions and bulk cellular readouts, without assessment of maximal glycolytic capacity, stress-induced metabolic demand, or compartment-specific ATP and redox dynamics. Because the malate-aspartate shuttle is particularly important for maintaining cytosolic NAD^+ regeneration under conditions of elevated glycolytic demand, defects may become apparent only during metabolic stress or increased energetic load. Standard glycolytic stress-test paradigms can distinguish basal glycolytic activity from maximal glycolytic reserve and often reveal latent metabolic constraints not evident at baseline. Likewise, compartment-targeted redox biosensors can detect subcellular perturbations in NADH/NAD^+ balance that are obscured in bulk measurements. Therefore, the unchanged basal metrics reported in Figures 4L-N may not fully exclude condition-dependent or spatially localized coupling between SLC25A13 function and energy metabolism.

Referee #3 (Remarks for Author):

My concerns/comments/questions were addressed.

EMM-2024-20849-V2 Rebuttal Letter

Referee #1 (Remarks for Author):

Comment: **The authors have addressed all of my concerns and substantially improved the quality of their manuscript.**

Referee #2 (Comments on Novelty/Model System for Author) :

Comment: **The authors showed that the mitochondrial glutamate-aspartate antiporter SLC25A13 coordinates glutamine catabolism primarily for cholesterol and nucleotide biosynthesis rather than energy production in endothelial cells, with its silencing impairing proliferation, migration, and pathological angiogenesis through preferential disruption of reductive carboxylation pathways-representing the first demonstration of glutamine-dependent cholesterol synthesis in any cell type and establishing SLC25A13 as a potential anti-angiogenic therapeutic target.**

The paper's central assertion that SLC25A13 plays an endothelial-distinct metabolic role rests on literature comparisons rather than within-study parallel experiments in non-endothelial cells. This concern is amplified by established tissue distribution data showing citrin (SLC25A13) is 'predominantly expressed in non-excitable tissues: liver, kidney, heart, pancreas, suggesting broad rather than endothelial-specific function. Without performing the same silencing, 13C-glutamine tracing, cholesterol/mevalonate measurements, and proliferation assays in matched non-EC proliferating cell lines under identical culture conditions, it is difficult to exclude that the differences highlighted in Figures 3-4 reflect methodological or contextual variation rather than genuine EC-specific biology. This weakens the paper's therapeutic rationale, which depends on EC selectivity.

At least textual edits such as:

A within-study, head-to-head comparison of SLC25A13 dependence between ECs and non-EC proliferating cells under matched culture and silencing conditions was not performed, and therefore the EC-specificity of the observed biosynthetic coupling remains to be directly confirmed."

Response: We thank the reviewer for this comment and agree that direct, head-to-head comparisons between ECs and other proliferating cell types under matched experimental conditions would be required to formally establish endothelial-cell specificity of SLC25A13-dependent metabolic phenotypes.

However, we would like to clarify that EC-specificity is not the primary claim of the present study. Rather, our objective was to define the role of SLC25A13 in endothelial cells and to identify the metabolic processes supported by this transporter in ECs. Our data demonstrate that SLC25A13 plays a critical role in endothelial metabolism, including the maintenance of

glutamine-derived cholesterol biosynthesis, but do not establish that this function is unique to ECs relative to other proliferating cell types.

Accordingly, we have revised the manuscript to avoid language that could be interpreted as claiming endothelial-cell specificity. Instead, we state that, to our knowledge, the prominent link between SLC25A13 activity and glutamine-supported cholesterol synthesis has not previously been reported in other cell types. Determining whether this metabolic coupling is unique to ECs or shared with additional proliferating cell populations will require direct comparative studies. These revisions are yellow highlighted on Pages 15, 17 and 18.

We also note that within ECs, our data identify a non-redundant requirement for SLC25A13 that is not compensated by the closely related glutamate-aspartate transporter SLC25A12, highlighting a specific role for SLC25A13 in endothelial metabolic regulation.

Comment: Replace "To our knowledge, the predominant SLC25A13-linked utilization of glutamine for cholesterol synthesis is a phenomenon that has not been documented in other cell types" with "To our knowledge, predominant SLC25A13-linked utilization of glutamine for cholesterol synthesis has not been reported in other cell types in prior literature; direct within-study comparisons to non-EC proliferating cells will be needed to establish EC-specificity."

Response: As per the reviewer's suggestion, we have made the corresponding change on Page 15.

Comment: Because SLC25A13 silencing alters cell-cycle distribution, the systematic cell-cycle composition shift may bias all bulk measurements used to construct the unified mechanistic model, not just any single assay. S-phase arrest (Figures EV2I-J) changes the relative abundance of cells with distinct metabolic demands, potentially producing pathway-level signals in Figure 3A-B and labeling shifts in Figures 3D-G and 4A-C that are partially secondary to composition rather than direct transporter function. This concern is supported by established evidence that cell cycle factors can explain up to 7% of gene expression variance in population-level data [1] and that cell-type proportion changes create systematic biases in bulk RNA-seq measurements [2]. Without synchronized populations, cell-cycle-matched comparisons, or single-cell resolution for at least one mechanistic readout, the paper-wide inference chain inherits this bias.

My suggestions are at First, to justify the current interpretation by noting that fractional isotopologue enrichments (used in Figures 3D-G and 4A) and rescue experiments (Figures 4E-F) are relatively robust to cell-cycle composition. For example, add: "Although SLC25A13 silencing leads to S-phase arrest (Figure EV2I-J), the fractional 13C-labeling patterns and metabolite rescue phenotypes reported here are consistent with direct metabolic effects, as fractional enrichment and functional rescue are less sensitive than absolute pool sizes to cell-cycle compositional shifts."

Response: As per the reviewer's suggestion, we have made the corresponding change on Page 9

Comment: **Next, acknowledge the limitation in the Limitations section: "SLC25A13 knockdown alters cell-cycle distribution, with an accumulation of cells in S-phase. Our bulk RNA-sequencing, steady-state metabolomics, and synthesis assays were performed on asynchronous populations, and therefore we cannot fully exclude that a portion of the observed transcriptional and metabolite-abundance changes reflects altered cell-cycle composition rather than direct metabolic consequences of SLC25A13 loss. Cell-cycle-matched or single-cell approaches will be required to fully deconvolve these contributions."**

Response: As per the reviewer's suggestion, we have made the corresponding change on Page 18.

Comment: **Finally, modify the main claim to reflect this nuanced perspective: Replace "Gene sets involved in cholesterol metabolism, purine catabolism, fatty acid synthesis, pyruvate metabolism, propanoate metabolism, glycerophospholipid metabolism were downregulated (mean statistic < 0) upon SLC25A13 silencing in ECs" with "Gene sets involved in cholesterol metabolism, purine catabolism, fatty acid synthesis, pyruvate metabolism, propanoate metabolism, and glycerophospholipid metabolism were downregulated upon SLC25A13 silencing in ECs; because SLC25A13 silencing also alters cell-cycle distribution (Figure EV2I-J), a portion of these bulk transcriptional changes may reflect compositional shifts in addition to direct SLC25A13-dependent regulation."**

Response: As per the reviewer's suggestion, we have made the corresponding change on Page 10

Referee #2 (Remarks for Author):

Comment: **1. EC-GEM computational modeling predicts mitochondrial aspartate export and biomass synthesis to be essential in endothelial cells, but does not uniquely implicate SLC25A13 over SLC25A12. The computational analyses establish reaction-level essentiality of the glutamate/aspartate antiporter within the malate-aspartate shuttle, yet the underlying gene-reaction mapping includes both SLC25A12 and SLC25A13. Consequently, the model alone cannot distinguish which isoform is required. Both proteins function as glutamate/aspartate antiporters, and prior experimental work has shown that SLC25A12 can functionally compensate for SLC25A13 in hepatic cells, supporting their biochemical interchangeability despite tissue-specific expression patterns. Therefore, the claim that SLC25A13 itself is computationally predicted to be essential extends beyond what the modeling directly demonstrates. Rather, isoform-specific essentiality is resolved only through the subsequent experimental validation. Explicitly distinguishing reaction-level from gene-level inference would strengthen the interpretation.**

Response: We thank the reviewer for this suggestion. Although the EC-GEM showcases reaction-level essentiality, it is important to note that its reaction knockout analyses could appropriately predict the essentiality of the mitochondrial glutamate-aspartate carrier reaction for biomass synthesis, which is a new observation in ECs from a metabolic perspective. This triggered us to focus on the SLC25A12 / SLC25A13 genes and perform gene-

level confirmatory experimental perturbations. From our *in vitro* results, it is clear that SLC25A13^{KD} leads to a defective proliferation phenotype, which cannot be compensated for by SLC25A12. We additionally did not observe a reduction in proliferation upon SLC25A12^{KD}, suggesting that SLC23A13 is uniquely essential for EC proliferation (Fig. EV2C). However, to clarify our point better, we have made textual modifications to explicitly distinguish the gene-level inference with model-based reaction-level inference. These are yellow highlighted on Pages 5, 8, 9 and 13.

Comment: 2. SLC25A13 is reported to be essential for endothelial proliferation and angiogenesis both in vitro and in vivo, but the in vivo experiments do not establish endothelial-cell autonomy. Although Figure 2E demonstrates reduced neovascularization and Figure EV2L confirms decreased Slc25a13 transcript levels in sorted endothelial cells, the intravitreal siRNA delivery strategy is not endothelial specific and likely affects multiple ocular cell types, including retinal pigment epithelial cells, pericytes, and infiltrating immune cells. Established endothelial-targeted delivery systems can achieve high endothelial specificity with minimal off-target uptake, and specialized nanoparticle approaches have been developed for vascular-selective gene perturbation in vivo. Without endothelial-restricted genetic targeting using such validated approaches, the conclusion that endothelial Slc25a13 is intrinsically required for angiogenesis in vivo remains potentially confounded by contributions from non-endothelial cell populations.

Response: We thank the reviewer for this point. We agree that intravitreal siRNA delivery does not provide endothelial-specific genetic targeting and may affect multiple ocular cell types, including retinal pigment epithelial cells, pericytes, and infiltrating immune cells. We acknowledge that, as a result, these in vivo experiments do not allow us to formally conclude endothelial-cell autonomy of the observed phenotype.

To partially address this limitation, we confirmed reduced Slc25a13 expression in sorted endothelial cells from treated retinas (Figure EV2L), indicating that endothelial cells are affected by the knockdown. Nevertheless, we agree that contributions from non-endothelial cell populations cannot be excluded. The previous manuscript already acknowledged this limitation. Specifically, in our previous version, we stated: *'However, since the silencing in this model is not EC-specific, effects on other cell types could also contribute to this in vivo vascular phenotype'.* We have further revised the text to avoid claiming an endothelial-cell-intrinsic requirement in vivo and instead describe the in vivo findings as consistent with a role for SLC25A13 in pathological neovascularization with endothelial involvement.

This text referred to a figure for reviewers that was removed because it displayed unpublished data. The text also referred to this data and was consequently removed.

Although these findings were obtained in a distinct experimental context and do not directly address the retinal neovascularization model, they provide independent support for a functional role of endothelial SLC2513 in vivo.

Figures for reviewers removed

Comment: **3. The study lacks genetic rescue or orthogonal on-target validation to exclude off-target effects in the in vitro knockdown experiments. While Figure EV2B confirms efficient protein depletion and Figures 2A-D demonstrate substantial functional defects following SLC25A13 silencing, specificity is not established through standard orthogonal approaches such as rescue with an shRNA-resistant SLC25A13 construct, use of multiple independent siRNA/shRNA sequences producing convergent phenotypes, or CRISPR-**

mediated knockout followed by rescue. Off-target activity is widely recognized as a major limitation of RNAi-based experiments, and rescue experiments remain the most rigorous method for confirming on-target specificity. Similarly, use of multiple independent targeting sequences is considered best practice for RNAi validation. In the absence of these controls, the conclusion that SLC25A13 is strictly "essential" for endothelial proliferation and angiogenesis may be overstated.

Response: We agree that rescue experiments, the use of multiple independent targeting sequences, or orthogonal genetic approaches would further strengthen the demonstration of on-target specificity. In the present work, our conclusions are based on the convergence of several independent observations. First, SLC25A13 knockdown resulted in robust depletion of the target protein (Figure EV2B). Second, the observed phenotypes were consistent across multiple functional, metabolic, and biochemical readouts. Third, several of the metabolic perturbations predicted by the EC-GEM were independently supported by metabolite rescue experiments and isotope-labeling analyses. While these findings support the interpretation that the observed phenotypes are attributable to SLC25A13 depletion, we agree that they do not formally exclude potential off-target contributions.

Accordingly, we have revised the manuscript to avoid overstatement and to clarify in the “Limitations” that additional orthogonal genetic approaches, including rescue experiments, would further strengthen the causal attribution of the observed phenotypes to SLC25A13. These revisions are yellow highlighted on Pages 3, 8, 14 and 17.

Comment: **4. SLC25A13 knockdown impairs endothelial wound closure and vessel sprouting, but it is unclear whether these phenotypes specifically reflect impaired migration independent of proliferation or drug-sensitivity effects. Both assays rely on mitomycin C to suppress proliferation, yet the study does not demonstrate equivalent cytostatic or cytotoxic effects across experimental groups. This is important because mitomycin C can itself alter migratory behavior in a context-dependent manner and exhibits marked variability in cellular sensitivity across different cell types. The modest increases in apoptosis and perturbations in S-phase progression observed in Figures EV2F-J could enhance differential sensitivity to mitomycin C or indirectly influence sprouting behavior independent of intrinsic motility. Without direct single-cell migration measurements, such as time-lapse tracking performed in the absence of cytostatic agents, or explicit controls demonstrating equivalent mitomycin C responses between conditions, the data do not conclusively establish a migration-specific defect (Figures 2C, 2D, EV2K, EV2F-J).**

Response: We thank the reviewer for pointing this out. We agree that wound-healing and sprouting assays do not exclusively measure cell migration and may also be influenced by differential responses to mitomycin C. Our intention was to evaluate endothelial angiogenic behavior under conditions in which proliferation was minimized by mitomycin C treatment; however, we acknowledge that these experiments do not formally distinguish migration-specific effects from other cellular processes. While we cannot exclude a contribution of altered mitomycin C sensitivity or changes in proliferation and survival, the relatively modest effects observed in apoptosis and cell-cycle distribution compared with the magnitude of the wound-closure and sprouting phenotypes are consistent with a predominant effect on overall angiogenic capacity rather than proliferation alone.

Comment: **5. The conclusion that SLC25A13 silencing suppresses pathological angiogenesis in vivo is limited by the use of a single non-cell-type-specific siRNA without orthogonal validation. Although endothelial target engagement was demonstrated at the mRNA level (Figure EV2L), protein knockdown was not confirmed in vivo, and the intravitreal delivery strategy likely affects additional cell populations, including retinal pigment epithelial cells, pericytes, and immune cells. Consequently, the reduced neovascular area observed in Figure 2E cannot be definitively attributed to endothelial SLC25A13 depletion. Additional validation using independent siRNA sequences, rescue with an siRNA-resistant construct, or endothelial-restricted targeting strategies would substantially strengthen the causal interpretation. This issue is particularly important because siRNAs have previously been shown to suppress choroidal neovascularization through target-independent mechanisms such as TLR3 activation, with non-targeting siRNAs producing anti-angiogenic effects comparable to sequence-specific reagents. Furthermore, both siRNA seed effects and in vivo-jetPEI delivery systems can elicit immunostimulatory responses that may independently influence angiogenesis.**

Response: We acknowledge that in vivo protein knockdown was not assessed and that intravitreal administration of the siRNA/in vivo-jetPEI complex may affect multiple retinal cell types. Therefore, we cannot formally exclude contributions from non-endothelial cells to the observed phenotype.

Regarding potential target-independent effects of siRNA delivery, all in vivo experiments included a non-targeting control siRNA formulated and administered under identical conditions. Thus, both experimental groups were equally exposed to the siRNA molecule and the delivery vehicle, controlling for nonspecific effects associated with siRNA administration or in vivo-jetPEI treatment. The reduction in neovascularization was observed specifically in eyes receiving the SLC25A13-targeting siRNA relative to the non-targeting control. Nevertheless, we agree that additional validation using independent siRNA sequences or rescue approaches would further strengthen the causal interpretation of the findings. To reflect these limitations, we have revised the manuscript and moderated our conclusions.

Comment: **6. The study proposes that SLC25A13 preferentially regulates reductive glutamine metabolism, but the metabolic analyses do not directly quantify reductive versus oxidative fluxes. The conclusions are inferred primarily from endpoint fractional labeling patterns rather than from compartment-resolved ^13C metabolic flux analysis (MFA) or time-resolved tracing experiments capable of quantitatively distinguishing reductive and oxidative TCA cycle fluxes with statistical confidence. Fractional enrichment measurements alone can be influenced by metabolite pool sizes, reversible exchange reactions, compartmental mixing between cytosolic and mitochondrial isocitrate dehydrogenase activity, and differential metabolite turnover. Formal compartment-specific ^13C-MFA approaches have been developed specifically to resolve these limitations and provide quantitative flux estimates with confidence intervals. In this context, the unchanged ^14CO2 production observed in Figure EV3B further suggests that total oxidative metabolism may remain preserved while flux redistribution occurs within the network.**

Without formal flux modeling, the magnitude and specificity of the proposed reductive-carboxylation defect remain incompletely resolved.

Response: We agree with the reviewer that fractional labelling patterns cannot alone provide quantitative changes regarding reductive vs. oxidative TCA fluxes and indeed requires compartment-resolved quantitative partitioning, which compartment-specific ^{13}C-MFA or time-resolved tracing experiments can provide. However, we observe consistent patterns in fractional labelling of citrate and downstream metabolites that show a decrease in reductive carboxylation upon SLC25A13^{KD} relative to glutamine oxidation. These labelling changes are consistent with the reduction seen in mevalonate / cholesterol abundance (as measured by relative and absolute measurements; Figs. 4B, 4C, 4D). Collectively, our evidence supports a specific change in the balance of glutamine-derived carbon utilization toward oxidative TCA entry and away from reductive carboxylation in SLC25A13-perturbed ECs. The unchanged total $^{14}CO_2$ release (Fig EV3B) argues against suppression of oxidative metabolism and is therefore consistent with flux redistribution (between reductive and oxidative glutamine metabolism) rather than overall reduction in TCA activity. Additionally, flux predictions from the EC-GEM and our perturbation experiments produce congruent growth-associated changes in metabolic outputs (reduction in amino acids, nucleotides and cholesterol) supporting a functional consequence of the observed metabolic shifts. To summarize, SLC25A13 perturbation leads to reproducible changes in glutamine-derived labeling and functional metabolic outcomes that are consistent with altered routing through reductive carboxylation, but formal compartment-resolved flux quantitation remains an important next step. This limitation has been added to Manuscript page 18 (highlighted in yellow).

Comment: **7. The conclusion that SLC25A13 primarily coordinates glutamine utilization for cholesterol synthesis and biomass production is not directly demonstrated. Although the study shows reduced labeling of mevalonate from ^13C-glutamine (Figure 4C), it does not directly measure incorporation of glutamine-derived carbon into cholesterol itself, nor does it compare glutamine contribution against alternative substrates such as acetate or fatty acids. Consequently, the assertion that glutamine is the predominant carbon source for cholesterol synthesis remains inferential. Reduced mevalonate labeling could alternatively reflect transcriptional downregulation of the mevalonate pathway, as suggested by the RNA-seq analyses (Figures 3A-B), rather than a direct limitation in glutamine carbon flux. While unchanged glucose-derived cholesterol labeling (Figure EV3E) argues against glucose as a major contributor, it does not distinguish glutamine from other established cholesterol precursors. Direct cholesterol isotopologue analysis following ^13C-glutamine tracing would therefore be required to substantiate the proposed substrate predominance.**

Response: We thank the reviewer for pointing out the possibilities of metabolic and non-metabolic mechanisms that can link glutamine utilization with biomass production. Although mevalonate is a potential precursor for cholesterol synthesis, we agree that reduced mevalonate labeling does not directly demonstrate glutamine to be a predominant carbon source for cholesterol synthesis unless labelled cholesterol is also measured. However, it is important to note that labeled mevalonate, relative abundance of total mevalonate and total cholesterol abundance were substantially reduced upon SLC25A13^{KD} (Fig. 4B-D), suggesting an overall congruent link of cholesterol biosynthesis with glutamine utilization. At the same time, RNA-seq data analyses show a downregulation of squalene and cholesterol pathway

genesets (Fig. 3A) and could provide an alternative non-metabolic, regulatory explanation for the reduction of mevalonate. Based on both observations, we believe that our data supports a dual mechanism of action where SLC25A13 perturbation: (i) metabolically reduces glutamine reductive carboxylation and thus cytosolic citrate/acetyl-CoA supply for mevalonate and cholesterol biosynthesis and (ii) transcriptionally downregulates the mevalonate pathway. This is also supported by the reductions observed in total abundance of cholesterol upon SLC25A13 perturbations and rescue experiments using mevalonate (Fig. 4E).

Based on previous literature that tested SLC25A13's link with glycolysis/glucose oxidation in proliferating cancer cells (Rabinovich *et al.,* 2020; Alkan *et al.*, 2018 and others), the EC-GEM's flux predictions of glutamine being catabolized into the TCA via mitochondrial glutamate-aspartate antiporter and essential role of mitochondrial glutamate-aspartate antiporter reaction via reaction knockout analyses, we prioritized ^{13}C glucose and ^{13}C glutamine labeling under SLC25A13 perturbations. Additionally, emerging evidence indicates that glutamine is metabolically essential as a carbon source for cholesterol synthesis in proliferating cell types *in vitro* and can also transcriptionally control the mevalonate pathway (Garcia *et al.* 2024). This result correlates with our metabolic observation of glutamine utilization for cholesterol synthesis and transcriptomics observations of downregulation of squalene and cholesterol synthesis. To answer the reviewer's concern, we have highlighted these possibilities in Page 16 of the Discussion section of the revised manuscript.

Comment: **8. The claim that SLC25A13 function is uncoupled from glycolytic flux and ATP production may be premature given the limited metabolic conditions tested. Measurements of glycolysis and ATP levels were restricted to basal steady-state conditions and bulk cellular readouts, without assessment of maximal glycolytic capacity, stress-induced metabolic demand, or compartment-specific ATP and redox dynamics. Because the malate-aspartate shuttle is particularly important for maintaining cytosolic NAD^+ regeneration under conditions of elevated glycolytic demand, defects may become apparent only during metabolic stress or increased energetic load. Standard glycolytic stress-test paradigms can distinguish basal glycolytic activity from maximal glycolytic reserve and often reveal latent metabolic constraints not evident at baseline. Likewise, compartment-targeted redox biosensors can detect subcellular perturbations in NADH/NAD^+ balance that are obscured in bulk measurements. Therefore, the unchanged basal metrics reported in Figures 4L-N may not fully exclude condition-dependent or spatially localized coupling between SLC25A13 function and energy metabolism.**

Response: We thank the reviewer for this important suggestion. We have performed Seahorse XF assays measuring oxygen consumption rate (OCR) under basal and oligomycin-treated conditions, which allowed us to calculate ATP-linked OCR (OCR_ATP), the mitochondrial respiration that drives ATP synthesis. Our data show that $SLC25A13^{KD}$ cells maintain comparable OCR_ATP relative to control (Fig. 4N), supporting decoupling from mitochondrial ATP production under basal conditions. Although we acknowledge that we did not measure ECAR (glycolysis) or assess maximal glycolytic capacity via a full glycolytic stress test, we measured glycolytic flux (Fig. 4O) in basal conditions and we did not observe any change upon $SLC25A13^{KD}$. Additionally, we did not assess compartment-specific $NADH/NAD^+$ dynamics under glycolytic stress conditions. We acknowledge this in the Limitations section

of Manuscript Page 18 (highlighted in yellow). We have thus reframed our language in the revised manuscript to specify that SLC25A13 function is uncoupled from glycolytic flux and ATP production under basal conditions, while acknowledging that condition-dependent or spatially localized coupling may emerge under metabolic stress.

Referee #3 (Remarks for Author):

Comment: **My concerns/comments/questions were addressed.**

24th Jul 2026

Dear Prof. Carmeliet,

Thank you for submitting your revised files. I am pleased to let you know that we will be able to accept your manuscript once the following editorial comments are addressed:

1/ Manuscript text:
- Please remove the highlighted font, and only keep in track changes any new modification.
- I would suggest changing the title to avoid "an overlooked player" and emphasize the role of SLC25A13, for instance "Endothelial SLC25A13 links glutamine metabolism to angiogenesis".
- Methods:
o Patient material: please include a statement confirming that the experiments conformed to the principles set out in the WMA Declaration of Helsinki and the Department of Health and Human Services Belmont Reports.
o Cell lines: please indicate their origine, and whether they were authenticated and tested for mycoplasma contamination.
o Statistics: please add a statement on blinding, randomization and inclusion/exclusion criteria.
- Data Availability Section: please provide a URL for E-MTAB-14591 dataset. Please deposit the raw and processed metabolomics data, together with appropriate sample metadata, in a public metabolomics repository (e.g. MetaboLights or Metabolomics Workbench) and provide the accession number in the manuscript. Please ensure that all custom scripts used for model simulations, downstream analyses and figure generation, together with the exact version of the EC-GEM model (if modified from the published version), are made publicly available in an appropriate repository to enable full reproducibility.

2/ Checklist:
Please fill in:
- Cell materials, subsections primary cultures and authentication/mycoplasma contamination.
- Experimental study design and statistics, all subsections.
- Ethics, subsections on human participants / details of authority granting ethics approval, as the information is provided in the manuscript. Please also fill in the part on informed consent and Helsinki Declaration.
- Data Availability, subsections on primary datasets and computational models.

3/ I introduced minor edits in your Paper Explained, please let me know if you agree or amend as you see fit:

Problem
Abnormal angiogenesis underlies several major diseases, including cancer and eye disorders, making EC metabolism an attractive therapeutic target.
Results
We identified the glutamate-aspartate antiporter SLC25A13, previously overlooked in endothelial cells (ECs), as a key regulator of EC metabolism. SLC25A13 coordinates glutamine utilization with the tricarboxylic acid cycle to support the synthesis of biomass precursors, particularly those of the mevalonate pathway.
Our findings suggest that SLC25A13 plays a distinct metabolic role in ECs compared with other proliferating cell types. Whereas previous studies have primarily linked SLC25A13 to coupling glycolysis with oxidative phosphorylation, our data indicate that in ECs it is essential for directing glutamine utilization towards the synthesis of biomass precursors, including mevalonate and cholesterol, highlighting a metabolic adaptation that appears to be particularly important during angiogenic proliferation. Accordingly, silencing SLC25A13 in ECs reduced angiogenesis and cell proliferation in vitro, as well as in a choroidal neovascularization mouse model in vivo.
Impact
Our findings reveal that silencing SLC25A13 significantly impairs angiogenesis and cell proliferation, supporting its further evaluation as a potential anti-angiogenic therapeutic target, particularly in diseases characterized by aberrant vascularization and pathological cell proliferation.

4/ As part of the EMBO Publications transparent editorial process initiative (see our Editorial at http://embomolmed.embopress.org/content/2/9/329), EMBO Molecular Medicine will publish online a Review Process File (RPF) to accompany accepted manuscripts.
This file will be published in conjunction with your paper and will include the anonymous referee reports, your point-by-point response and all pertinent correspondence relating to the manuscript. Let us know whether you agree with the publication of the RPF and as here, if you want to remove or not any figures from it prior to publication.
Please note that the Authors checklist will be published at the end of the RPF.

I look forward to receiving your revised manuscript.

Yours sincerely,

Lise Roth

Lise Roth, PhD
Senior Editor
EMBO Molecular Medicine

x

The authors addressed the remaining editorial issues.

28th Aug 2026

Dear Prof. Carmeliet,

Thank you for submitting your revised files. I am pleased to inform you that your manuscript is accepted for publication and is now being sent to our publisher to be included in the next available issue of EMBO Molecular Medicine.

Your manuscript will be processed for publication by EMBO Press. It will be copy edited and you will receive page proofs prior to publication. Please note that you will be contacted by Springer Nature Author Services to complete licensing and payment information.

You may qualify for financial assistance for your publication charges - either via a Springer Nature fully open access agreement or an EMBO initiative. Check your eligibility: https://link.springer.com/journal/44321/how-to-publish-with-us

Should you be planning a Press Release on your article, please notify the production team via this form in order to coordinate publication and release dates: https://forms.cloud.microsoft/e/eH4A8JVP6g

If you have any questions, please do not hesitate to contact the Editorial Office. Thank you for your contribution to EMBO Molecular Medicine.

Yours sincerely,

Lise

Lise Roth, Ph.D
Senior Editor
EMBO Molecular Medicine

>>> Please note that it is EMBO Molecular Medicine policy for the transcript of the editorial process (containing referee reports and your response letter) to be published as an online supplement to each paper. If you do NOT want this, you will need to inform the Editorial Office via email immediately. More information is available here: https://link.springer.com/partners/embo-press/editorial-policies#Peer%20review

EMBO Press Author Check

Corresponding Author Name: Prof. Peter Carmeliet	**USEFUL LINKS FOR COMPLETING THIS FORM**
Journal Submitted to: EMBO Molecular Medicine	The EMBO Journal - Author Guidelines
Manuscript Number: EMM-2024-20849-V2	EMBO Reports - Author Guidelines
	Molecular Systems Biology - Author Guidelines
	EMBO Molecular Medicine - Author Guidelines

Reporting Checklist for Life Sci

This checklist is adapted from Materials Design Analysis Reporting (MDAR) Checklist for Authors. MDAR establishes a minimum set of requirements in transparent reporting in th

Please note that a copy of this checklist will be published alongside y

Abridged guidelines for figures

1. Data

The data shown in figures should

- → the data were obtained and processed according to the field's best practice and are presented to reflect the results of the experiments in an accurate and unbiased m
- → ideally, figure panels should include only measurements that are directly comparable to each other and obtained with the same assay.
- → plots include clearly labeled error bars for independent experiments and sample sizes. Unless justified, error bars should not be shown for technical replicates.
- → if n<5, the individual data points from each experiment should be plotted. Any statistical test employed should be justified.
- → Source Data should be included to report the data underlying figures according to the guidelines set out in the authorship guidelines on Data Presentation.

2. Captions

Each figure caption should contain the following information, for each panel where they are relevant:

- → a specification of the experimental system investigated (eg cell line, species name).
- → the assay(s) and method(s) used to carry out the reported observations and measurements.
- → an explicit mention of the biological and chemical entity(ies) that are being measured.
- → an explicit mention of the biological and chemical entity(ies) that are altered/varied/perturbed in a controlled manner.
- → the exact sample size (n) for each experimental group/condition, given as a number, not a range;
- → a description of the sample collection allowing the reader to understand whether the samples represent technical or biological replicates (including how many animals
- → a statement of how many times the experiment shown was independently replicated in the laboratory.
- → definitions of statistical methods and measures:
  - common tests, such as t-test (please specify whether paired vs. unpaired), simple χ2 tests, Wilcoxon and Mann-Whitney tests, can be unambiguously identified by
  - are tests one-sided or two-sided?
  - are there adjustments for multiple comparisons?
  - exact statistical test results, e.g., P values = x but not P values < x;
  - definition of 'center values' as median or average;
  - definition of error bars as s.d. or s.e.m.

Please complete ALL of the questions below. Select "Not Applicable" only when the requested information is not relevant for your study.

Materials

	Information included in the manuscript?	In which section is the information available?
**Newly Created**	**Information included in the manuscript?**	**In which section is the information available?**
New materials and	Not Applicable	
**Antibodies**	**Information included in the manuscript?**	**In which section is the information available?**
For antibodies provide	Yes	Reagents and Tools Table, Materials and Methods
**DNA and RNA**	**Information included in the manuscript?**	**In which section is the information available?**
**Short novel DNA or**	Yes	Materials and Methods
**Cell materials**	**Information included in the manuscript?**	**In which section is the information available?**
**Cell lines: Provide**	Yes	Materials and Methods
**Primary cultures:**	Not Applicable	/
Report if the cell lines were recently authenticated (e.g., by STR profiling) and tested for mycoplasma contamination.	Not Applicable	/
**Experimental animals**	**Information included in the manuscript?**	**In which section is the information available? (Reagents and Tools Table, Materials and Methods, Figures, Data Availability Section)**
**Laboratory animals or Model organisms: Provide species, strain, sex, age, genetic modification status. Provide accession number in repository OR supplier name, catalog number, clone number, OR RRID.**	Yes	Materials and Methods
**Animal observed in or captured from the field: Provide species, sex, and age where possible**	Not Applicable	/
Please detail housing and husbandry conditions.	Yes	Materials and Methods
**Plants and microbes**	**Information included in the manuscript?**	**In which section is the information available? (Reagents and Tools Table, Materials and Methods, Figures, Data Availability Section)**
**Plants: provide species and strain, ecotype and cultivar where relevant, unique accession number if available, and source (including location for collected wild specimens).**	Not Applicable	/
**Microbes: provide species and strain, unique accession number if available, and source**	Not Applicable	/
**Human research participants**	**Information included in the manuscript?**	**In which section is the information available? (Reagents and Tools Table, Materials and Methods, Figures, Data Availability Section)**
If collected and within the bounds of privacy constraints report on age, sex and gender or ethnicity for all study participants.	Not Applicable	
**Core facilities**	**Information included in the manuscript?**	**In which section is the information available? (Reagents and Tools Table, Materials and Methods, Figures, Data Availability Section)**
If your work benefited from core facilities, was their service mentioned in the acknowledgments section?	Yes	Acknowledgements section

Design

	Information included in the manuscript?	In which section is the information available?
**Study protocol**	**Information included in the manuscript?**	**In which section is the information available? (Reagents and Tools Table, Materials and Methods, Figures, Data Availability Section)**
If study protocol has been pre-registered, provide DOI in the manuscript. For clinical trials, provide the trial registration number OR cite DOI.	Not Applicable	
Report the clinical trial registration number (at ClinicalTrials.gov or equivalent), where applicable.	Not Applicable	
**Laboratory protocol**	**Information included in the manuscript?**	**In which section is the information available? (Reagents and Tools Table, Materials and Methods, Figures, Data Availability Section)**
Provide DOI OR other citation details if external detailed step-by-step protocolsare available.	Yes	Materials and Methods
**Experimental study design and statistics**	**Information included in the manuscript?**	**In which section is the information available? (Reagents and Tools Table, Materials and Methods, Figures, Data Availability Section)**
Include a statement about sample size estimate even if no statistical methods were used.	Yes	Materials and Methods
Were any steps taken to minimize the effects of subjective bias when allocating animals/samples to treatment (e.g. randomization procedure)? If yes, have they been described?	Not Applicable	/
Include a statement about blinding even if no blinding was done.	Not Applicable	/
Describe inclusion/exclusion criteria if samples or animals were excluded from the analysis. Were the criteria pre-established?<br><br>If sample or data points were omitted from analysis, report if this was due to attrition or intentional exclusion and provide justification.	Not Applicable	/

	A	B	C	D	E	F	G
84			For every figure, are statistical tests justified as appropriate? Do the data meet the assumptions of the tests (e.g., normal distribution)? Describe any methods used to assess it. Is there an estimate of variation within each group of data? Is the variance similar between the groups that are being statistically compared?	Yes	Figures		
85							
86			**Sample definition and in-laboratory replication**	**Information included in the manuscript?**	**In which section is the information available? (Reagents and Tools Table, Materials and Methods, Figures, Data Availability Section)**		
87			In the figure legends: state number of times the experiment was replicated in laboratory.	Yes	Figures		
88			In the figure legends: define whether data describe technical or biological replicates.	Yes	Figures		
89							
90		**Ethics**					
91			**Ethics**	**Information included in the manuscript?**	**In which section is the information available? (Reagents and Tools Table, Materials and Methods, Figures, Data Availability Section)**		
92			Studies involving human participants: State details of authority granting ethics approval (IRB or equivalent committee(s), provide reference number for approval.	Not Applicable	/		
93			Studies involving human participants: Include a statement confirming that informed consent was obtained from all subjects and that the experiments conformed to the principles set out in the WMA Declaration of Helsinki and the Department of Health and Human Services Belmont Report.	Not Applicable	/		
94			Studies involving human participants: For publication of patient photos, include a statement confirming that consent to publish was obtained.	Not Applicable	/		
95			Studies involving experimental animals: State details of authority granting ethics approval (IRB or equivalent committee(s), provide reference number for approval. Include a statement of compliance with ethical regulations.	Yes	Materials and Methods		
96			Studies involving specimen and field samples: State if relevant permitsobtained, provide details of authority approving study; if none were required, explain why.	Not Applicable	/		
97							
98			**Dual Use Research of Concern (DURC)**	**Information included in the manuscript?**	**In which section is the information available? (Reagents and Tools Table, Materials and Methods, Figures, Data Availability Section)**		
99			Could your study fall under dual use research restrictions? Please check biosecurity documents and list of **select agents and toxins** (CDC): https://www.selectagents.gov/sat/list.htm	Not Applicable	/		
100			If you used a select agent, is the security level of the lab appropriate and reported in the manuscript?	Not Applicable	/		
101			If a study is subject to dual use research of concern regulations, is the name of the authority granting approval and reference number for the regulatory approval provided in the manuscript?	Not Applicable	/		
102							
103		**Reporting**					
104			The MDAR framework recommends adoption of discipline-specific guidelines, established and endorsed through community initiatives. Journals have their own policy about requiring specific guidelines and recommendations to complement MDAR.				
105			**Adherence to community standards**	**Information included in the manuscript?**	**In which section is the information available? (Reagents and Tools Table, Materials and Methods, Figures, Data Availability Section)**		
106			State if relevant guidelines or checklists (e.g., ICMJE, MIBBI, ARRIVE, PRISMA) have been followed or provided.	Not Applicable	/		
107			For tumor marker prognostic studies, we recommend that you follow the REMARK reporting guidelines (see link list at top right). See author guidelines, under 'Reporting Guidelines'. Please confirm you have followed these guidelines.	Not Applicable	/		
108			For phase II and III randomized controlled trials, please refer to the CONSORTflow diagram (see link list at top right) and submit the CONSORT checklist (see link list at top right) with your submission. See author guidelines, under 'Reporting Guidelines'. Please confirm you have submitted this list.	Not Applicable	/		
109							
110		**Data Availability**					
111			**Data availability**	**Information included in the manuscript?**	**In which section is the information available? (Reagents and Tools Table, Materials and Methods, Figures, Data Availability Section)**		
112			Have primary datasets been deposited according to the journal's guidelines (see 'Data Deposition' section) and the respective accession numbers provided in the Data Availability Section?	Not Applicable			
113			Were human clinical and genomic datasets deposited in a public access-controlled repository in accordance to ethical obligations to the patients and to the applicable consent agreement?	Not Applicable			
114			Are computational models that are central and integral to a study available without restrictions in a machine-readable form? Were the relevant accession numbers or links provided?	Not Applicable			
115			If publicly available data were reused, provide the respective data citations in the reference list.	Yes	References		
116							
