# Supplementary material for: Endothelial SLC25A13 links glutamine metabolism to angiogenesis
Source: EMBO Mol Med. 2026 Sep 26;18(11):4357–85. doi: 10.1038/s44321-026-00524-3 (PMC13748631; doi:10.1038/s44321-026-00524-3)
Supplement: Supplementary file 2 [file 44321_2026_524_MOESM8_ESM.pdf]

Expanded View Figures

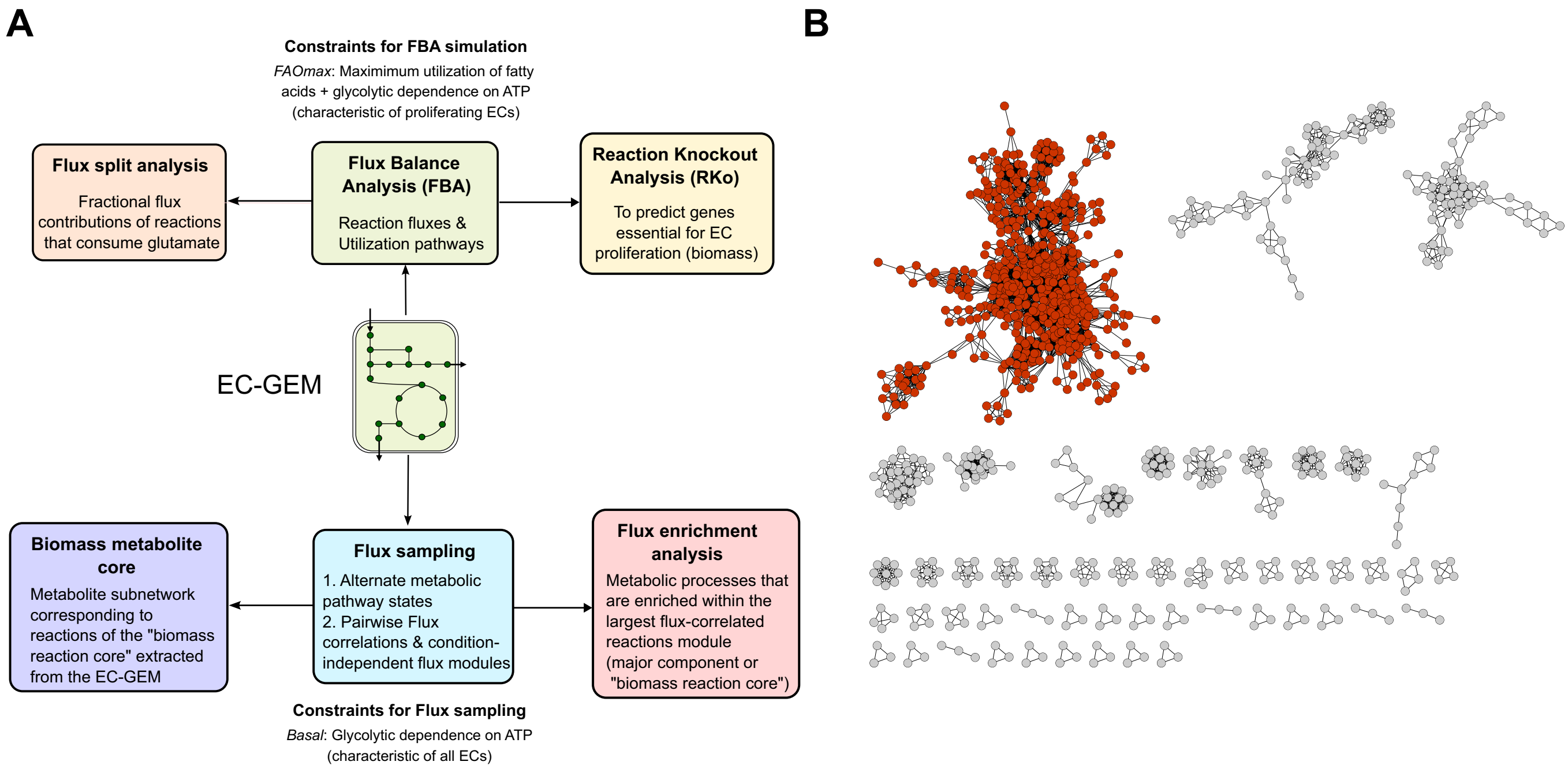

Figure EV1. Endothelial cell genome-scale metabolic model workflow.

(**A**) Workflow of the analyses performed using the endothelial cell genome-scale metabolic model (EC-GEM). (**B**) Flux correlation analysis in the EC-GEM. The network plot shows 1064 flux-correlated reactions distributed into multiple modules (groups). The nodes (balls) represent the reactions, and the sticks (edges) represent the presence of a correlation between the nodes. The red-colored motif represents the biomass reaction core that contains 548 flux-correlated reactions belonging to pathways required for biomass synthesis in ECs. The gray-colored motifs are smaller connected components of flux-coupled reactions enlisted in Dataset EV1.

 © The Author(s)

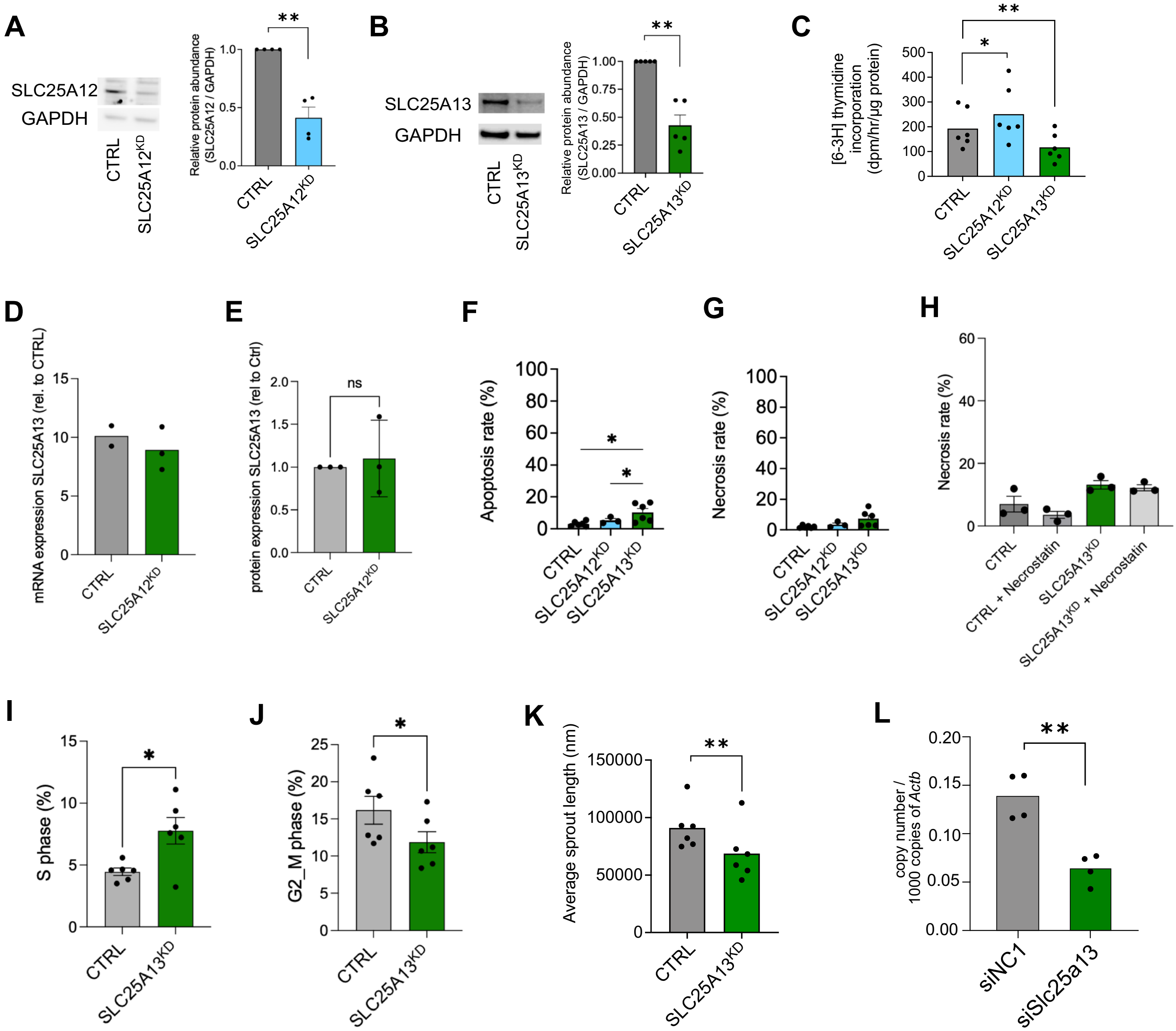

Figure EV2. Functional characterization of *SLC25A12* and *SLC25A13* knockdown in endothelial cells.

(**A**) Representative immunoblot and densitometric quantification of SLC25A12 protein levels in CTRL and SLC25A12^{KD} ECs (expressed relative to control) ($n = 4$). GAPDH was used as a loading control. (**B**) Representative immunoblot and densitometric quantification of SLC25A13 protein levels in CTRL and SLC25A13^{KD} ECs (expressed relative to control) ($n = 4$ and $n = 5$, respectively). GAPDH was used as a loading control. (**C**) Proliferation ([6-^{3}H] thymidine incorporation into DNA) of CTRL, SLC25A12^{KD}, and SLC25A13^{KD} ECs ($n = 6$). These results were obtained in the same experiment as the data shown in Fig. 2A and therefore show the same results for the CTRL. The results for the SLC25A13^{KD} bar have been repeated for an easy comparison. (**D**) Quantification of *SLC25A13* mRNA expression levels in CTRL and SLC25A12^{KD} ECs (expressed relative to control) ($n = 2$–3). (**E**) Densitometric quantification of SLC25A13 protein levels in CTRL and SLC25A12^{KD} ECs (expressed relative to control) ($n = 3$). (**F**) Quantification of apoptosis with Annexin/PI test ($n = 3$–6). (**G**) Quantification of necrosis with Annexin/PI test. (**H**) Quantification of necroptosis with Annexin/PI test ($n = 3$–6). (**I**) Quantification of cell cycle S-phase ($n = 6$). (**J**) Quantification of cell cycle G2_M-phase ($n = 6$). (**K**) Spheroid sprouting in mitomycin C-treated mitotically inactivated EC: sprout length (nm) of sprouting CTRL and SLC25A13^{KD} EC spheroids ($n = 6$). (**L**) Transcript levels of *Slc25a13* in ECs isolated from siNC1 or siSLC25A13 treated neovascularized choroids ($n = 4$). Data are means ± SEM; $^{*}P < 0.05$, $^{**}P < 0.01$ by one-sample *t* test and Wilcoxon test (**A**, **B**); a two-tailed paired *t* test (**C**, **E**, **F**, **G**, **I–K**); by a one-way ANOVA (**H**); unpaired *t* test (**L**). Exact *P* values: (**A**) $P = 0.0077$; (**B**) $P = 0.0037$; (**C**) CTRL vs SLC25A13, $P = 0.0015$; CTRL vs SLC25A12, $P = 0.0235$; (**F**) CTRL vs SLC25A13, $P = 0.0270$; SLC25A12 vs SLC25A13, $P = 0.0364$; (**I**) $P = 0.0179$; (**J**) $P = 0.0169$; (**K**) $P = 0.0096$; (**L**) $P = 0.0020$.

© The Author(s)

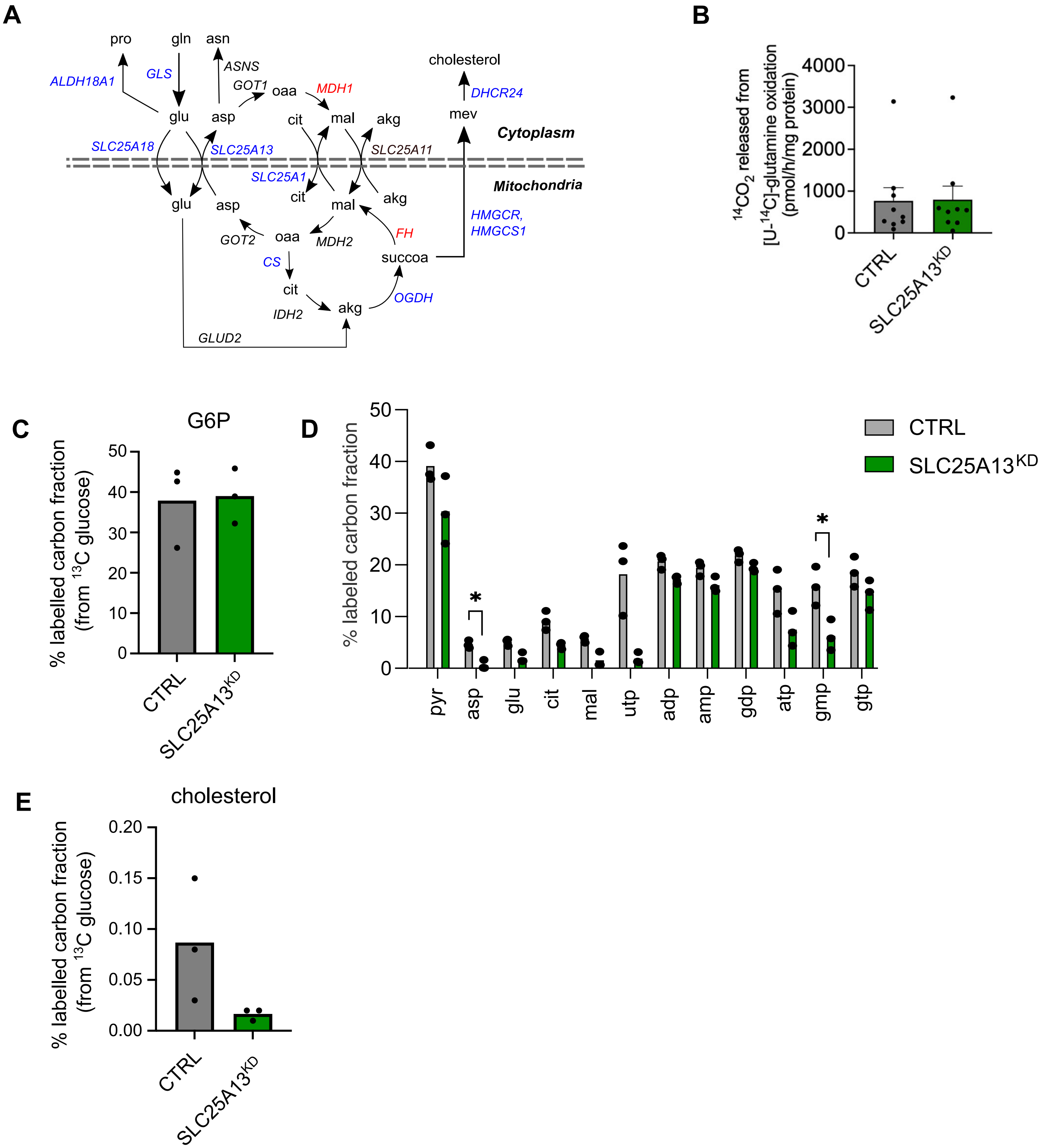

Figure EV3. ***SLC25A13* knockdown alters malate-aspartate shuttle activity and cellular carbon metabolism.**

(**A**) Scheme demonstrating the metabolic reactions associated with the malate–aspartate shuttle. Genes associated with these reactions are displayed on the reaction arrows. Blue colored gene names represent genes that are downregulated, and red-colored gene names represent genes that are upregulated upon SLC25A13^{KD}. (**B**) Glutamine oxidation: Amount of $^{14}CO_2$ released as a consequence of glutamine oxidation in CTRL and SLC25A13^{KD} ECs ($n = 9$). (**C**) Carbon fraction of glucose-6-phosphate (G6P) labeled from U-^{13}C glucose ($n = 3$). (**D**) Barplots representing the total fraction of [U-^{13}C]-glucose carbon in pyruvate (pyr), amino acids (asp, glu), TCA metabolites (cit, mal), nucleotides (ump, adp, amp, gdp, atp, gmp, gtp), and cholesterol precursor (mev) ($n = 3$). (**E**) Carbon fraction of cholesterol labeled from U-^{13}C glucose ($n = 3$). adp adenosine diphosphate, atp adenosine triphosphate, amp adenosine monophosphate, asp aspartate, cit citrate, gdp guanosine diphosphate, glu glutamate, gmp guanosine monophosphate, mal malate, pyr pyruvate, utp uridine triphosphate. Data are means ± SEM; not significant, $^{*}P < 0.05$ by a two-tailed paired t test (**D**). Exact P values: (**D**) asp, $P = 0.044900$; gmp, $P = 0.030079$.

 © The Author(s)

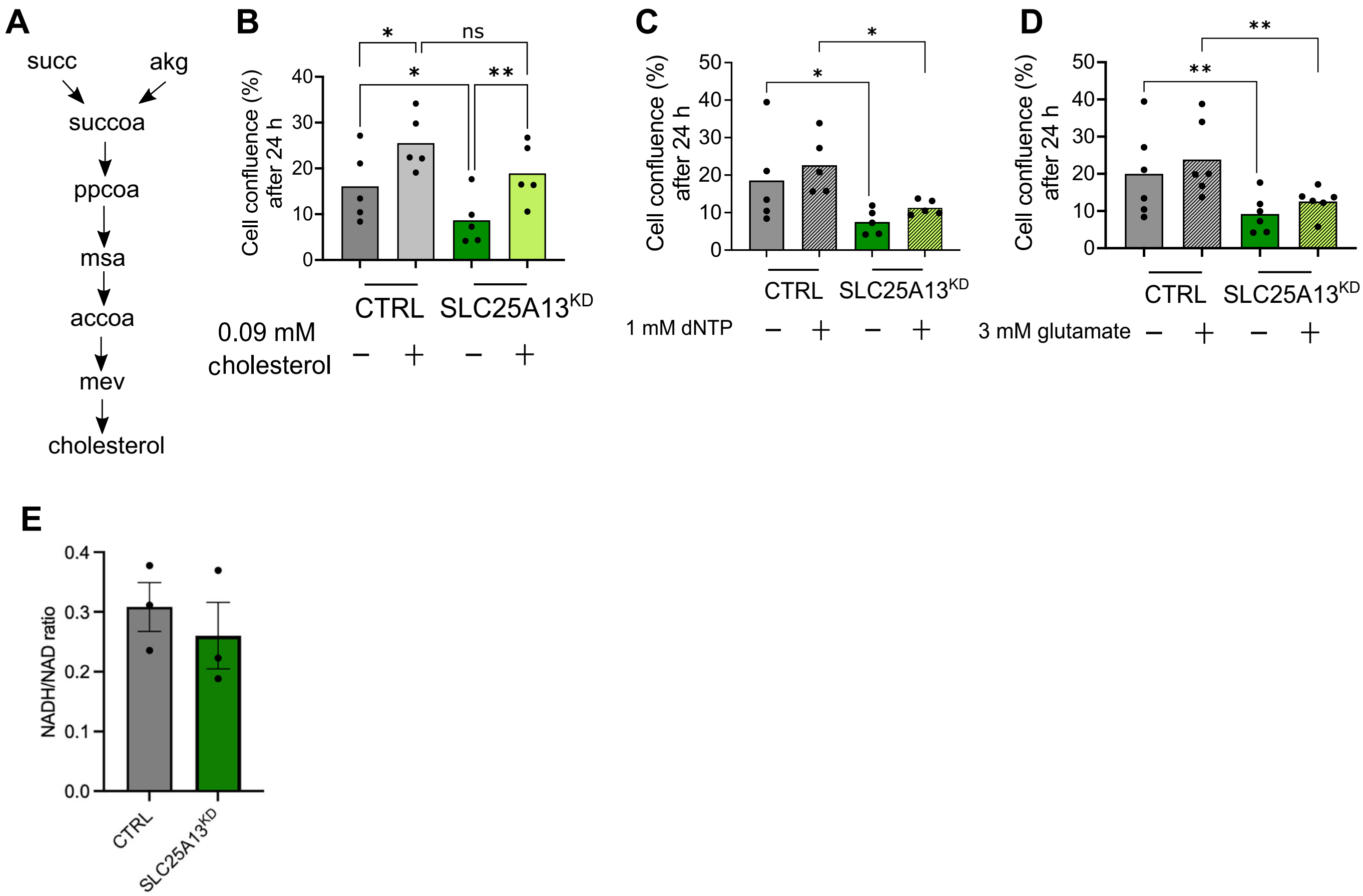

Figure EV4. SLC25A13 knockdown limits endothelial cell proliferation through impaired cholesterol, nucleotide, and glutamate metabolism.

(**A**) Pathway showing the catabolism of TCA metabolites to form mevalonate and hence, cholesterol. (**B**) Rescue with excess cholesterol: Barplots demonstrating the comparison between CTRL and SLC25A13^{KD} ECs grown in EGM2 + 0.09 mM methyl-β-cyclodextrin cholesterol ($n = 5$). (**C**) Proliferation (measured via percentage of cell confluency) of CTRL and SLC25A13^{KD} ECs, cultured in the presence or absence of 1 mM dNTP ($n = 5$). (**D**) Proliferation (measured via percentage confluence) of CTRL and SLC25A13^{KD} ECs, cultured with or without excess glutamate (3 mM) ($n = 6$). (**E**) Cellular NADH/NAD$^+$ ratio in CTRL and SLC25A13^{KD} ECs ($n = 3$). accoa acetyl-coA, akg α-ketoglutarate, mev mevalonate, msa malonate semialdehyde, ppcoa propionyl-coA, succ succinate, succoa succinyl-coA. Data are means ± SEM; ns, not significant, *$P < 0.05$, **$P < 0.01$ by one-way ANOVA (**B**–**D**). Exact P values: (**B**) CTRL (−) vs CTRL (+), $P = 0.0143$; CTRL (−) vs SLC25A13^{KD} (−), $P = 0.0435$; SLC25A13^{KD} (−) vs SLC25A13^{KD} (+), $P = 0.090$; (**C**) CTRL (−) vs SLC25A13^{KD} (−), $P = 0.0121$; CTRL (+) vs SLC25A13^{KD} (+), $P = 0.0104$; (**D**) CTRL (−) vs SLC25A13^{KD} (−), $P = 0.0075$; CTRL (+) vs SLC25A13^{KD} (+), $P = 0.0056$.

© The Author(s)

A

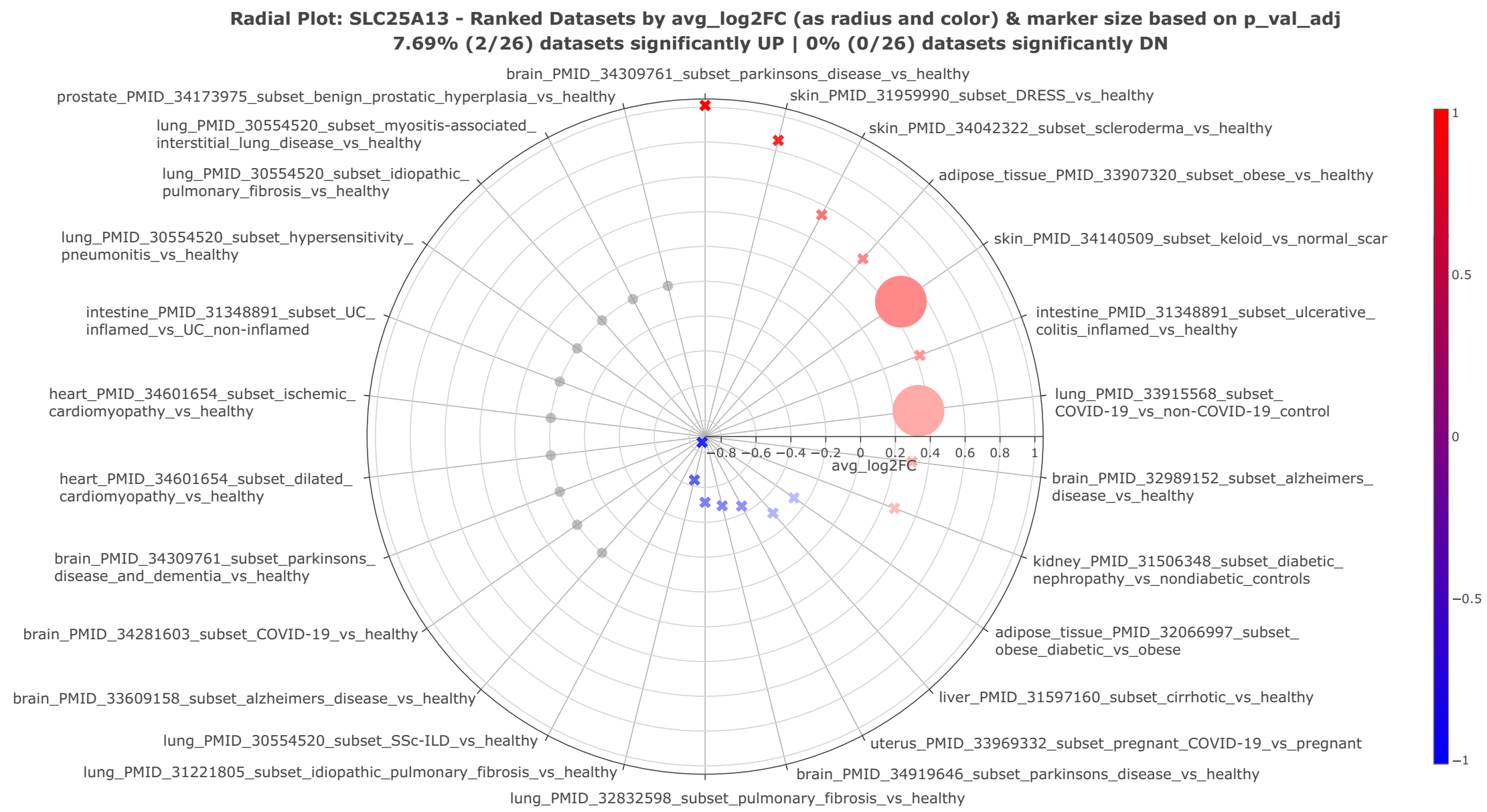

B

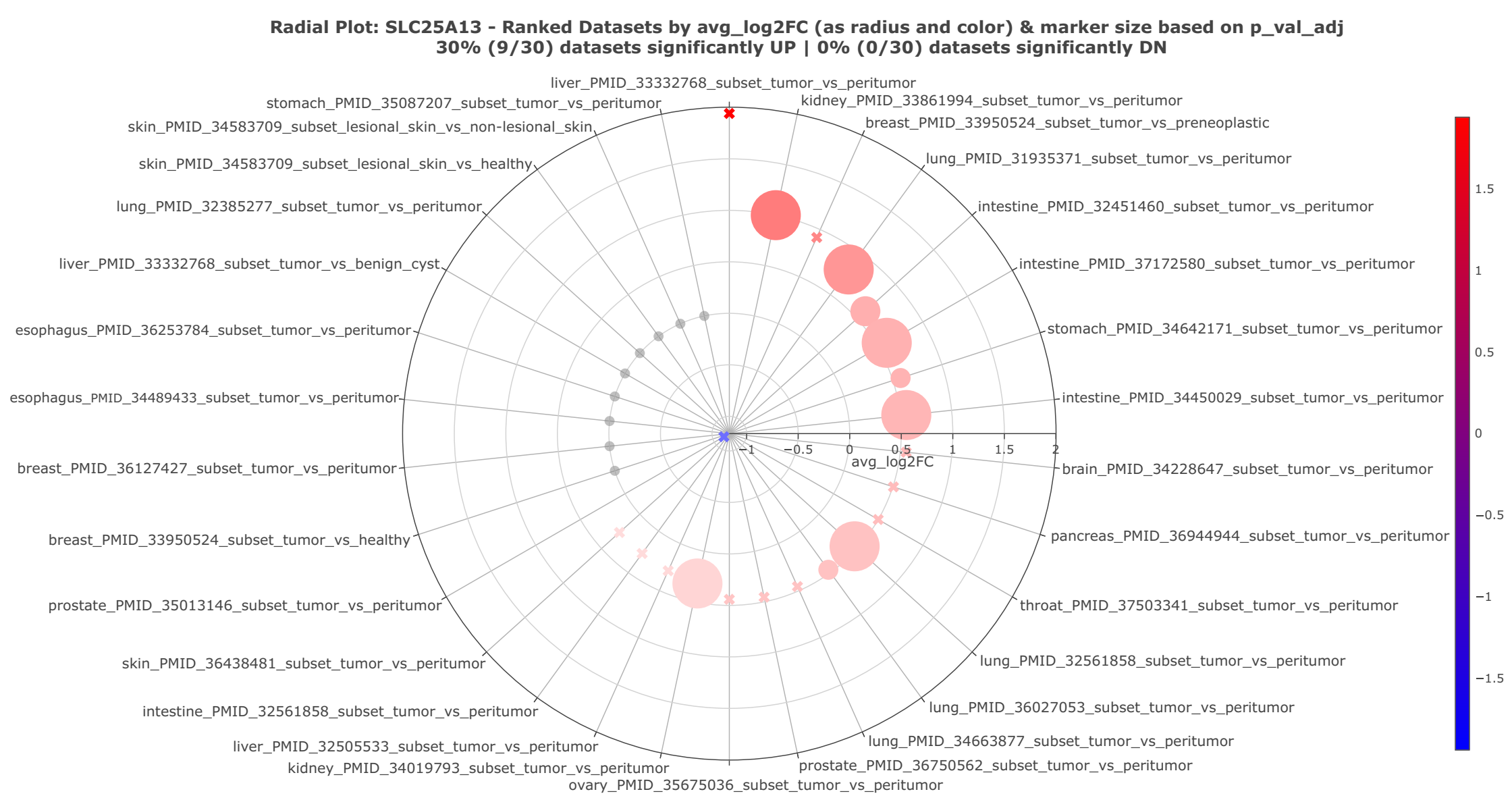

C

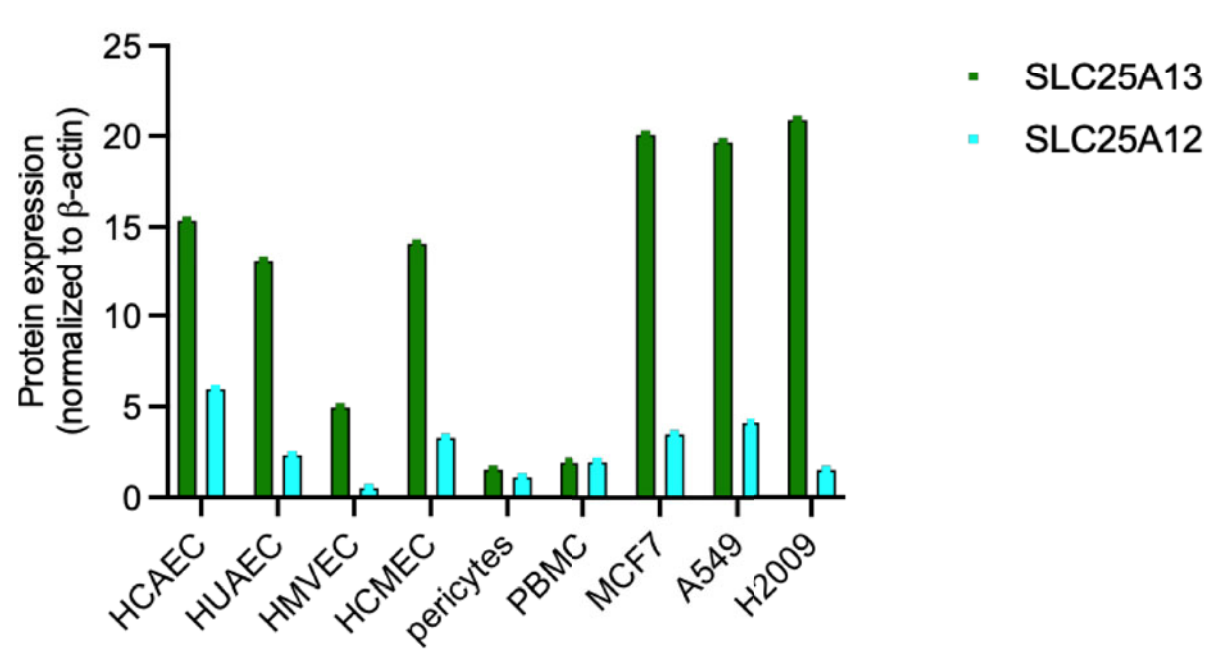

© The Author(s)

Figure EV5. SLC25A13 expression across inflammatory and tumor datasets and vascular cell types.

(**A**) Radial plot of *SLC25A13* expression in inflammatory datasets. (**B**) Radial plot of *SLC25A13* expression in tumor datasets. (**C**) SLC25A13 and SLC25A12 protein expression normalized to β-actin in vascular and non-vascular cells.

© The Author(s)
